# Nanochitin: Chemistry, Structure, Assembly, and Applications

**DOI:** 10.1021/acs.chemrev.2c00125

**Published:** 2022-06-02

**Authors:** Long Bai, Liang Liu, Marianelly Esquivel, Blaise L. Tardy, Siqi Huan, Xun Niu, Shouxin Liu, Guihua Yang, Yimin Fan, Orlando J. Rojas

**Affiliations:** † Key Laboratory of Bio-based Material Science & Technology (Ministry of Education), Northeast Forestry University, Harbin 150040, P.R. China; ‡ Bioproducts Institute, Department of Chemical & Biological Engineering, Department of Chemistry, and Department of Wood Science, 2360 East Mall, The University of British Columbia, Vancouver, BC V6T 1Z3, Canada; § Jiangsu Co-Innovation Center of Efficient Processing and Utilization of Forest Resources, Jiangsu Key Lab of Biomass-Based Green Fuel and Chemicals, College of Chemical Engineering, Nanjing Forestry University, 159 Longpan Road, Nanjing 210037, P.R. China; ∥ Polymer Research Laboratory, Department of Chemistry, National University of Costa Rica, Heredia 3000, Costa Rica; ⊥ Department of Bioproducts and Biosystems, School of Chemical Engineering, Aalto University, FI-00076 Aalto, Finland; # Department of Chemical Engineering, Khalifa University, Abu Dhabi, United Arab Emirates; ∇ State Key Laboratory of Biobased Material and Green Papermaking, Qilu University of Technology, Shandong Academy of Sciences, Jinan 250353, China

## Abstract

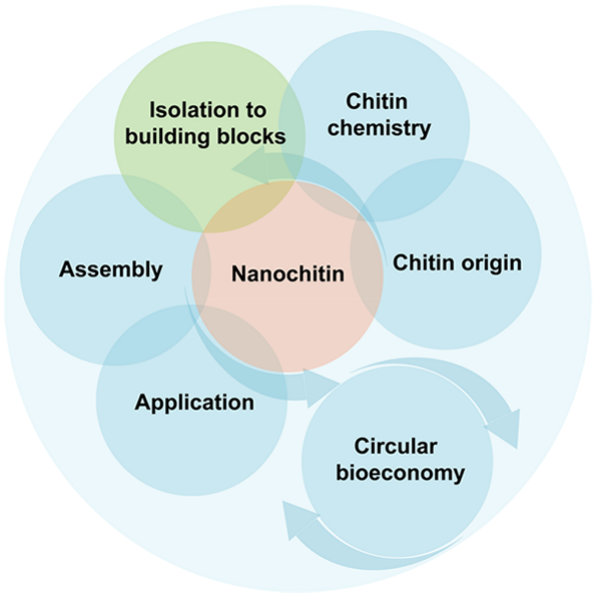

Chitin, a fascinating biopolymer found in living organisms, fulfills current demands of availability, sustainability, biocompatibility, biodegradability, functionality, and renewability. A feature of chitin is its ability to structure into hierarchical assemblies, spanning the nano- and macroscales, imparting toughness and resistance (chemical, biological, among others) to multicomponent materials as well as adding adaptability, tunability, and versatility. Retaining the inherent structural characteristics of chitin and its colloidal features in dispersed media has been central to its use, considering it as a building block for the construction of emerging materials. Top-down chitin designs have been reported and differentiate from the traditional molecular-level, bottom-up synthesis and assembly for material development. Such topics are the focus of this Review, which also covers the origins and biological characteristics of chitin and their influence on the morphological and physical-chemical properties. We discuss recent achievements in the isolation, deconstruction, and fractionation of chitin nanostructures of varying axial aspects (nanofibrils and nanorods) along with methods for their modification and assembly into functional materials. We highlight the role of nanochitin in its native architecture and as a component of materials subjected to multiscale interactions, leading to highly dynamic and functional structures. We introduce the most recent advances in the applications of nanochitin-derived materials and industrialization efforts, following green manufacturing principles. Finally, we offer a critical perspective about the adoption of nanochitin in the context of advanced, sustainable materials.

## 1 Nanochitin and Its Sustainability

### 1.1 Nanochitin as a Renewable Material

Society is witnessing a global impetus toward regenerative systems that move us closer to the goals of the bioeconomy and fast-changing materials production models,^1^ directing to improve the quality of life while minimizing or preventing negative impacts on the environment. This requires a shift of material screening from fossil sources to biomass ones,^2^ wherein accessible bioresources are critical. The ocean ecosystem, covering 71% of Earth’s total surface and containing 97% of all water on our planet, is a source of life^3,4^ and emerges as an “unlimited” bioresource.^5^ This is particularly true as the development of aquaculture overcomes many of the issues that are associated with agricultural land use, which can eventually reach saturation. The biomass found in the ocean possesses unique structures compared to that in land organisms;^6,7^ however, it represents a minor subject in the field of natural biopolymers, vastly outweighed by the popularity of plant-based resources. An exception is the case of biopolymers sourced from algae, which can be envisioned as an active area in future ocean-based biorefineries. On the other hand, chitin is the most abundant biopolymer in the ocean, principally present in the exoskeletons of crustaceans.^8,9^ Annually, 6–8 million tons of waste crab, shrimp, and lobster shells are produced globally,^10^ particularly from Asian counties.^11^ Basically, chitin is a long-chain polymer of (1,4)-β-*N*-acetylglucosamine, a derivative of glucose,^12,13^ making it a major precursor of cationic polysaccharides from nature, and exhibits biological activity, biocompatibility, biodegradability, low allergenicity, and easy processability.^14−17^

**Figure 1:**
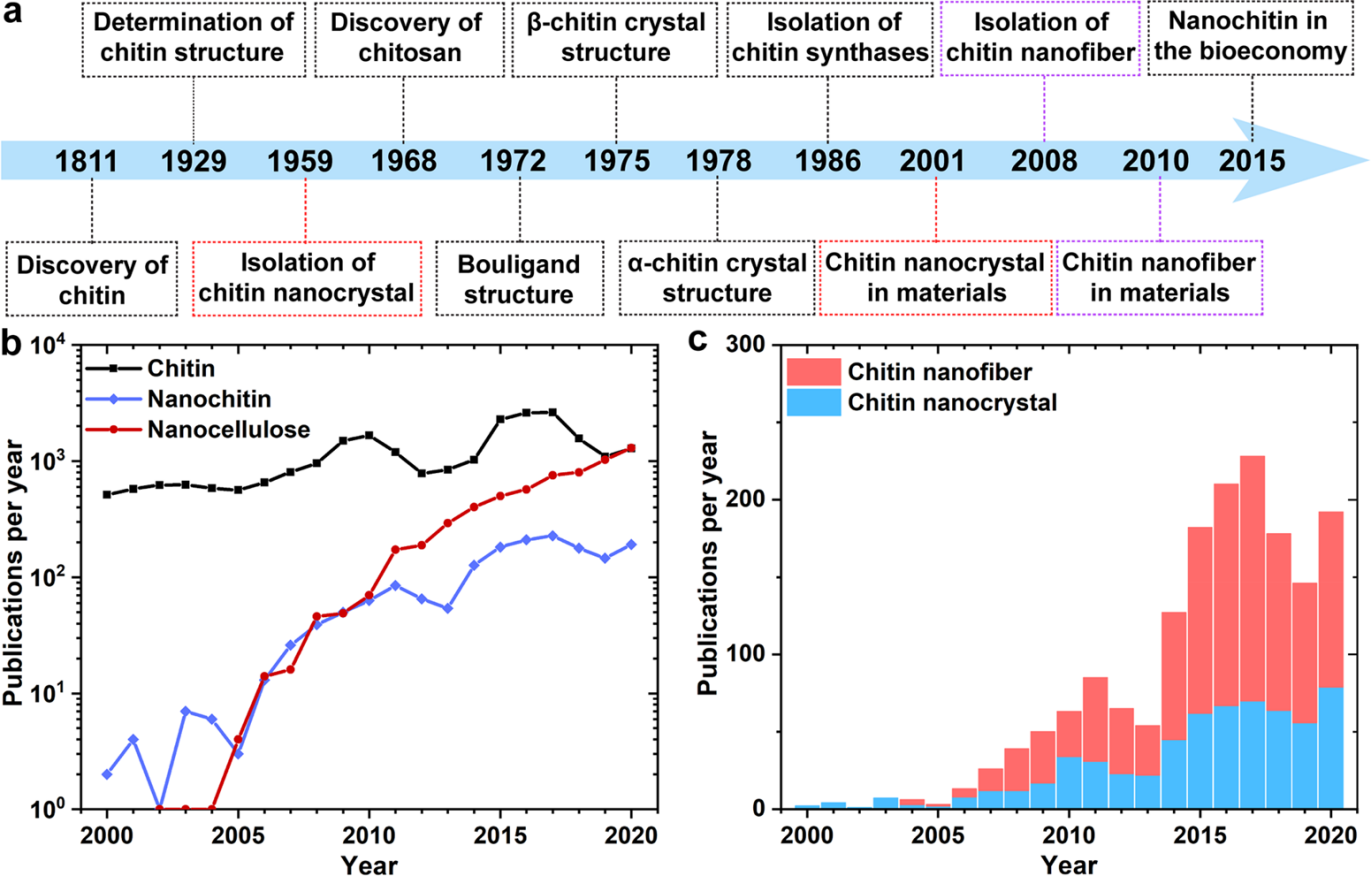
(a) Timeline of discovery, analysis, extraction, use, and future direction of chitin and nanochitin. Red and purple dashed frames indicate chitin nanocrystal and nanofiber, respectively. Adapted with permission from ref (19). Copyright 2018 Elsevier. Summary of publications relating to (b) chitin, nanochitin, and nanocellulose and (c) comparison of chitin nanofibers and nanocrystals. The number of publications was collected from the Science and Engineering Indicators of the National Science Foundation between 2000 and 2020 using Scopus. The research was limited to titles, abstracts, and keywords as follows: chitin/cellulose (*) nanocrystal and nanofiber, including * nanowhisker, * whisker, * nanorod, * *crystallite*, nanocrystalline *, * nanofiber, * nanofibril, nanofibrillar *, and nanofibrillated *.

Despite its many attractive features, chitin remains among the least utilized biomass resource given its intractable structure and recalcitrance toward processing.^18^ Indeed, after its discovery over two centuries ago,^19^ reports on chitin utilization is only dated in recent decades (Figure 1a). Chitin is mostly used in its highly deacetylated (degraded) form, known as chitosan, which shows good solubility and processability in acidic conditions.^20^ However, the abundant waste produced in chitosan production can be seen as a limitation to the sustainable use of chitin,^21^ which justifies the consideration of new strategies with better environmental and economic prospects. Thus, direct processing and conversion of chitin into novel building blocks, fitting material design and creation, represent a green and well justified alternative. To this end, one can take advantage of the structural characteristics of chitin at the nanoscale, referred in this Review as “nanochitin”. Nanochitin is the elementary building block of chitin structures, more precisely, hierarchical mesostructures, featuring similar chemical and biological reactivity compared to the chitin precursor. Understanding the structural construction of nanochitin in nature, as well as its chemical adaptability, will facilitate its adoption toward performance optimization and inspire the design of novel engineered materials. Considering the annual rate of publications in the area, research related to nanochitin follows a much slower rate compared with that of the parent chitin (Figure 1b). Therefore, the characteristics of nanochitin, particularly from the material perspective, remain a topic for upcoming discoveries.

### 1.2 Nanochitin Characteristics

Structurally, nanochitin is considered as an assembly of semicrystalline chitin nanofibrils that show highly oriented nanocrystals embedded in an amorphous matrix, wherein single nanofibrils are packed into highly orientated microfibrils or fibril bundles that are held together by van der Waals (vdW) forces and hydrogen bonding (H-bonding).^22^ The attractive properties of nanochitin stem partially from its fibrillar or rodlike nature, nanometer lateral dimension, and tailorable crystallinity, among others. Chemically, nanochitin is confined by a sheath of proteins and is assembled into elongated fibrils that are embedded in a mineral–protein matrix in the exoskeleton of arthropods. Hence, removal of proteins and minerals is a necessary step prior to nanochitin isolation. Unlike other forms of natural nanopolysaccharides, nanochitin contains nitrogen, which is crucial for life.^23^ The amino groups also endow nanochitin with versatile processing and a vast number of applications that are not typical of their nanopolysaccharide counterparts.

In this Review, we consider two types of nanochitin, chitin nanofiber (ChNF) and nanocrystal (ChNC), which have equally attracted research interests in recent years (Figure 1c). To keep the inherent native crystalline structure, the most common route to access nanochitin is direct disintegration of chitin bundles. Hence, unlike most synthetic nanoparticles, nanochitin is produced top-down and preserves, to some degree, the biological morphology and ordered structure in shells. Thus, different from the regenerated chitin nanomaterials based on the dissolved chitin,^18^ nanochitin maintains a sophisticated meso-architecture that reflects that of its source, endowing the possibility for reconstruction into structures that take advantage of the features of “nanosized units” essential for nanotechnology as a field.^19^

The processing methods used to produce nanochain greatly influence its characteristics. ChNC is typically produced by controlled chemical reactions that selectively remove the disordered chitin segments present in the source material, leaving chitin crystallites intact.^24^ Upon processing, surface charges, either positive or negative, can be installed on the ChNC surface, which ensures colloidal stability in aqueous media. The resultant nanoparticles can be considered as rigid nanorods (highly crystalline chitin assemblies),^25^ akin to cellulose nanocrystals (CNC).^26,27^ ChNF, on the other hand, is obtained by disintegrating native chitin using mechanical treatments without removal of the disordered, noncrystalline regions. When no surface chemical treatment is applied to native chitin, mechanical shearing, even under intense conditions, results in ChNF that presents heterogeneously distributed lateral dimensions. On the contrary, more individualized ChNF, *e.g.*, with relatively uniform lateral dimensions, can be achieved by mechanically disintegrating chitin after chemical pretreatment, wherein enhanced interfibrillar repulsion exists given the ionized nanofibril surface. Important features of ChNF include its high axial aspect ratio and flexibility, which promote entanglement and, together with hydration and electrostatic interactions (if present), facilitate the formation of hydrogels at low concentrations.

In the past two decades, nanochitin has increasingly captured the attention of academia, as evidenced by the rapid publication growth (Figure 1b); nevertheless, it still lags behind other biomass-based nanoparticles, such as nanocelluloses,^28^ particularly in the recent decade (Figure 1b). The main reason for this latter observation is likely related to the fact that although chitin shares many similarities with cellulose, the hydroxyl groups in chitin are partially occupied with acetyl amine, resulting in stronger H-bonding between the neighboring chains.^29^ This makes chitin more stable and more difficult to be disintegrated or isolated than cellulose, significantly affecting access and conversion of chitin to nanochitin, not to mention that the acceptability of nanochitin in different cultures influences its utilization and commercialization. To conclude, nanochitin has been less popular compared to other renewable nanoparticles but shows significant attractiveness given its remarkable potential, which deserves a systematic investigation in the future and is the main subject of this Review.

### 1.3 Scope of This Review

**Figure 2:**
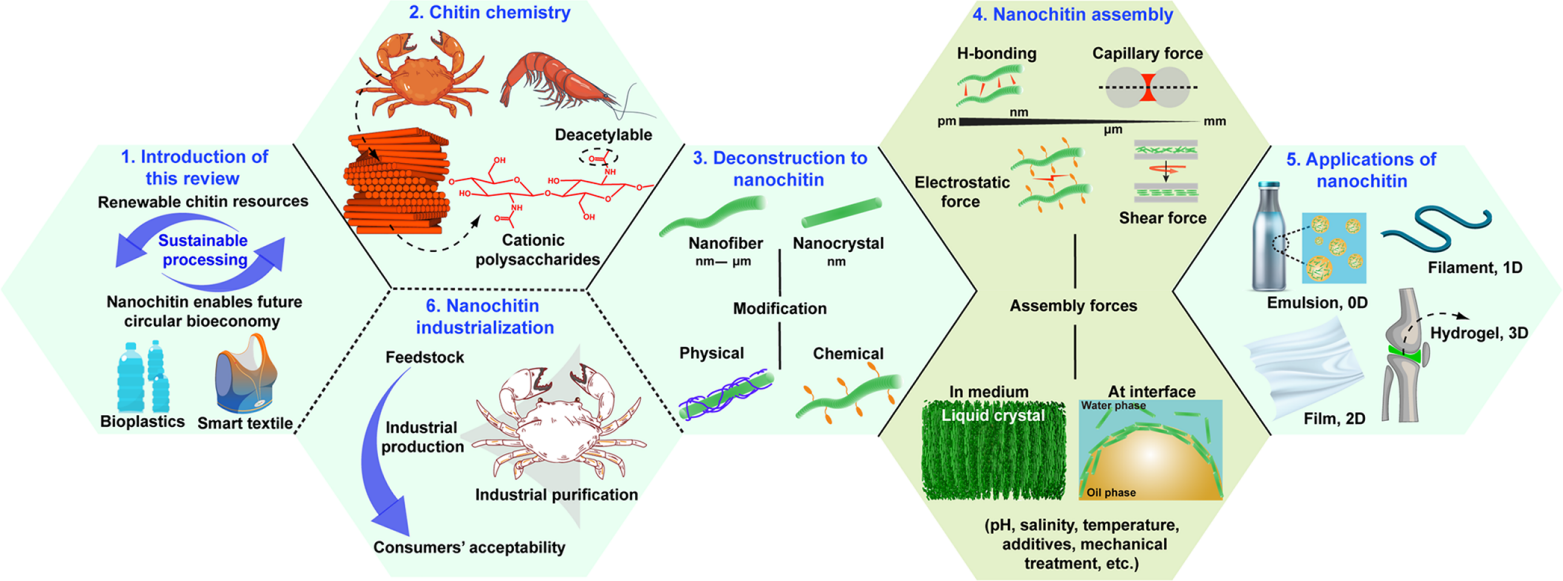
Structure of this Review, which includes a brief introduction (panel 1, section 1) and introduces the relationship between chitin chemistry (section 2) and its deconstruction into nanochitin (panel 3, section 3). The Review also covers the main interactions (panel 4, section 4) that rule the multiscale structuring of nanochitin in a variety of media, resulting in diverse applications. The latter take advantage of multidimensional phenomena (panel 5, section 5). Finally, we present the promise of nanochitin in relation to industrialization and to accelerate materials development in the context of the bioeconomy (panel 6, section 6).

We review the topic of nanochitin as a source of sustainable materials, following the general structure schematically illustrated in Figure 2. We highlight the use of nanochitin to improve the accessibility and utilization of ocean and other resources and emphasize a performance that matches or surpasses that of other biomass-based nanomaterials (Figure 2, panel 1). Several recent reviews have considered nanochitin, mostly focusing on its application and covering topics related to material development and associated physicochemical properties. Here, we comprehensively discuss aspects related to nanochitin colloidal interactions and assemblies to develop new functions. We address the possibility of harnessing the fundamental chemical and structural characteristics of nanochitin to guide material assembly, design, and development, highlighting the significance and uniqueness of each involved step, including isolation, modification, processing, reconstruction, and industrialization. Particularly, we refer to advances in nanochitin produced from top-down strategies, acknowledging bottom-up synthesis routes.^18,30^ Herein, we first discuss the origin and biological and chemical features of chitin, which are key aspects relevant to its deconstruction into nanochitin, giving an accent to the superiority of top-down approaches and highlighting the adaptability of nanochitin (Figure 2, panels 2 and 3). The assembly interactions that take part in the multiscale structuring of nanochitin in a variety of media are then summarized (Figure 2, panel 4), bearing in mind that processing conditions greatly influence the suprastructures of assembled nanochitin. We further show the potential of nanochitin as a building block of multidimensional bioproducts and discuss the relevant structure–process–property relationships, considering dynamic assembly strategies (Figure 2, panel 5). We close the Review by discussing upcycling and industrialization opportunities with an eye on the future, taking (nano)chitin as an enabler for circular and sustainable manufacturing (Figure 2, panel 6).

## 2 Origin, Biological, and Chemical Features of Chitin

### 2.1 Biological Origins of Chitin

**Figure 3:**
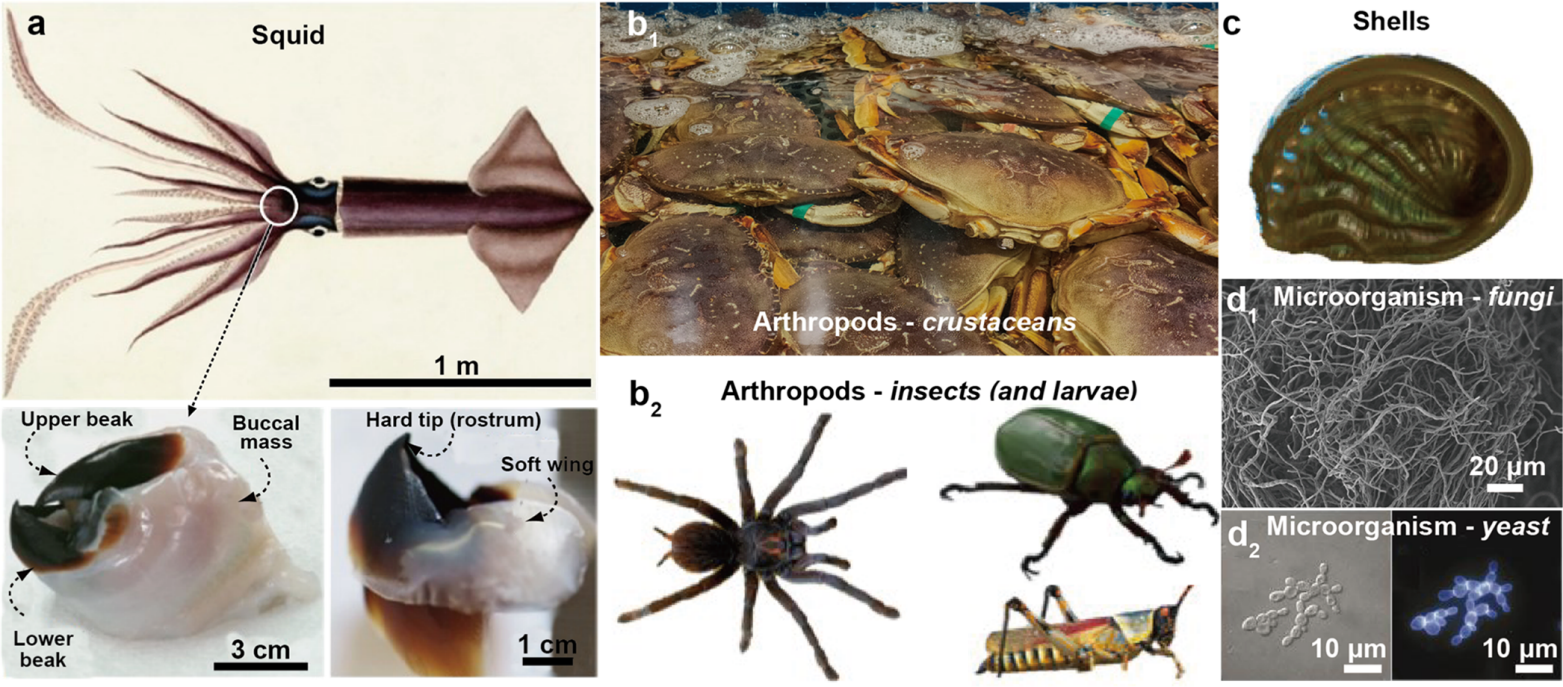
Variety of organisms associated with chitin, including, to cite only a few examples: (a) Squid, typically in the beak and pens. Adapted with permission from ref (37). Copyright 2008 American Association for Advancement of Science. Arthropods including (b_1_) crustaceans and (b2) insects and their larvae. (c) Shells, from terrestrial, river, or sea mollusks. Microorganisms including (d1) fungi and (d2_)_ yeast. The dispersed mycelium in d1 is harvested from *Aspergillus niger*, and the yeast pseudohyphae in d2 is stained by Calcofluor white. Adapted with permission from refs (38) and (39). Copyright 2021 and 2002, respectively, Springer Nature.

Chitin makes up parts of many different organisms with unique molecular structures (Figure 3).^31^ In this subsection, chitin sources are introduced along with their main features. Although different classes of organisms, *e.g*., terrestrial or marine, fungi, or insects, are considered in many instances, a small subset of species has been explored in the context of chitosan and chitin valorization. In most cases, these are associated with waste streams such as silk-work larvae, seafood byproducts, or simply processing food-waste derived from insect biomass.^8,32^ However, there is a very large set of chitin-synthesizing organisms, namely, nearly all insects, fungi, crustaceans, seashells, and mollusks. These organisms use chitin for a range of functions that include scaffolding during growth, mechanical strength and toughness, controlled opacity and light reflection, adhesion, and communication and as a first line of defense against microorganisms and pathological stress.^33−36^ Herein, we introduce the biosynthesis of chitin and chitin suprastructures found in organisms. Regarding chitin metabolism, superstructures, and functions, it is important to note that insects are most studied since they are easily accessible and common subjects of developmental biology. Furthermore, their body parts do not present a high mineral content compared to crustaceans or seashells, enabling a more facile identification of chitin-related intricate structures.

#### 2.1.1 Chitin’s Multiscale Structures and Functions

Chitin nanofibrils are arranged following a wide variety of biological architectures. The arrangement of nanofibrils enable tethering of a range of macroscopically observable properties. This includes networks of chitin and proteins in the exoskeleton of arthropods to achieve a remarkable combination of strength and toughness. However, chitin nanofibrils also form structures used for adhesion, enabling adhesion to a wide range of surfaces that support the full animal body weight or, for specific reflection of light, in a palette that finds no match in current synthetic colors. We introduce these aspects as a premise to reverse engineering chitin nanofibrils.

##### 2.1.1.1 Nanochitin Structures and Mechanical Resilience

**Figure 4:**
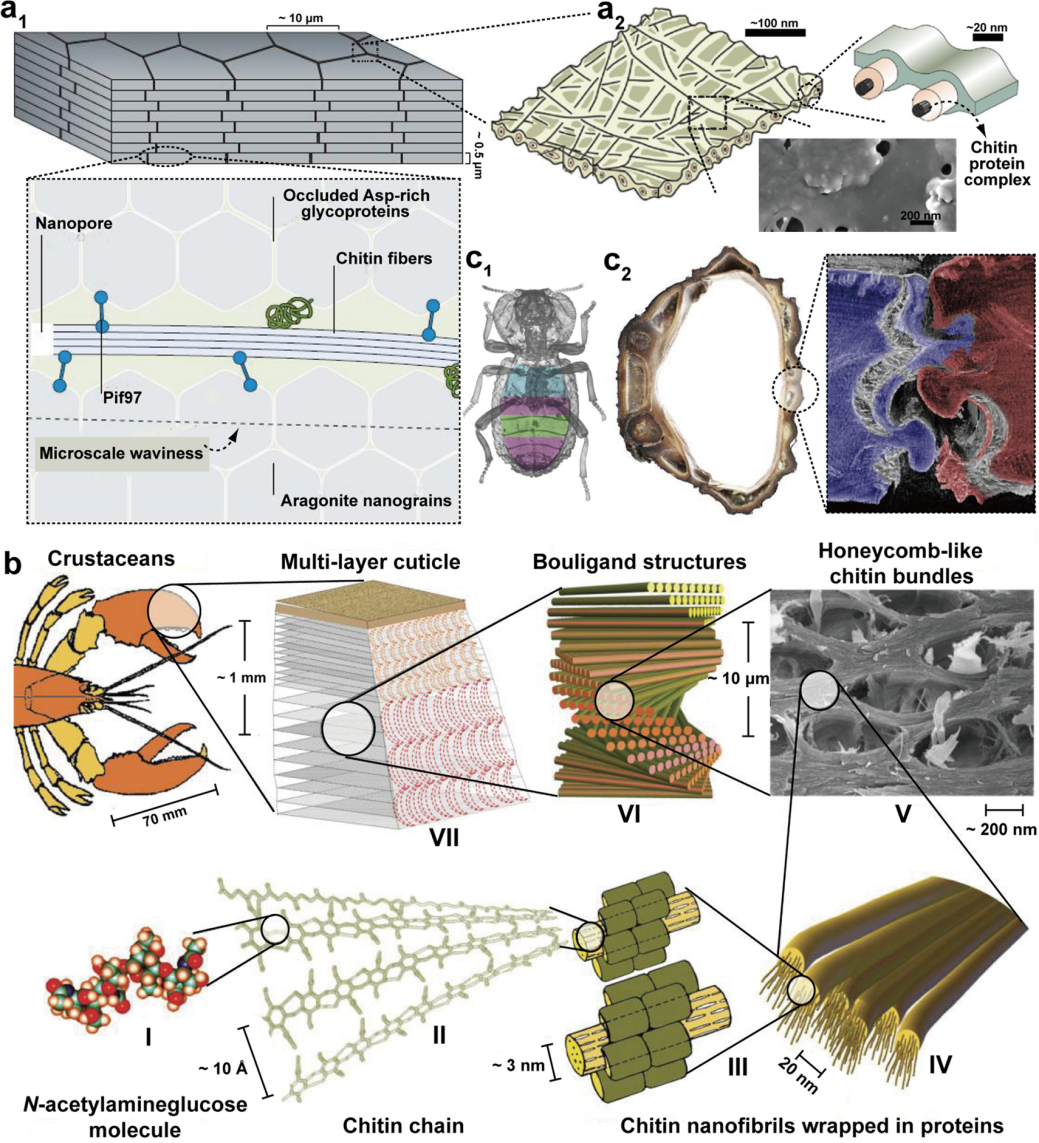
Examples of multiscaled chitinous structures present in biological constructs. (a1) Mineral platelets organized within nacreous structures, *e.g.*, in seashells, wherein the plates are cemented by calcite–protein–chitin complexes. Adapted with permission from ref (49). Copyright 2016 Springer Nature. (a2) Chitin fibril bundles within the protein matrix between inorganic plates. Adapted with permission from ref (60). Copyright 2021 American Chemical Society. (b) Multiscaled structures found in crustaceans. The monomeric unit (I) is polymerized into chitin polymers (II), which form bundles within proteins with specific binding sites (III). The bundles are arranged in a number of structures that optimize fracture deflection and strain-dependent response, herein exemplified with the honeycomb structure (V) and helicoidal Bouligand structures found at multiple scales within the cuticle (VI, VII). Adapted with permission from ref (44). Copyright 2010 John Wiley and Sons. (c1) Plane view of diabolical ironclad beetle, highlighting three distinct internal regions with variable spacing between organs and elytra. (c2) The jigsaw-type joints in the beetle yield a sequential manner, through delamination of the joints (right panel: computed tomography reconstruction of fractured suture), resulting in the highest resistance to compression in the animal kingdom. Adapted with permission from ref (53). Copyright 2020 Springer Nature.

When put to scale, chitin structures, particularly in insects, seashells, and crustaceans, possess an ideal combination of strength and toughness-to-body weight ratio. Most structures are expressed asbrick-and-mortar architectures, where energy dissipation occurs *via* a fracture and follows a tortuous path, *i.e*., energy dissipating along soft layers surrounding hard, generally minerals, *e.g*., calcium carbonate layers. Such architectures are typically observed in nacre or crustaceans (Figure 4a1)^40^ and have inspired many efforts in biomimicry.^41^ Although chitin is strong, with an axial tensile strength of the order of a gigaPascals,^42^ the chitin–protein bundles act as soft layers (Figure 4a2). Moreover, the interactions between individual components are extremely strong, contributing to overall mechanical toughness exhibited by the respective organisms. As determined by X-ray diffraction, the axial modulus of a dry α-chitin crystal has been estimated to be 41 GPa, making the fibers both strong and stiff in the axial direction.^43^ The axial Young’s modulus of such nanofibers is generally an order of magnitude higher than the transversal modulus, associated with the directionality of the fibrous networks with macroscopic stiffness. Importantly, the flexural anisotropy can be completely removed at the macroscopic scale by arranging the fibers hierarchically.^44^ In insects, chitin plays a role as hard component since there are no minerals.

Beyond nanoscaled brick-and-mortar architectures, there are a range of structures found in insects and crustaceans that elevate the potential for energy dissipation (Figure 4b).^45,46^ At each scale, these structures optimally dissipate various compressive, tensile, or flexural macroscopic stresses occurring from multiple directions. In comparison, collagen, silk, or cellulose nanofibrils (CNF) take up only relatively simple conformations, although also multiscaled.^22^ These simple conformations are aligned in the case of collagen or silk and twisted around the cell walls in the case of cellulose. Most of the chitinous architectures observed in arthropods present the so-called Bouligand structures. They correspond to stacked and rotated fibrous microstructures, forming lamellae or layers that resemble plywood (Figure 4b). Therein, aligned fibrils enable in-plane isotropy, and particularly, adjacent layers are progressively rotated with respect to their neighboring ones. This helicoidal architecture efficiently deflects fractures and cracks as a function of the twist angle, typically small (<10°), and results in three-dimensional dissipation of the stresses and higher angles to delamination. This type of hierarchical structure is generally convoluted with topographical motifs. Thereafter, the sheetlike helicoidal layers are curved into periodic structures or spheroidal contours.^47,48^ This is the case of the mantis shrimp that exhibits some of the best combinations of strength and toughness, as found in its hammerlike appendage. This added level of hierarchy enables the hammer of the mantis shrimp to easily fracture seashell nacre, although both are mineralized structures comprising proteins and chitin.^49^ Beyond Bouligand structures, a range of “lock-in” mechanisms can be found from chitinous architectures, where the radius of curvature within jigsawlike structures, combined with their tilt angle, provides optimal stress dissipation mechanisms.^50−52^ This has most recently been highlighted in the diabolical ironclad beetle that presents one of the most remarkable displays of resistance to compression in the animal kingdom while possessing a rather low density body (Figure 4c).^53^ This is achieved by combining a delaminating chitinous structure with jigsaw joints that help with transduction of the stresses across the joined interfaces. Lastly, a typical structure combined with the previously mentioned configurations is that of honeycomb, where chitin bundles are organized in an architecture within each of the layers, which can be arranged in Bouligand structures across planes. The stiffness of these planes can be modulated for efficiently dissipating in-plane stresses as a function of the ratio of chitin bundle thickness to the segment length of the hexagonal structures.^54^

In another implementation, macroscaled porosity within the chitin-based skeleton of the squid pen enables multiscaled buoyancy chambers, which can resist the varying hydrostatic pressures of the ocean.^55^ At the macroscale, a large range of architectures can be observed, with animal cales and armors being typical examples as well as patterned armor of certain animals.^56,57^ In the case of fungi, the diversity in chitin long-range order is considerably reduced with much less intricate designs. In contrast, in insects and crustaceans, many structures have been optimally designed to dissipate stress, relevant to the direct environment associated with the respective organisms. These structures are the subject of ongoing studies that aim to generate materials following the concepts of biomimicry, from the nano- to macroscale. At the nanoscale levels, liquid crystals (LCs) are used to reproduce related architectures. At the macroscale, interesting developments take place in designs for the automotive and aeronaval industries, which can take advantage of the physical and mechanical features found in nature,^50,58^ for instance, exemplified by the strategies used in mechanical fasteners, similar to those found in turbine engines or dovetails in turbine blades as well as landing gear fittings.^53,59^

##### 2.1.1.2 Nanochitin Structures and Selective Light Reflection

**Figure 5:**
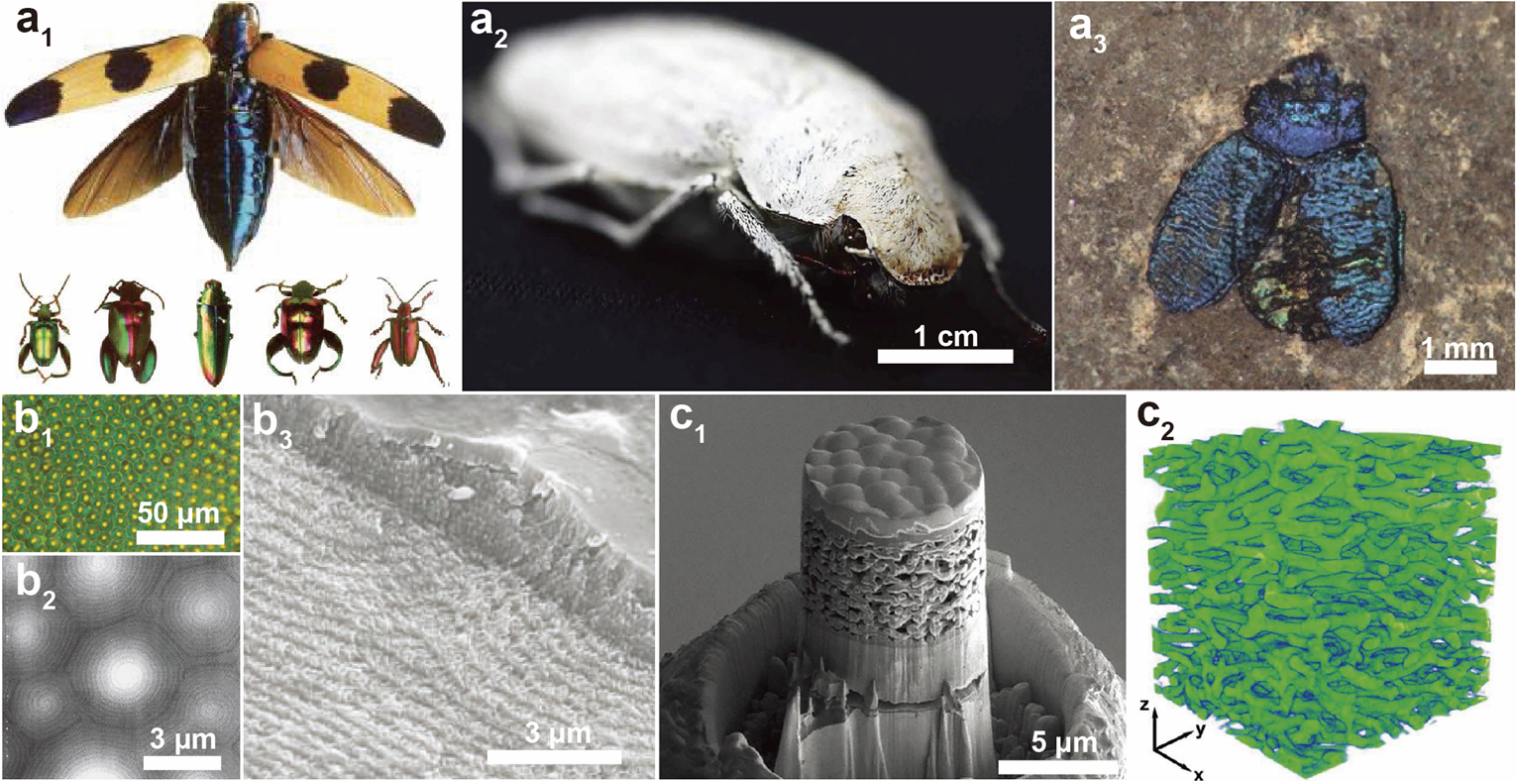
Chitin structures and associated colors found in beetles. (a1) Range of structural colors in various beetles. Adapted with permission from ref (74). Copyright 2008 John Wiley and Sons. (a2) Highly scattering shell of *Cyphochilus* sp. yielding a superwhite appearance. Adapted with permission from ref (72). Copyright 2018 John Wiley and Sons. (a3) Preserved 40 million year old beetle cuticle showing light (*Chrysomelids* from Eckfeld). Adapted with permission from ref (66) . Copyright 2012 The Royal Society. (b1) Optical micrograph of the exoskeleton of beetle *Chrysina gloriosa* showing bright yellow reflections from the core of each cell and greenish reflection from the edges. (b2) AFM and (b3) SEM images for the orientation of the Bouligand structures into semispheres in the epicuticle. Adapted with permission from ref (63). Copyright 2009 American Association for Advancement of Science. (c1) Cross-sectional SEM and (c2) 3D reconstruction of structures of white reflecting epicuticle as displayed in a3. Adapted with permission from ref (72). Copyright 2018 John Wiley and Sons.

In addition to the mechanical properties, the long-range order of chitin-based structures results in a wide range of optical properties, associated with structural color, where periodical features generate a broad range of optical phenomena (Figure 5a1 and 5b1), enabling color mixing, angle-dependent color (iridescence), circular polarization, and selective filters, among others.^61−64^ Color production is typically generated *via* pigmentation (typically from amino acids aromatic residues), interference, diffraction, and scattering or a combination of these four phenomena.^65^ Within very thin layers (<10 μm) present on given animals, these “colors” range from highly white (Figure 5a2) or black layers to broadband or very narrow band specific reflections (Figure 5a1). These reflections are considerably more resistant to fading than those obtained from pigments. Indeed, fossils that are a million years old still show high intensity reflections (Figure 5a3).^66^ While the wings of butterflies generally result in colors by a combination of phenomena, the reflections from beetles or crustaceans are more typically associated with light interference and that of the moth is associated with diffraction gratings.^65^ In recent days, interference is the most studied case, principally from films or layered structures. Specific iridescent reflections, where color display is angle-dependent, are a result of such interference phenomena.^67^ Nanostructured fibrils present in the chitin-containing species are organized into hierarchical, periodic thin films structures with scales matching the visible spectrum, between ca. 300 and 800 nm.^68^ These films can be curved, which results in direction-controlled color where the refractive index (RI) difference is generated between the chitin fibrils and that of the surrounding matrix. Although not fully addressed in the scientific literature, there also exist examples of iridescent fungi, for instance, *Elaeomyxa cerifera*, where chitin plays a key role.

The best understood structures generating structural colors in the animal realm are based on chiral-nematic order, associated with Bouligand structures (Figure 5b2 and b3).^63^ Such color strategy enables selective polarization reflection, depending on the helicoidal turn taken by chitin nanofibrils, typically filtering light into left-handed polarized reflections. This is used as visual cues between animals that possess circularly polarized sensitive vision.^69^ When observing the helicoidal turns in Bouligand structures, the pitch that corresponds to half a full turn determines the reflected colors, with a larger pitch resulting in colors of larger wavelengths being reflected.^70^ In animals, reflections outside the visible range are commonly found. Structural colors are generally more pronounced in beetles due to their proportionally thicker cuticle.^71^

Beyond specific reflections, chitinous networks can form highly scattering layers (white), highly absorbing layers (black), or even high transmittance layers (transparent). Black structures are generally the result of a high concentration of cross-linked aromatics, typically melanins or pterins.^65^ An example of superwhite structures is reported for beetles, where assemblies (2 μm in thickness) efficiently reflect broadband color, *i.e*., white colors using inhomogeneous scattering from a perfectly isotropically, three-dimensionally ordered network of fibrils (Figure 5c).^72^ The multiscaled width and orientation of the bundles are critical to achieve high scattering over such a thick film. Angle-independent specific reflections can also be obtained by spherical features,^71,73^ although these are also structural colors, *i.e*., not involving pigments. Broadband reflections can also be induced by the introduction of a pitch gradient, where both mechanical and optical properties are matched.^73^ In the case of transparent structures, where reflections are minimized by conical features on the surface of the film, a quasi-monolithic layer constitutes the thin film to reduce the reflections. Therein, disorder of the conical structures enhances nonspecific reflections, contrasting directly with structural color generation.^36^

##### 2.1.1.3 Other Nanochitin-Based Functional Structures

**Figure 6:**
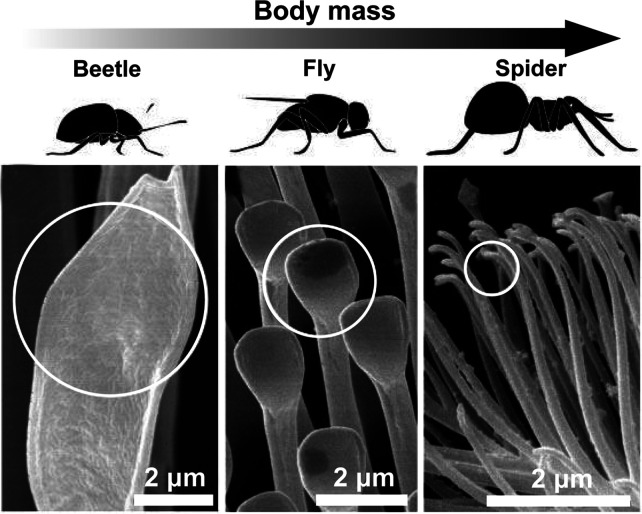
SEM images of the spatula- and spindlelike adhesive structures found in various insects (beetle, fly, and spider), indicating that heavier insect bodies exhibit finer adhesive structures. Adapted with permission from ref (76). Copyright 2003 National Academy of Sciences.

Setae and other pronglike appendages are intricate spatula- and spindlelike organizations found across the animal kingdom that control friction at interfaces, among other functions. Interestingly, these can be formed by chitin^33^ and other nonchitinous elements,^75^ typically found in lizards and insects. They are well studied in insects, since they promote a strong adhesion to a range of surfaces, including loose and hydrophobic surfaces. The relation between the structure and the density of adhesive setae is directly related to the body weight of the animal. For instance, spiders have smaller setae but at a higher areal density on their legs when compared to smaller insects, such as beetles, where setae are larger but in reduced numbers (Figure 6). Flies with intermediate sizes also bear intermediate spatula size and areal density.^76^ A gradient of mechanical stiffness, for instance, ranging from 1 MPa at the tip to 10 GPa at the base,^77^ is present along these appendages to enhance flexural properties and to match various surface topographies. Furthermore, the tip of the setae may be convoluted with microstructures, such as fingerprint-like structures, conical spikes, or corrugated ridges, to further increase the relative friction.^78^ In fact, adhesive structures can be found to scale continuously from such nanometric structures, toward millimetric claws, including intermediate micrometric setae arranged onto larger pads.^79^ Importantly, setae and other hair-type elements require optimal adhesive power since a too high adhesion leads to reduced cleaning potential, where dirt cannot be removed. This was demonstrated for bees, where a strong adhesion to pollen resulted in its permanent attachment.^80^ Chitin can also have a structural role, although rare, in excreted adhesives. This is the case of barnacle adhesives, which require a high resistance to mechanical stresses beyond a strong adhesion.^81^

Beyond color, adhesion, and structural mechanics, one should note that chitinous architectures have been considered in other roles.^82^ For instance, the antibacterial activity of chitin has been used in material science. Protection from pathogens is associated with the biological activity of chitin, for instance, for application in healthcare. Spindlelike chitinous structures can serve as vibration sensors or defense tool against predators.^82,83^ The latter urticating structures are typically observed in caterpillars,^84^ and longer extraction devices can be found in mosquitoes and butterflies, where the mechanical properties of theses constructs are optimized as a function of the target. Other examples include hydrophilicity and mostly superhydrophobicity at the interface,^35^ motion-generating structures,^85,86^ and vibrating structures used for sound generation.^87,88^

#### 2.1.2 Biogenesis of Chitin

**Figure 7:**
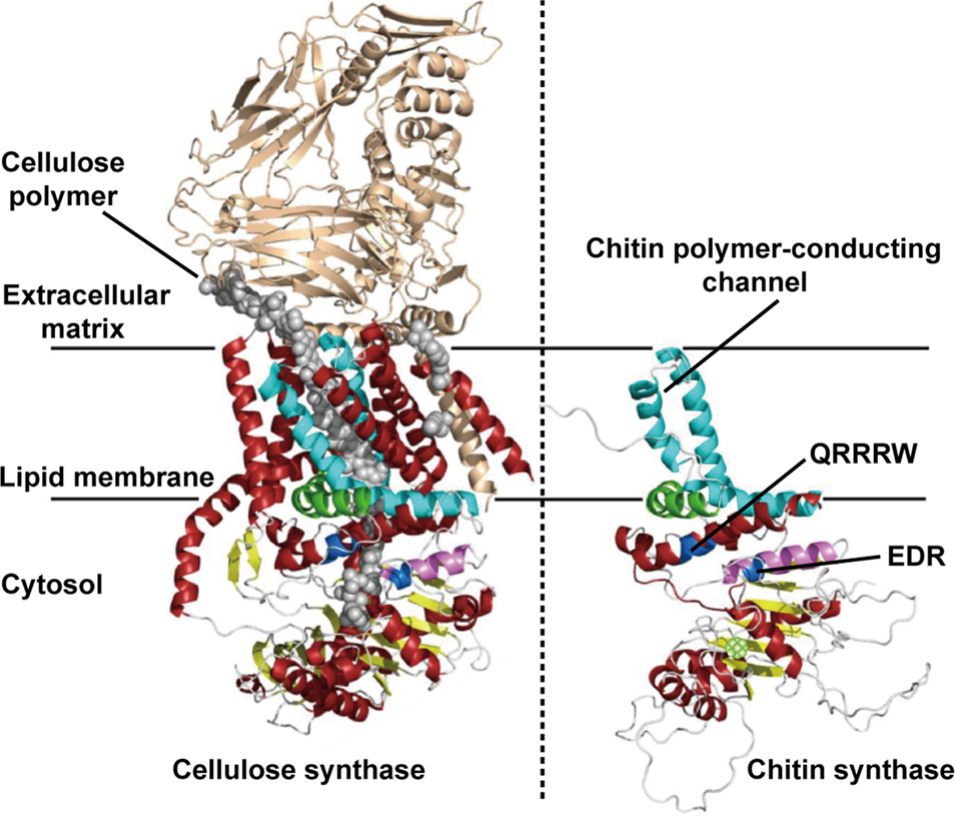
Three-dimensional schematic illustration of the structure of trans-membranous chitin synthase. The left panel is the crystal structure of the bacterial cellulose synthase complex from *Rhodobacter sphaeroides*. Here, the cellulose polymer is shown with gray spheres. The right panel corresponds to a computed 3D structure of the C-terminal parts of chitin synthase. The crystal structure of the bacterial cellulose synthase (left panel) was used as a template for structural predictions. The highly conserved amino acid sequences include QRRRW (product binding site) and EDR (saccharide binding site), found in other glycosyltransferases such as cellulose synthases. Adapted with permission from ref (94). Copyright 2017 MDPI.

We introduce the structure found within different organisms and the biosynthetic pathways leading to the formation of chitin. It is important to note that chitin synthesis is a highly complex process, where chitin is dynamically synthesized, degraded, bundled, and translocated and continuously forms superstructures. To date, many of these mechanisms are only partially known and put forward from indirect evidence.^89^ The synthesis of chitin is catalyzed by chitin synthases, glycosyltransferase enzymes, which are membrane complexes that polymerize *N*-acetylglucosamine into linear chains of β-(1,4)-acetylglucosamine.^90^ A three-dimensional structure can be predicted for chitin synthase, templated from its counterpart, namely, bacterial cellulose synthase (Figure 7). The synthesis takes place over three steps; *i.e*., the junction between monomeric units is made on the cytoplasmic side, followed by transmembrane transfer of the chain, and finally self-assembly of the polymers into fibers.^91^ The monomer used for synthesis is generally a phosphorylated precursor, *N*-acetylglucosamine-1-phosphate, that is appended the “UDP” functional group into the final precursor to chitin, UDP-*N*-acetylglucosamine.^92^ The general mechanism is the same for all glycosyltransferase, i.e., cellulose and hyaluronan.^93^ Therein, the enzyme exploits the difference in reactivity associated with the directionality of the reactivity of the growing chain due to the reducing end being more reactive.

##### 2.1.2.1 Structure of Chitin in Insects

Due to their facile collection, laboratory breeding techniques, and nonmineralized shells, insect chitins are the most well studied systems. However, despite over 100 years of studies dedicated to insect chitin biogenesis, many questions remain unanswered, including how the organization of chitin occurs. In insects, chitin synthesis takes place in the body wall or cuticle, in the gut lining or peritrophic matrix, in the salivary glands, trachea, and eggshells, and in the extremities of the muscles.^95^ Therein, both synthesis and degradation take place simultaneously and to different extents, which depends on the developmental stage. In insects, chitin contains a small fraction of glucosamine, making insect chitin an inherently heterogeneous polymer. The chitin fibrils within the gut and cuticle are organized into nanofibrils and sheets, which are themselves embedded into a protein matrix. At this stage, it seems more likely for the proteins to interact supramolecularly with chitin,^96^ although it is likely that phenol- or, more likely, catechol-amine reactions occur. Although polar in nature, the dehydration of chitins may be favored by interactions with hydrophobic proteins.^97^ In this so-called tanning process, hydrophobic proteins expel water and cross-link chitins *via* oxidation of the proteinaceous phenols into catechols, which can readily react with the amine group of chitin,^98^ this leads to very stiff insect parts.^99^ The process of cross-linking induced by dehydration is essential and is a key synergy between high-histidine content proteins and chitin. The soft insect cuticle contains up to 75% water, while the hard cuticle has a water content reduced to 15%. The phenolic-containing proteins that are oxidized to bear a high catechol content cross-link the proteins and chitins, tuning the mechanical properties of the constructs. The Young modulus of soft insect cuticles ranges from 1 kPa to 50 MPa, while that of tanned cuticles can reach up to 20 GPa. The presence of heavy metals, *e.g*., Zn, Mn, or Fe, further cross-links catechols.^100,101^ The elasticity is also modulated by resilin.^102,103^ Thereafter, chitin deacetylases play an important role in enhancing the interaction potential of the chitin fibrils with the extracellular matrix.^104^ Chitin microfibril synthesis occurs in the extracellular space, where proteins organize their three-dimensional layout. The main difference for the synthesis of chitin between organisms is the source of the glucose precursor. In the case of insects, it can even be body fat in the form of glycogen.

Insects periodically replace their outer shell as protein-based dehydration, *i.e*. tanning, rendering the shell too rigid for growth, which is achieved by secretion of the molten fluid containing chitinases and proteases. This process involves various steps that are crucial in various development stages or to answer to environmental stresses.^105^ The chitin content and obtainable yield of chitin fibrils is thereafter directly impacted by molting, wherein the chitin content is minimal right after molting. Chitin is hydrolyzed into oligomers by chitinase and β-*N*-acetylglucosaminidases that further degrade the oligomers into the monomer.^106,107^

In insects, chitin is highly associated with proteins throughout their bodies. The chitin–protein matrix defines entirely the stiffness of the insect’s shell by modulating interactions with water. Larval bodies and wings are highly flexible, while the heads and mandibles are the most rigid parts. Chitin binding proteins are either directly secreted into the extracellular matrix or stored in vesicles for later payload release. There is a wide range of proteins containing chitin binding motifs, which serve various functions, including structural consolidation, immunity enhancement, bactericidal effects, *etc.*(108) Many of the enzymes involved in the degradation of chitin possess such chitin binding domains.^109−112^ Another set of proteins, Knickkopf proteins, takes part in the organization of chitin within the body of insects, although the exact mechanisms involved in chitin translocation and bundle formation are unclear. This set of proteins is known to participate in the remodeling of chitin fibers since their absence in insects results in a lack of fibril alignment and lamination.^113,114^ Furthermore, there is a clear indication of fibrillar rotation during embryogenesis, suggesting an active formation of the structures.^115,116^ In the end structure, through post-tanning, chitin nanofibrils form bundles within the proteinaceous matrix of up to 100 nm, which are arranged at the macroscale.

**Figure 8:**
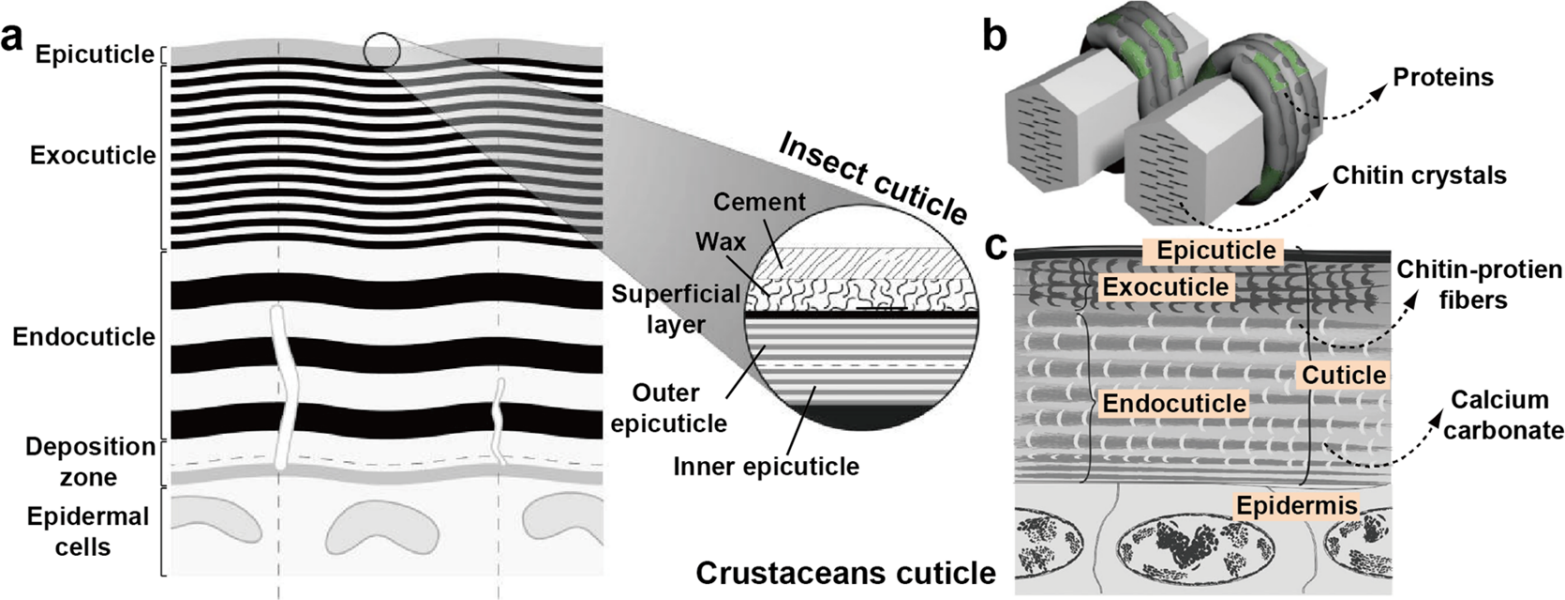
(a) Schematic illustration of a typical cross-sectional structure of an insect cuticle in the premolt stage, showing the most important layers, with a fully developed endo- and exo-epicuticle. (b) Model of complex protein–chitin superstructures via post tanning/sclerotization, wherein proteins (dark and green tubes wrapping around the chitin crystals) specifically interact with the multiple faces of chitin crystals as well as with the amorphous domains. Note: different colors represent different proteins. Adapted with permission from ref (74). Copyright 2008 John Wiley and Sons. (c) Simplified structure of crustacean cuticle, highlighting the mineralized domains within the cuticle, wherein the network of minerals is considerably more continuous in crustaceans than in seashells. Adapted with permission from ref (119). Copyright 2017 Elsevier.

The degree of crystallinity of chitin varies among different insects. However, the superstructure within arthropods generally presents a long-range order. For instance, a regular spacing of 7 nm and crystallites of 2 nm were reported in thin sections of a fly.^117^ This observation was further extended to chitin in arthropods.^118^ Chitin is then found across the cuticle of insects forming various structures. The cuticles contain the epidermal cells, where the chitin fibrils are synthesized, followed by the deposition zone where the initial chitin–protein biogenesis occurs. This is followed by the endocuticle, exocuticle, and epicuticle, where varying densities of chitin bundles and protein complexes are observed as a function of various tanning processes (Figure 8a).^74^ The epicuticle determines the hydrophobicity of the animal skin and is generally covered in wax and minerals. The epicuticle is typically 2 μm thick, while the exocuticle is about 1.5 orders of magnitude thicker, in the range of 100 μm. Epicuticle multilayers are generally thinner than those of the exocuticle. The basal chitinous layer, the endocuticle, presents a looser network of fibrils and a considerably lower extent of tanning.

##### 2.1.2.2 Structure of Chitin in Crustaceans

In crustaceans, chitin organization generally resembles that found in insect shells, forming microfibrillar bundles in strong and tough composites. The structure is usually mineralized with calcium carbonate structures. A range of structures is found in crustaceans wherein stiffness is mostly modulated by proteins, as is the case of insects. This is also the case of the squid beak, where histidine-rich proteins and α-chitin exist (ca. 2:1 ratio) in the absence of minerals.^37^ The mostly organic structure presents remarkable stiffness and hardness, making it possible to fracture the shells of gastropods. The highly cross-linked structure is resistant to proteolysis and is generally not digestible. Such cross-linking strategy is reminiscent of the tanning (sclerotization) process in insects, wherein a darkened color is observed as a result of aromatic cross-linking points (Figure 8b). The tanning process forms gradient-like structures, where the apex of the beak is the hardest while the base is hydrogel-like.

The crustacean cuticle possesses all of the layers found in the cuticle of insects,^54^ typically containing higher carotenoid content, which results in an orange appearance.^120^ Contrary to the case of insects, α-chitin structures in crustaceans mineralizes on the surface of the fibers into calcium carbonate crystals via calcification. As such, the chitin–protein complexes determine the cationic planes, which leads mineral plates. Initially, amorphous calcium carbonate precursors form highly ordered mesoscaled crystals,^121^ resulting from the highly crowded cellular environment.^122^ Their thickness can vary in the order of a few micrometers. Aragonite and calcite can be found in mollusk shells and sea urchins, respectively. Both amorphous calcium carbonate and calcite are found in crustaceans, with some amount of calcium phosphate.^123,124^ Mineralization occurs principally in the exocuticle and endocuticle (Figure 8c).^107^ The other polymorph, β-chitin, is commonly found in squid pens, making 31% dry matter equivalent, with a degree of acetylation (DA) of 96% and over 75% crystallinity.^125^ The structures formed by the fibrils are generally considerably softer than what is found in typical arthropod cuticles.

##### 2.1.2.3 Structure of Chitin in Other Organisms

**Figure 9:**
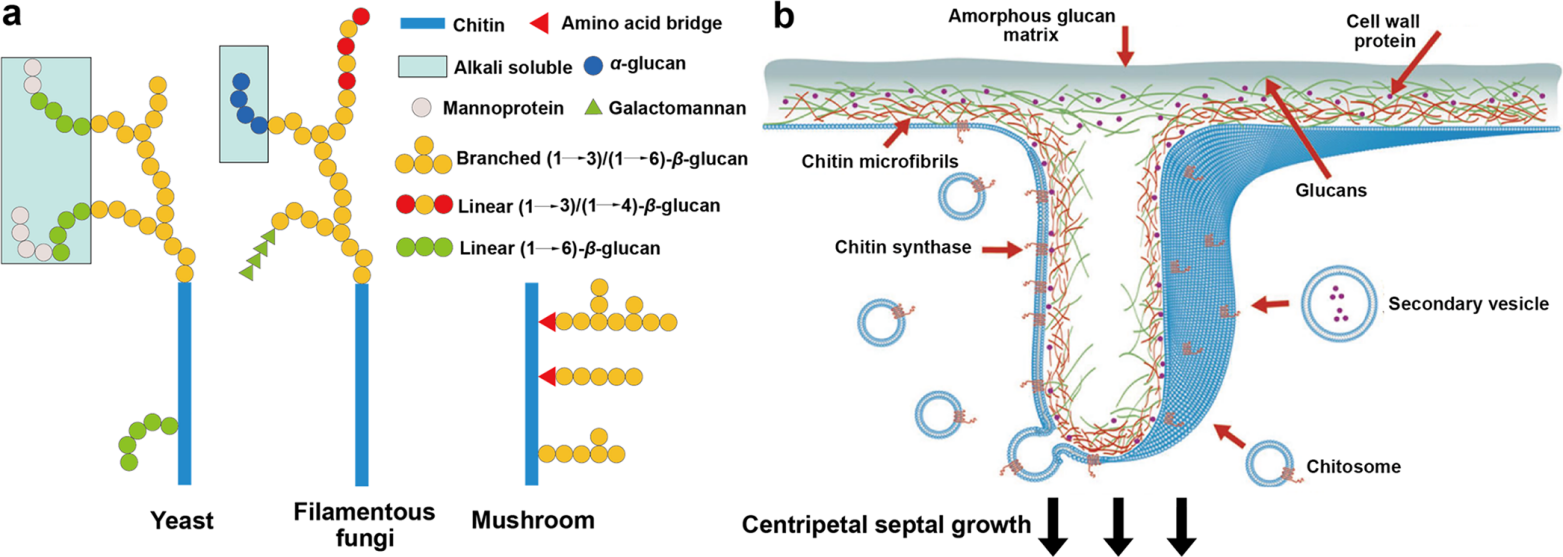
(a) Schematic illustration showing chitinous structures found in microorganisms. The chitin–glucan complex models include *Saccharomyces cerevisae* and *Candida albicans* for the yeast, *Aspergillus fumigatus* for the filamentous fungi, and *Schizophyllum commune* for the mushroom. Adapted from ref (135). Copyright 2020 American Chemical Society. (b) Three-dimensionally schematic illustration of the use of chitosome, a chitin-generating complex and its structure in fungi. Adapted with permission from ref (132). Copyright 2013 Elsevier.

In addition to arthropods, fungi contain chitin in the range of 5–27% as dry mass,^126^ which is principally found in the ring necessary for mitosis of the fungal cells^127^ and in the growing hyphal tip.^128^ The deacetylated form of chitin, chitosan, can also be found in fungi cell walls. Chitin is identified as a branched structure within a branched architecture composed of β-1,3 and β-1,6 glycans arranged within a highly hydrated amorphous matrix (Figure 9),^126^ located at the core of the branched polysaccharide superstructure or at its edges (Figure 9a).^129,130^ Chitin synthases in fungi are found principally within chitosome (Figure 9b), which are spherical lipid–protein assemblies with a diameter of 40–70 nm and a membrane of ca. 7 nm.^131,132^ They make the main constructs that deliver chitin synthase, a membranous protein, to the surface where chitin nanofibrils are assembled.^133^ It is believed that other chitin-forming entities also use chitosome-like structures for the translocation of chitin synthases. For instance, a high density of synthases was found at the apical region of microvilli in insects.^134^ As cellulosomes for cellulose, membrane enzymes are not soluble, producing polymers and fibrils through interfacial biogenesis. Microfibrils superstructured in triple helices can be found in the cell walls of mushrooms. They are composed of a range of glucans sparsely branched by chitin fibers. In the mycelium, the vegetative growth state of fungi filaments, a network with a high content of chitin (with other glucans), forms three-dimensional architecture of the growing organism.^135,136^ Nematodes and single cell organisms such as yeasts or protozoa can also form chitin,^137,138^ although their purpose may be more functional than structural since the chains remain relatively short and mediate hydrophilicity of the cell walls.^103^ Chitin in yeasts is also one of the elements that leads to mitosis of yeast cells.^139^

### 2.2 Chitin Chemistry

We next introduce chitin chemistry, from the molecular level, as in saccharide rings, to structural chemical organizations, as in raw chitin. Three aspects are considered: structural chemistry, surface chemistry, and bioactivity. In the first subsection, we discuss the various assemblies of chitosan into fibrils composed of given chitin polymorphs. We then introduce the surface chemistry of these fibrils, followed by a discussion about the impact of these fibrils in interfacing with microorganisms and complex living tissues.

#### 2.2.1 Chitin’s Structural Chemistry

**Figure 10:**
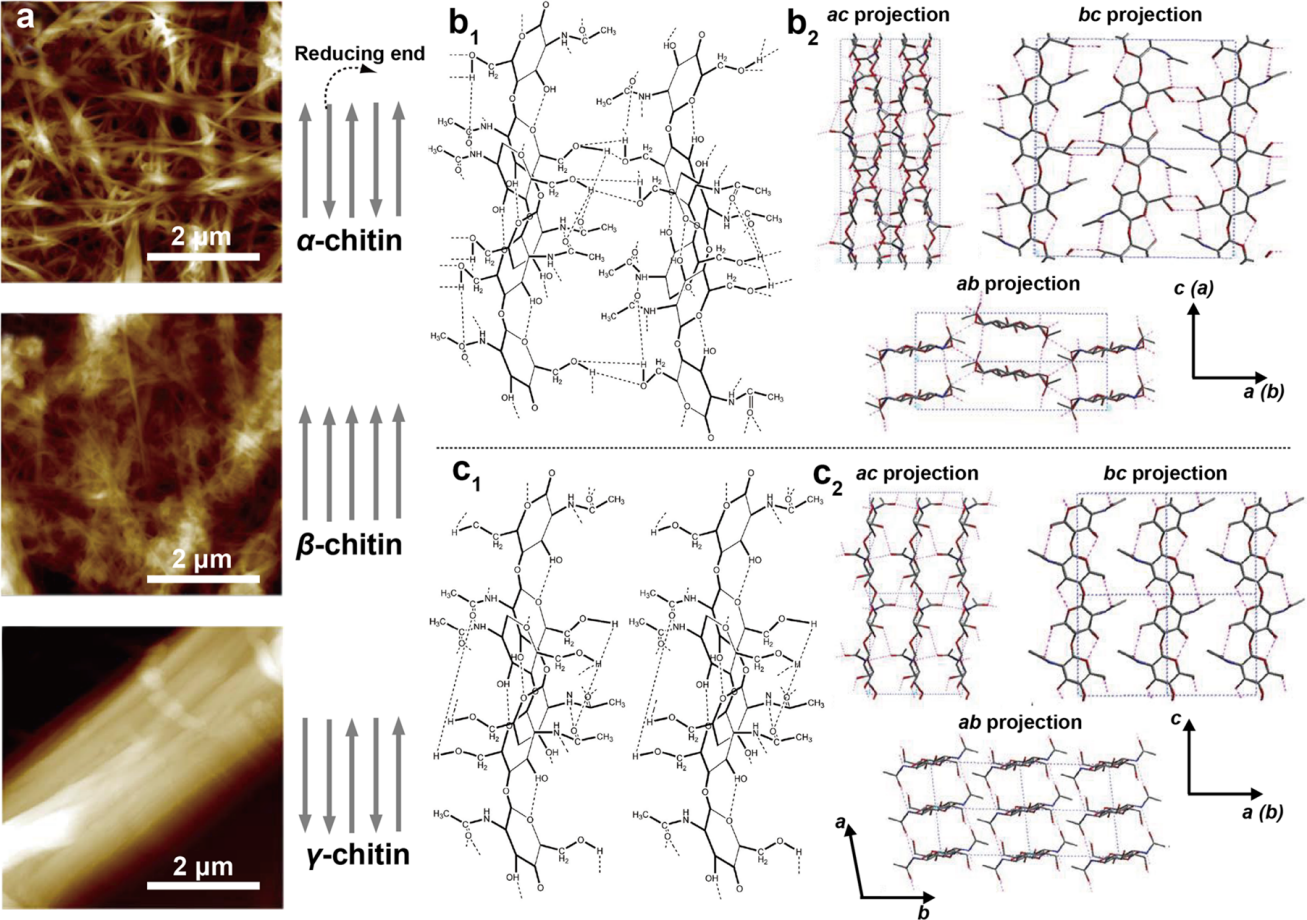
Illustration of three main polymorphs of chitin. (a) AFM images of three polymorphs of ChNFs obtained from the same acid/base extraction process. α-Chitin can be extracted from crab shells (*Potamon ibericum*), β-chitin from squid pens (*Sepia* sp.), and γ-chitin from moth cocoons (*Orgyia dubia*). Adapted with permission from ref (151). Copyright 2017 Elsevier. The gray arrows indicate the orientation of chitin macromolecules within the crystalline domains, with the arrows pointing away from the reducing end. Molecular structure and hydrogen H-bonding in (b1) α-chitin and (c1) β-chitin. Note: the intersheet bonding is absent in the case of β-chitin. Adapted with permission from ref (152). Copyright 2009 Elsevier. Structure of (b2) α-chitin and (c2) β-chitin in different projection planes (ac, bc, and ab). Adapted with permission from ref (9). Copyright 2006 Elsevier.

The molecular organization of chitin, as an abundant amino polysaccharide, involves rings in macromolecules and interactions with other elements, covalent or supramolecular, defining many of the functions in the living organism, within which they are synthesized. Nano- and microfibrils interact via H-bonding between amine and carbonyl groups. Small crystalline domains are embedded within pseudocrystalline and amorphous domains along the fibrils. The main crystalline allomorphs, namely α-, β-, and γ-chitin (Figure 10), are associated with a directionality with respect to its reducing end, which is a tautomer in equilibrium between the closed ring and open aldehyde forms. In the α-form, the chitin chains are arranged in an antiparallel fashion; on the contrary, the β-form has a parallel arrangement, while γ-chitin has two parallel chains neighboring one antiparallel one (Figure 10a).^90^ α-Chitin is the most abundant allomorph and is found in fungal and yeast cell walls as well as in arthropods, including crustaceans and insects. Compared with cellulose fibrils, chitin nanofibrils are generally considerably less polar but still highly hydrated in most animals, prior to tanning. Their hydrophobicity may be enhanced by biosynthesized hydrophobic proteins.^97^ β-Chitin has reduced H-bonding interactions compared to α-chitin and forms softer fibrils that are more susceptible to hydrolysis and overall swelling. α-Chitin is present in hard materials, while β- and γ-chitins are present in flexible structures.^140^

α-Chitin, an allomorph that is insoluble in most solvents, forms orthorhombic crystals, presenting both inter- and intrasheet bonding (Figure 10b). The theoretical axial elastic modulus of α-chitin is 150 GPa,^102^ with 59 GPa being the experimental value.^141^ The surface energy of different facets are affected by the crystalline allomorph. For instance, in the (010) face of α-chitin, the distance between available H-bonding groups matches that of a proteinaceous β-sheet such as silk.^142^ In fact, the (010) face is suggested to present the strongest interaction with proteins in general and proteinaceous residues are expected on this face after isolation of the fibers,^102,143^ which demonstrates that the optimization between the protein-chitin interface also exists in most chitin-containing animals.

β-Chitin, found as a hydrogel-like structure within the squid pen, sea tubeworms, and diatoms and most famously within the squid beak,^37,144−147^ is a monoclinic crystal that has no intersheet bonds (Figure 10c).^146^ β-Chitin is more susceptible to deacetylation by acid-based hydrolysis,^144^ and can be transformed into α-chitin when exposed to concentrated NaOH or HCl.^148,149^ Particularly, although β-chitin is insoluble in many solvents, it exhibits better solubility than α-chitin does; for instance, it is soluble in formic acid.^150^ γ-Chitin exists scarcely in nature and is found, for instance, in certain species of squid or beetles.^140,150^ α-Chitin and γ-chitin decompose at 330 and 310 °C, respectively, while β-chitin decomposes at a lower temperature, 230 °C. γ-Chitin was found to present microscaled fibers when extracted from the cocoon of the moth (*Orgyia dubia*), with extremely tightly bonded nanofibers as obtained from typical extraction processes (strong acid/base treatments).^151^ Accordingly, γ-chitin is digested at a slower rate compared with α- and β-chitin. Because chitin macromolecules are arranged in sheets and in β-chitin these sheets interact weakly, several polar molecules can penetrate β-chitin such as alcohols. In contrast, intersheet networks of α-chitin are tightly held by H-bonding.^9^

The solubility of chitin in aqueous media depends on the crystal allomorph and degree of surface acetylation.^152^ The highly deacetylated form of chitin, chitosan, forms highly viscous acid solutions and is generally insoluble above pH 6 due to its p*K*_a_ of 6.3.^153,154^ Meanwhile, chitin is insoluble in water at any pH condition. However, inorganic salts, such as Ca(CNS)_2_, CaI_2_, CaBr_2_, or CaCl_2_, have been found to facilitate chitin dissolution.^155^ The dissolution and regeneration of chitin result in materials with properties that are highly dependent on the solvents used.^156^ For example, lyotropic LCs form when chitin is dissolved in lithium chloride/dimethylacetamide at 2 wt %, where phase transitions are concentration-dependent, which can be used to enhance the long-range order of the polymers in the regenerated fibers.^157^ In addition to the lyotropic, a thermotropic behavior also exists, where phase transitions are temperature-dependent.^158,159^

#### 2.2.2 Chitin’s Surface Chemistry

The unique cationic character of chitin derives from the hydrolysis of surface acetyl groups, which dictates the properties of nanochitin. Here we discuss the chemistry of such surface groups and their distribution along the biosynthesized polymer, the interaction with the surrounding biological matrix, and the resulting superstructures upon extraction. The impact of other functional groups, *e.g.*, hydroxyls, other glucans, and residual proteins, is also discussed.

The most important aspect of chitin surface features is the presence of amine groups that result from a variable degree of deacetylation (DDA). Below pH 6, the ammonium ion bears a positive charge in aqueous media, which infers long-range electrostatic interactions prevailing over the short-range H-bonding interactions. At pH > 6, all of the hydroxyl, ether, acetyl, and amine groups contribute to H-bonding, forming a complex intracrystalline and interfacial H-bonding network. Furthermore, the ether and acetyl groups, considerably less polar, likely impart amphiphilicity. The number and distribution of amine groups are highly dependent on the source type and processing. For instance, yeast and, more commonly, fungi present a highly deacetylated β-chitin, in general more easily deacetylated due to the hydrated nature of the crystal. In contrast, chitin obtained from the endocuticle and exocuticle of arthropods is highly acetylated (>98%). Moreover, initial deacetylation is expected to occur more easily on the groups outside of the crystalline domains and in the amorphous regions; therein one group every two glucose units is first deacetylated.^160^ Thereafter, a broad range of deacetylation degree distribution is expected. Therefore, in typical chitin samples, a fraction is predominantly charged while another fraction may present rather low charges.^160^ Compared with cellulose, the presence of amine groups implies simpler modification routes, for instance, by using a wide range of functionalization approaches.^161^ Furthermore, amine groups, in addition to the primary hydroxyl groups, infer two functionalities for modification. Orthogonal chemistry can be used to exploit the considerably higher reactivity of the amine groups.^162^ There are numerous examples of chitin modification with hydrophobic groups, e.g., to reduce their hydrophilicity or improve their compatibility with other surfaces,^163^ which will be comprehensively discussed in the following subsection. Beyond surface energy, amine groups enable coordination bonds with heavy metals, making chitin a suitable option for water remediation and heavy metal separation.^164^

Commonly, surface chemistry of chitin extracted from arthropods is affected by proteinaceous residues that remain after extraction.^165^ In most arthropods, supramolecular and covalent interactions are extremely strong; for instance, interaction of chitin and protein is optimal when the protein forms β-sheet domains, wherein the spacing between the domains of the sheets matches that found between the monomeric saccharide units of chitin.^102,142,143^ Other chitin binding domains can be found on chitinases, also leading to strong supramolecular interactions.^166^ The surface chemistry of chitin extracted from glucan, in contrast to that extracted from arthropods, is highly contaminated with other glucans.^167,168^ The range of accessibility of glucan structures is rather wide.^135^ For instance, the water contact angle of films made from fungi chitin ranged from 24° to 66°, regardless of whether the fibrils were isolated from a mushroom stalk, cap, or paragus or from a mixture.^169^ The fungal source also significantly affects the surface charge; for instance, the zeta potential of nanofibrils ranges from −10 to −25 mV when extracted from *Agaricus bisporus* or *Daedaleopsis confragosa*.^170^

#### 2.2.3 Chitin’s Bioactivity

Chitin possesses principally a structural role in biological systems, but it also has immune-defensive and antibacterial activities. The bioactivity of chitin is mostly similar to and related to that of homologous chitosan, with additional implications of antifungal and antioxidant effects and uses reported in regenerative medicine and other applications.^130,171^

Chitosan has been extensively explored since its introduction in bactericide-related applications.^172−174^ The action of chitosan against microorganisms is reported to occur by several mechanisms, many of which are still unclear. Chitosan action as far as the disruption of the cell wall and subsequent microorganism lysis is assigned to its antibacterial activity, which is associated with charge matching with the anionic cell wall of the microorganisms.^175^ Gram-positive bacteria contain on the surface negatively charged teichoic acids, while Gram-negative bacteria are negatively charged given the presence of lipopolysaccharides.^176^ The disruption of the membrane is found to increase by quaternization of the charged groups of chitosan,^177^ which also affects certain fungi.^178^ Another mechanism is associated with small chitosan and endosomal escape, wherein interactions with messenger ribonucleic acid in the targeted microorganisms disrupt the synthesis of key proteins.^179^ This mechanism is also responsible for the lysis of yeasts and fungal cells by chitosan. Chitosan as an adjuvant increases the potency and control internalization/release mechanisms of active compounds affecting mammalian cells.^180^ The chelation potential of chitosan at neutral pH and above is another route that leads to the disruption of the metabolic activity of microorganisms.^181^ This is because both Gram-positive and Gram-negative bacteria require divalent ions for proliferation. High-molecular-weight chitosan also impacts the proliferation of bacteria by adsorbing and crowding on the surface, preventing the uptake of key nutrients.^181^ Overall, the antibacterial and antifungal activity of chitosan is well documented and used to prevent infection in a range of biomedical and agro-industrial applications, for instance, to protect plants such as tomatoes and cucumbers as well as to prevent dental and respiratory infections.^182^

Chitosan has been demonstrated to be nontoxic to a range of mammalian cell lines.^183^ In wound treatment applications, it stimulates the immune system, accelerating the onset of the inflammatory phase and thus the overall healing process.^184^ The potential of chitosan in regenerative medicine is associated with its similarity to glycosaminoglycans, which are the main components of the extracellular matrix guiding injury regeneration.^185^ During the inflammatory response, chitosan has been shown to interact with immune cells, such as macrophages and neutrophils.^186^ In turn, chitosan enhances fibroblast proliferation,^187^ which relates to the interactions with anionic biomacromolecules, such as heparin or proteins recruited on site.^188^ Chitosan is documented to form complexes that induce flocculation, most relevant in water treatment, but it is also generally recognized as safe by the U.S. Food and Drug Administration.^189^ Thus, it is extensively used in the food and beverage industry for its antimicrobial and antioxidant activity.^190,191^ When applied to oysters (for raw consumption), it suppresses bacterial growth and extends the shelf life.^192,193^

Mirroring the biological activities of chitosan, it is reasonable to expect that chitin should exhibit similar functions.^194,195^ However, since many of the bioactivities of chitosan are associated with chitosan’s cationic surface charges, the biological activity of chitin is considerably lower. Chitin is known to decrease the proliferation of fibroblasts, while highly deacetylated chitosan (>80%) significantly increases the proliferation.^187^ Besides the biological activity of chitin,^17,196−198^ rapid erythrocyte aggregation and fast subsequent clotting has been shown to be induced by chitin.^199^ It can be biomineralized with hydroxyapatite for tissue regeneration applications.^200,201^ Moreover, chitin fibers have been proposed for sutures due to their resorbability;^202^ meanwhile, chitin mats showed potential use as temporary skin for open wounds.^203^ Generally, the antimicrobial activity of chitin is associated with its molecular weight, and, as expected, with DDA.^204^ A higher DDA results in increased biocompatibility, antimicrobial, antioxidant, and hemostatic activity as well as mucoadhesion.^171^ Such effects depend on the chitin source; for instance, the antimicrobial activity of fungal chitin is higher compared to that derived from crab.^205^ This is related to the presence of residual proteins in the latter and the higher DDA of the former. Strategies to enhance biological functions use knowledge about the structural chemistry of chitin and upcoming explorations involve the use of nanochitin and methods to control its surface functions (charges, chemical groups, etc.)

## 3 Isolation and Engineering of Nanochitin

The insolubility of chitin in water challenges processing,^12^ particularly for the direct production of chitin nanomaterials.^206^ The preparation of nanochitin has greatly advanced in recent decades given the knowledge gained about chitin and its assembly in biological materials. The routes used in nanochitin isolation include top-down and bottom-up approaches, e.g., direct isolation of chitin nanoparticles from the biological materials and assembly of chitin molecules into nanofibrils, respectively.^18,207^ Accordingly, we emphasize the top-down approaches owing to the structural benefits of nanochitin encoded in the living materials. The most recent progress in nanochitin isolation and chemistry is presented next.

### 3.1 Chitin Extraction

**Figure 11:**
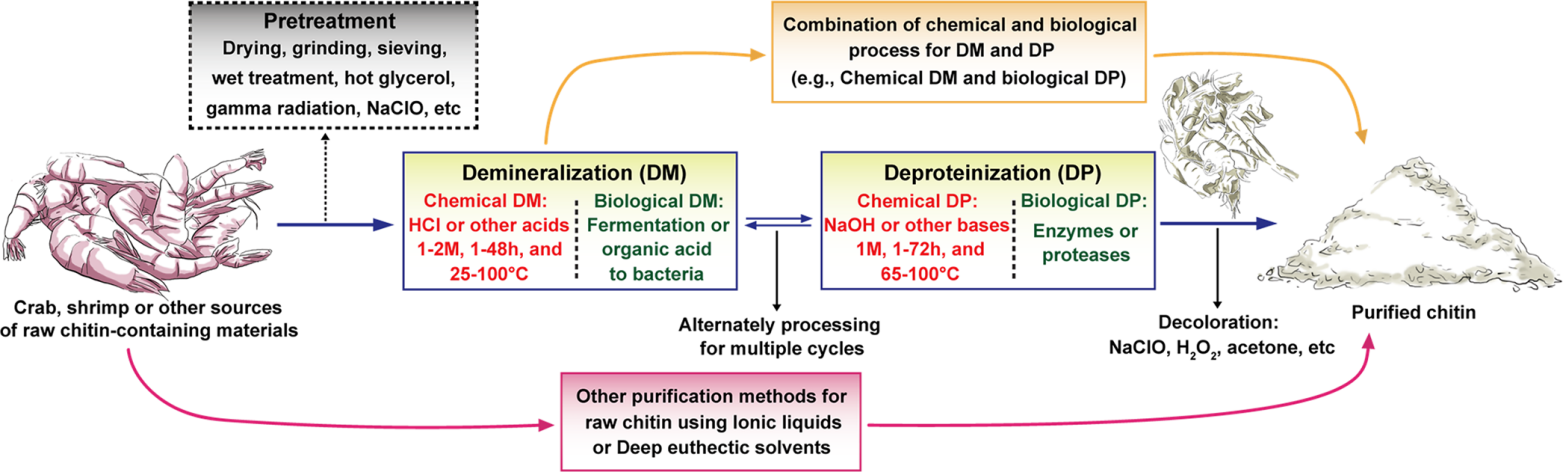
Overview of chitin extraction from crustacean shells that involves demineralization (DM), deproteinization (DP), and discoloration (all shown in solid boxes) as well as alternative pretreatments (dashed box). New extraction methods are emerging given considerations of environmental impact and sustainability.

In principle, elementary chitin nanofibrils are wrapped by proteins forming chitin–protein complex superstructures, embedded in minerals (crystalline calcium carbonate and a small amount of calcium phosphate) when forming the hard stratum corneum of crustacean shells.^208^ Thus, the procedure for nanochitin isolation from crustacean shells usually starts with chitin extraction (purification), demineralization (DM), deproteinization (DP), and discoloration (Figure 11).

DM and DP are used to remove calcium minerals and proteins complexed with chitin, respectively, which frees tightly bound chitin and might require multiple cycles, depending on the chitin source and intended uses.^209^ The discoloration, e.g., using sodium hypochlorite, acetone, or hydrogen peroxide, removes pigments adhered on the shells, e.g., astaxanthin and β-carotene, so that colorless chitin is generated prior to nanofibrillation.^16,210^ Apart from these procedures, pretreatments involving size reduction, drying, grinding, sieving, and wet processing of crustacean shells are applied before DM and DP, which facilitate the extraction and increase the efficiency of the process.^211,212^ For instance, a pretreatment using hot glycerol, a plasticizing agent, improved the efficiency of chitin extraction from prawn shells,^213^ and utilization of 3% NaClO before DM and DP reduced the processing time and energy consumption.^214^ There are reports on the use of atmospheric pressure dielectric barrier discharge plasma or gamma irradiation to increase the removal of minerals and proteins.^215,216^ Although other treatments might facilitate chitin extraction, impurities or uncontrolled degradation may occur, which would affect the properties of chitin and the obtained nanochitin.

A typical method for chitin extraction involves acids and bases for DM and DP, respectively, where DM implies the use of acid–base reactions to release water-soluble calcium salts, carbon dioxide, and water. Meanwhile, the DP process dissolves proteins adhered to chitin by using alkaline solutions.^217^ As a consequence, the extraction conditions are important since they determine the purity and type of chitin, as well as the total cost.^165,218−221^ In the case of DM, HCl is the most commonly used acid, for instance, at 0.3–2 M concentration, 1–48 h, and 25–100 °C.^165^ Other acids, such as HNO_3_, H_2_SO_3_, or organic acids including CH_3_COOH, HCOOH, C_6_H_8_O_7_, and their combinations, have been used to remove minerals from the shells.^222^ For DP, a typical procedure is to treat the shells with NaOH, for instance, 1 M concentration, 1–72 h, and 25–100 °C.^165^ Similarly, different types of alkaline reagents, e.g., Na_2_CO_3_, NaHCO_3_, KOH, K_2_CO_3_, Ca(OH)_2_, Na_2_SO_3_, NaHSO_3_, CaHSO_3_, Na_3_PO_4_, and Na_2_S, have been successfully applied.^209^ Although chemical extraction of chitin from different crustaceans is well established, recent studies focus on optimizing the conditions to minimize the impact on chitin’s molecular weight, to improve the yield and to decrease the processing cost. Microwave radiation has been used as a heating mechanism for chitin extraction, leading to reduced treatment time;^223,224^ likewise, sonication has been shown to improve the efficiency of chemical DP to produce chitin from shrimp.^225^

Chemical extraction has some disadvantages, such as environmental and sustainable impacts; for instance, the solubilized minerals and proteins are side streams that are not recovered or used.^210^ Thus, efforts in recent years have been directed to develop environmentally friendly chemical methods,^226^ and have included considerations to biological routes (Figure 11). An emerging area is that of chitin biorefineries, wherein all the byproducts generated, including proteins and pigments, are reused.^227^ For instance, DM that used citric acid combined with biological methods (pancreatic enzymes) at relatively low temperature (40 °C) have achieved good extraction yields.^213^ The biological treatments can access microorganisms that ferment the waste from DM,^228,229^ and the use of enzymes, including proteases, has been reported for DP.^209^ The fermentation of chitin shells using organic acids, either in the presence or absence of lactic acid bacteria, allowed efficient removal of minerals upon extraction.^230,231^ In addition, such processes can be extended to dual or multiple stages that involve fermentation, cofermentation, postfermentation, or autofermentation from endogenous microorganisms.^16,232^ On the other hand, treatment of chitin shell residues with proteases enables the replacement of chemical DP, for instance, using proteolytic enzymes such as chymotrypsin, papain, pancreatin, and others.^233^ Despite the environmental advantages of biological processes for chitin extraction, they usually require long processing times and are more expensive. Meanwhile, they present relatively low yields and so far are mostly limited to laboratory scales.^210^ However, a comparison of chemical and biological treatments for chitin extraction from crustacean shells on a pilot scale, based on sustainability parameters, underscored the great promise of biological processes.^234^ Recently, ionic liquids and deep eutectic solvents have been considered for chitin extraction from shells.^235−237^ For instance, 1-ethyl-3-methylimidazolium acetate was applied to process shrimp, wherein chitin in the shells was first solubilized and then precipitated as purified solid for further processing.^238^ Deep eutectic solvents are considered for fast, easy, and ecofriendly chitin extraction and typically involve a mixture containing choline chloride with an active ingredient, e.g., thiourea, urea, glycerol, and organic acid.^239−241^ However, similar to biological treatments, practical use has been limited by considerations of supply, scale, and cost.

### 3.2 Isolation of Nanochitin

**Figure 12:**
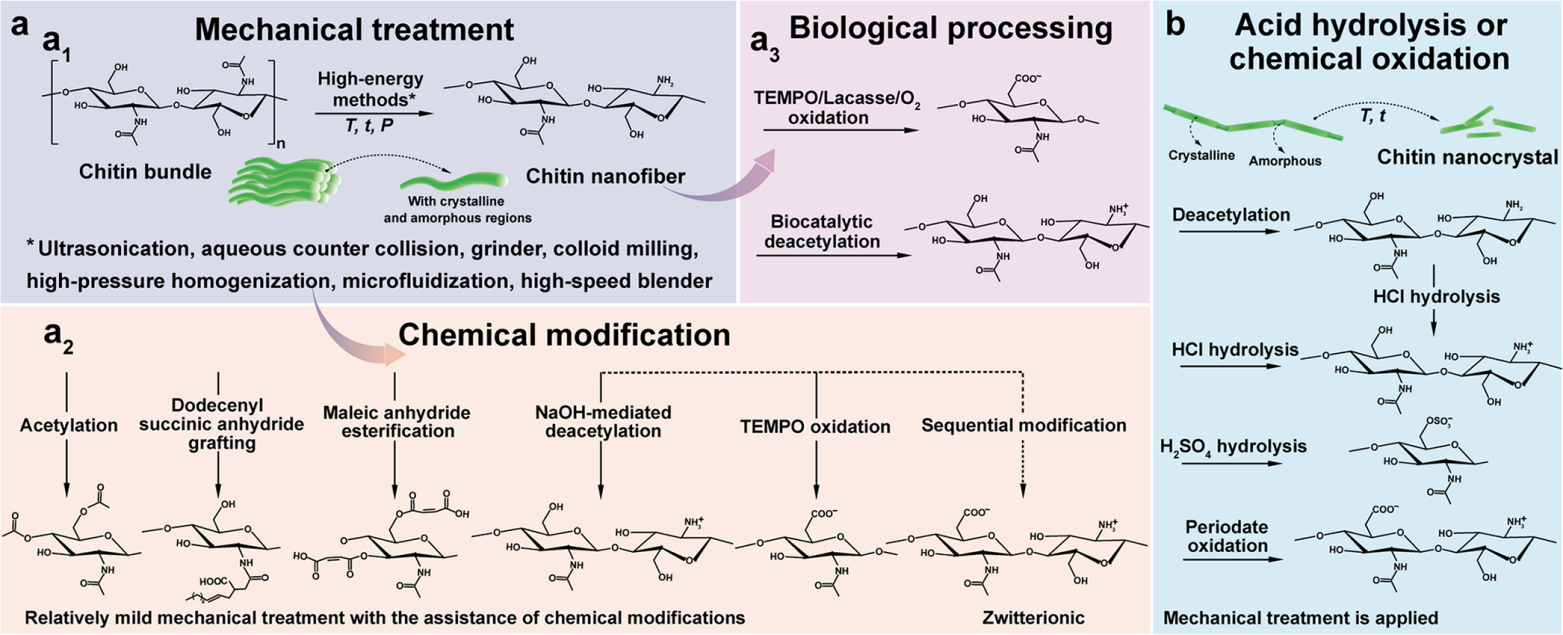
Overview of the methods used for isolation of (a) ChNF and (b) ChNC. ChNF containing crystalline and disordered structures is produced by (a1) mechanical treatment or mechanical treatment assisted by (a2) chemical modification and (a3) biological processing. The main goal of the chemical and biological modifications is to endow additional chemical features that facilitate mechanical fibrillation. ChNC is produced by surface exfoliation and by removal of disordered chitin structures using strong chemical processing with acids or oxidizing agents.

There is a great interest in the isolation of fibrillar chitin because the structural, chemical, and biological advantages can be gained from their morphological and nanoscale aspects.^242^ To this end, the top-down approach is widely used due to the possibility of maintaining chitin’s semicrystalline structures, 1D nanofibrous morphology, and thereby intrinsic performance.^243^ Typically, two types of nanochitin, chitin nanofibers (ChNF) and chitin nanocrystal (ChNC), can be isolated, Figure 12. The properties of nanochitin depend on the source of chitin, its isolation conditions and modification strategies, as well as the specific requirements for the desired applications. The mechanism for isolating ChNF considers the mechanical fibrillation of the chitin bundles, leading to nanoscaled lateral sizes with enhanced interfibrillar repulsion, most often generated by the nanofibrils’ ionized surfaces that retain both the amorphous and crystalline regions (Figure 12a). By contrast, the preparation of ChNC involves chemicals that exfoliate the surface of the chitin nanofibrils and remove the disordered chitin structures to yield ordered, crystalline ones (Figure 12b). Thus, the isolation of ChNF utilizes mechanical nanofibrillation with the assistance of chemical or biological processing,^244^ while strong acid hydrolysis or oxidation is used for ChNC production.

#### 3.2.1 Isolation of Chitin Nanofiber, ChNF

##### 3.2.1.1 Mechanical Treatment

**Figure 13:**
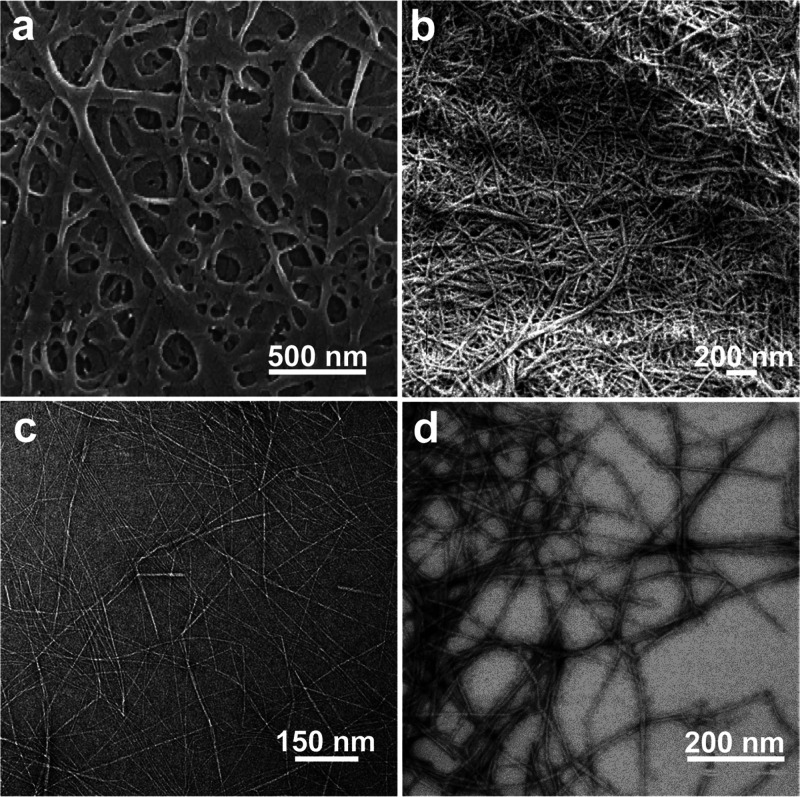
Microscopic images of ChNF prepared by typical mechanical nanofibrillation. SEM images of (a) ChNF isolated from chitin via ultrasonication in water and (b) ChNF isolated from crab shell α-chitin after one-pass grinding in acetic acid medium. Adapted with permission from ref (247). Copyright 2007 AIP Publishing LLC. Adapted from ref (250). Copyright 2009 American Chemical Society. TEM micrographs of (c) ChNF produced from squid pen β-chitin by one-pass microfluidization in acetic acid medium and (d) low-protein ChNF produced from lobster exoskeletons by microfluidization. Adapted with permission from ref (258). Copyright 2019 The Royal Society of Chemistry. Adapted with permission from ref (259). Copyright 2014 Elsevier.

In principle, ChNF isolated by mechanical nanofibrillation yield long, fibril-like morphologies (submicrometers to micrometers) with widths of the order of a few nanometers up to tens of nanometers, leading to structures of high aspect ratio, which is dependent on the chitin source and the choice of the mechanical treatment used. Representative mechanical methods that have been reported for nanofibrillation of chitin into ChNF are listed in Figure 12a. Ultrasonication is a simple high-energy method to disassemble natural chitin fibers into ChNF.^245,246^ For instance, ultrasonication at 20 kHz, 900–1000W, and 30 min in water at neutral pH was used to fibrillate chitin into nanofibers (widths of 25–120 nm) (Figure 13a).^247^ Acoustic cavitation at high frequency caused the formation, growth, and collapse of microbubbles in aqueous media to provoke microjets and shock waves on the surface of the chitin fibers, which etched them and promoted their disintegration along the axial direction. The ultrasonication process has also been applied in acid media.^248^ At pH 3–4, a transparent (85% for visible light) and viscous suspension containing individualized ChNF (length of several micronmeters and width of 3–4 nm) was obtained by ultrasonicating squid pen β-chitin (19.5 kHz, 300 W and relatively short duration). In this procedure, a thin ChNF was produced under short processing times with low energy input compared with that prepared by ultrasonication at pH 7 in water. Moreover, the obtained ChNF presented a DA of 0.9, indicating the presence of amino groups. Thus, protonation of amino groups on the crystalline surface of β-chitin in acidic medium enabled cationic surfaces. Together with the weak intermolecular forces in β-chitin, efficient nanofibrillation took place due to the electrostatic repulsive forces generated between the nanofibers in the β-chitin bundles. Such conditions are not effective in the case of crab α-chitin given its high DA, strong intermolecular forces, high crystallinity, and different packing arrangement of chains.

Grinding is another mechanical method that disintegrates chitin into ChNF,^249^ wherein the breakage of chitin bundles to thin nanofibers is caused by the continuous shear generated between grinding stones operated under a small gauge. ChNF was prepared by passing a never-dried crab chitin through a grinder (pH 3, 1500 rpm and 0.15 mm milling gap), which resulted in a gel-like suspension containing long nanofibers with 10–20 nm in width (Figure 13b).^250^ Meanwhile, dried chitin powder from crab shells was adequately fibrillated via grinding under acidic conditions, leading to ChNF (10–20 nm width) of high aspect ratio, similar to that obtained from never-dried chitin.^251^ In these cases, although the DDA of native chitin was relatively small, the electrostatic repulsion generated by the amino groups on the chitin in acidic medium was sufficient to break the strong H-bonding between nanofibers in the bundles. Besides acidic conditions, ChNF with a width of 10–20 nm and uniform shape was directly prepared from prawn shells using 1500 rpm grinding in water at neutral pH.^252^ The reason that nanofibrillation of chitin from prawn shells effectively occurs in neutral media is a consequence of the prawn exoskeleton, which is made up of exocuticle, a structure that is finer than that of crab shells, which are made up of 90% endocuticle, thus allowing easier disintegration, even under weak electrostatic repulsion.

High-pressure homogenization has been widely used to disintegrate bulk materials, which is suitable to produce ChNF from chitin.^253,254^ For example, dynamic high-pressure homogenization was utilized to isolate ChNF from lobster residues following 40 passes at 1000 bar.^255^ Through this process, nanofibers (width in the range of 80–100 nm) of high aspect ratio were obtained. Particularly, homogenization is a complete mechanical process that does not require the addition of acids or other chemical treatments, thereby causing little change to the chemical or crystalline structure of chitin. However, owing to the intense nanofibrillation, it is still interesting to understand how the surface properties of chitin change during processing. For this purpose, ChNF was prepared from purified crab shell chitin by repeated high-pressure homogenization (200 MPa with 10 passes) in water. Such a process yielded a heterogeneous network of nanochitin with widths ranging from a few nanometers to several tens of nanometers.^256^ Solid-state ^13^C NMR spectra revealed that the chemical shifts of all carbons remained unchanged before and after nanofibrillation and all C6-OH groups had the gauche–gauche conformation. Nevertheless, the weight-average molar mass of ChNF was only approximately 60% that of the original chitin, and the DA of ChNF increased from 0.83 to 0.98, indicating that the C2-NH_2_ groups presented in original chitin were partially removed during high-pressure homogenization in water. Thus, high-pressure homogenization alters the surface properties of the as-prepared ChNF.

Microfluidization has been developed as a high-energy method to produce ChNF.^257^ Interestingly, squid pen β-chitin was easily fibrillated into ChNF (1–3 μm in length and 2–7 nm in width) by using a one-pass microfluidization in acetic acid (Figure 13c).^258^ In addition, the fibers had a DA of 99%, indicating the importance of the amino groups to facilitate nanofibrillation of the chitin bundles. As noted earlier, isolation of nanochitin requires the removal of minerals and proteins; however, microfluidization is capable of producing protein-containing ChNF from mineral-free chitin. For instance, individual ChNF exhibiting nanofiber width of 3–4 nm and low protein content was successfully isolated from lobster exoskeletons by using microfluidization (5 passes, 900 and 1600 bar, Figure 13d).^259^ Apart from the single homogenization approach, mechanical defibrillation involving several steps was applied to fibrillate chitin into ChNF without changing the chemical or structural features.^260^ In this procedure, a stage-wise process, including 10-pass grinding (0.2 mm milling gap), 10-pass microfluidization (30 000 psi, 120 mL/min), and homogenization, was developed, resulting in nanofibers with a length of greater than 1 μm and width of approximately 50 nm.

Other high-energy methods, using a high-speed blender or a star burst system,^261,262^ have been shown effective to produce ChNF from native chitin. For instance, an aqueous counter collision technique was successfully used to prepare ChNF (width in the range of 10–20 nm).^263^ This process involved wet pulverization of purified chitin and ejection of a liquid dispersions from a pair of nozzles operated at high pressure, as a pair of jets that collide against each other. In this treatment, the interfacial interactions within chitin chains were cleaved solely by the high pressure, with no need for chemical modification. As the number of passes increased, more nanofibers (with smaller width) were dispersed in water. The obtained ChNF showed a favorable network formation in suspension, likely a result of the less charged nature of the ChNFs and their relatively large size.

Mechanical nanofibrillation methods are simple and adaptable, and they yield ChNF with high aspect ratio and with no need for any chemical reaction while keeping most of the original features of the native chitin. Some factors should be considered when choosing the mechanical method. As far as obtaining a higher degree of fibrillation, one should consider: (1) the source of chitin (high DDA, low crystallinity, weak intermolecular interaction forces, and fine exocuticle structures), e.g., squid pens and prawns facilitate production; (2) the apparatus (high energy output, more intense local impact, and multiple processing cycles during disintegration); and (3) the environment (acidic pH condition during processing facilitates nanofibrillation).^264^ The main drawbacks of mechanical nanofibrillation include the availability of special instruments, relatively high energy consumption, and production cost as well as possible incomplete individualization of ChNF.

##### 3.2.1.2 Mechanical Treatment Assisted with Chemical Modification

**Figure 14:**
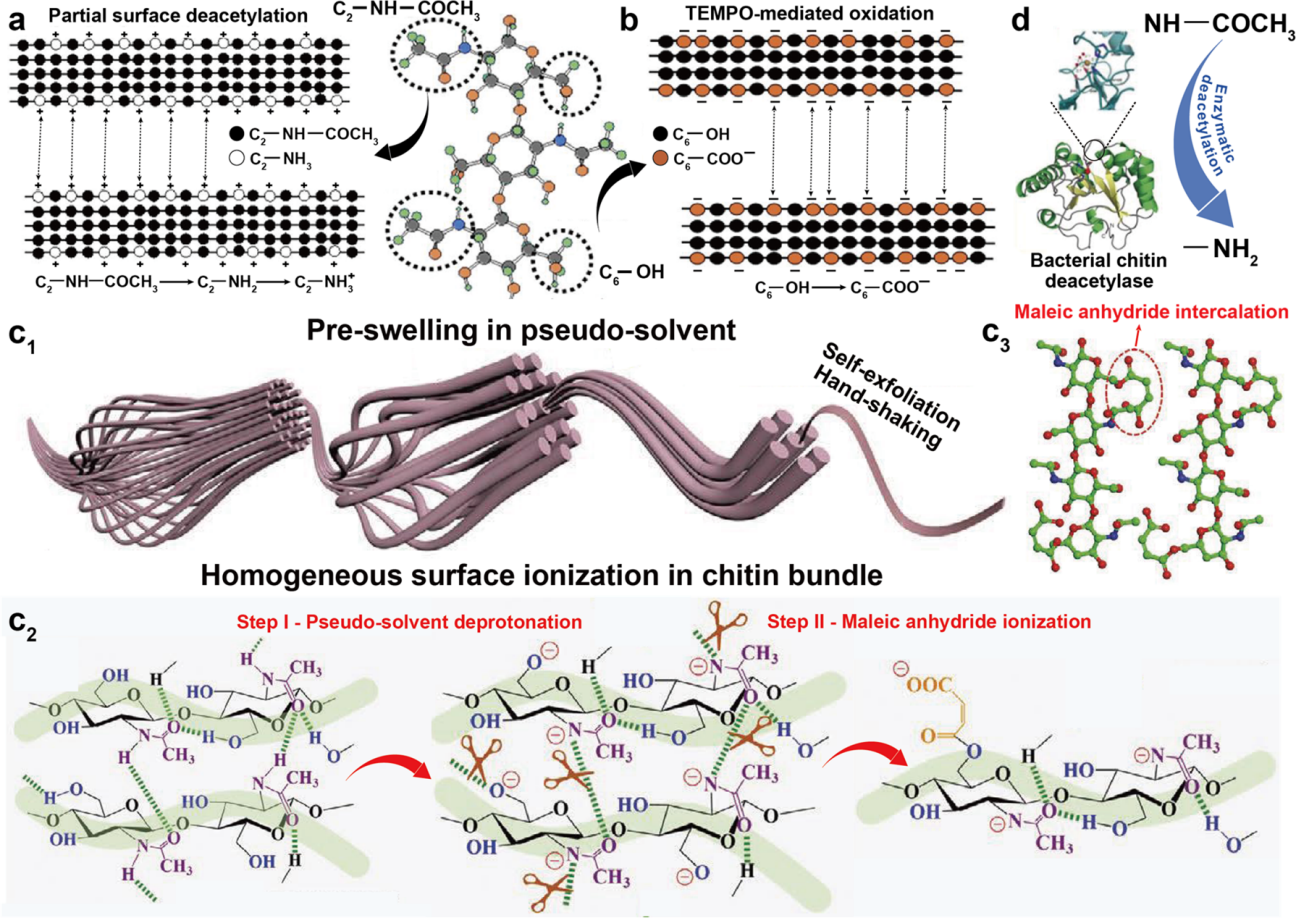
Schematic illustration of the reaction mechanism that takes place at the molecular level following (a) partial deacetylation and (b) TEMPO-mediated oxidation on the surface of chitin prior to fibrillation. Adapted with permission from ref (19). Copyright 2018 Elsevier. The molecular structure of chitin is included to indicate the reaction sites in the different processing steps. (c) Self-exfoliation pathway and mechanism of native chitin assisted by the pseudosolvent treatment. Adapted with permission from ref (288). Copyright 2021 John Wiley and Sons. (c1) Schematic illustration of self-exfoliation of chitin into ChNF using pseudo-solvent swelling and subsequent homogeneous surface ionization. (c2) Two-step sequential chemical and structural evolution of chitin during the self-exfoliation process, wherein step I involves deprotonation of chitin in and step II corresponds to ionization of chitin with maleic anhydride. (c3) Model showing of pseudo-solvent-assisted maleic anhydride intercalation into chitin molecules. (d) Enzymatic deacetylation of chitin surface via bacterial chitin deacetylase. Adapted with permission from ref (290). Copyright 2019 The Royal Society of Chemistry.

To function as a building block, the properties of ChNF should be uniform, reproducible, and adjustable. Thus, the use of mechanical treatment alone makes it difficult to satisfy all of these criteria. To improve the efficiency of ChNF isolation and endow additional functionality, chemical modifications are often performed on native chitin before mechanical treatments.^265^ For instance, oxalic acid can be used to hydrolyze shrimp α-chitin, leading to the installation of carboxylic groups on the surface of the fibrils, which results in a high mechanical disintegration (ultrasonication) efficiency in mild conditions.^266^ The crystallinity and thermal stability of α-chitin were basically unaltered under mild treatment, while the obtained ChNF was homogeneously dispersed in water at a wide range of pH (3–11). This result demonstrates the benefit of chemical modification in the mechanical treatment of chitin. Surface ionization, such as deacetylation, oxidation, esterification, and etherification, are commonly applied to deconstruct natural materials into nanofibrils.^267,268^ Among these strategies, partial deacetylation (Figure 14a) and chemical-mediated oxidation (mainly 2,2,6,6-tetramethylpiperidine-1-oxyl radical, TEMPO) (Figure 14b) are the two primary methods used to enhance chitin nanofibrillation during mechanical treatments, e.g., by endowing charged groups on the surfaces of native chitin that generate electrostatic repulsive forces,^269,270^ eventually resulting in fine ChNF.

**Figure 15:**
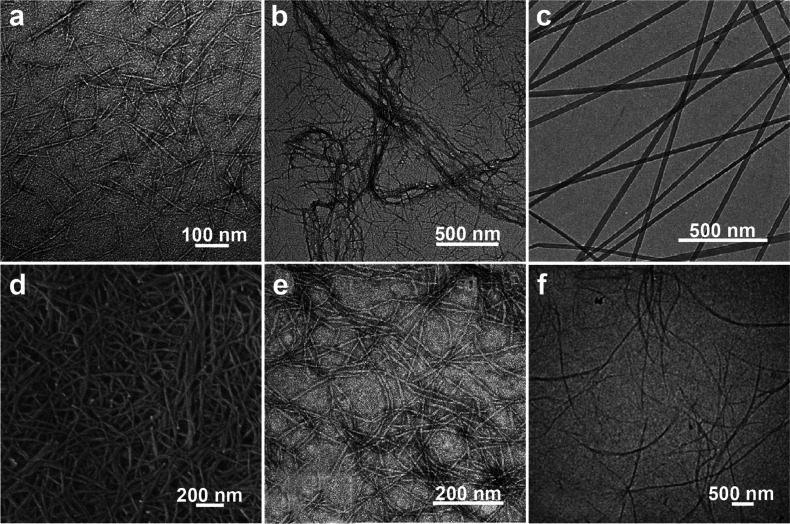
Images of ChNF prepared by mechanical nanofibrillation assisted by chemical treatment. TEM images of ChNF isolated by (a) ultrasonication and (b) microfluidization of partially deacetylated α-chitin from crab shells in acidic water (acetic acid). Adapted with permission from ref (272). Copyright 2010 Elsevier. Adapted from ref (273). Copyright 2019 American Chemical Society. (c) TEM image of ChNF produced from TEMPO-oxidized tubeworm β-chitin in water. Adapted with permission from ref (279). Copyright 2009 Elsevier. (d) SEM image of ChNF isolated via grinding of maleic anhydride-esterified α-chitin in water. Adapted with permission from ref (283). Copyright 2016 Elsevier. (e) TEM micrograph of ChNF self-exfoliated from squid pen β-chitin by pseudo-solvent swelling and maleic anhydride intercalation. Adapted with permission from ref (288). Copyright 2021 John Wiley and Sons. (f) TEM image of ChNF mechanically disintegrated from chitin deacetylase-processed α-chitin in acidic water. Adapted with permission from ref (290). Copyright 2019 The Royal Society of Chemistry.

In the case of partial deacetylation, the alkaline-mediated reaction occurs with the acetyl groups of acetamide at the C2 position on the surfaces of chitin crystallites, from which the acetyl groups are partially removed and leads to the exposure of C2 amino groups.^19,271^ Thus, different from the case of unmodified native chitin, the protonation of the abundant, randomly distributed C2-NH_2_ groups in chitins that are partially deacetylated in acid conditions provides a high density of cationic charges on the crystalline nanofibrils, which is a critical driving force for mechanical individualization of ChNF (Figure 14a). A typical procedure was applied on crab shell α-chitin by using 33% NaOH at 90 °C for 4 h, yielding 85–90% of partially deacetylated chitin with a DA of approximately 0.70 while still maintaining the crystallite properties of the original α-chitin.^272^ The results indicate that partial deacetylation took place selectively on the α-chitin crystallite surface. When the obtained deacetylated chitin was subjected to mechanical defibrillation and subsequent ultrasonication in an acidic medium (pH 3–4 using acetic acid), a viscous and transparent ChNF water suspension was obtained with nanofiber lengths of ∼250 nm and widths of ∼6 nm (Figure 15a). ChNF of more than 500 nm in length was also produced. Compared with the mechanical treatment of α-chitin without deacetylation, this method leads to ChNF under low-energy consumption and high nanofibrillation efficiency. In addition to ultrasonication, microfluidization was also used to nanofibrillate the partially deacetylated α-chitin into ChNF in acidic medium, which specifically enabled the production of ChNF with a large aspect ratio (length of several micrometers and width of approximately 30 nm) (Figure 15b).^273^ The control of NaOH-mediated deacetylation is critical in developing ChNF properties. By adjusting the NaOH reaction time, a series of deacetylated α-chitin with varying DDA was produced.^274^ More importantly, it was confirmed that deacetylation occurred on the surface and that the interior of nanofibers remained unaltered. When subjecting such modified α-chitins into wet pulverization in acetic acid media, ChNF was obtained, exhibiting a reduced width as the DA was reduced, which indicated that the physicochemical properties of ChNF were controlled by the deacetylation reaction. Apart from deacetylation, the acid type, pH, and ionic strength during mechanical nanofibrillation determine the nanofibrillation efficiency and properties of ChNF. For instance, after ultrasonication of α-chitin from crab shells, under different aqueous environments, the ChNF produced using ascorbic acid showed a width of 2.5 nm at pH 3.5 and low ionic strength, thereby indicating the conditions that favor chitin nanofibrillation: low pH, high ionic strength, and highly deprotonated polyvalent acids.^275^

Following TEMPO-mediated oxidation, the more active C6 primary hydroxyl groups on the crystallite chitin surfaces are selectively oxidized,^276^ and new β-(1, 4)-linked polyuronic acids, with repeating units of sodium salt of *N*-acetylglucosaminuronic acid, i.e., chitouronic acid, are obtained quantitatively (Figure 14b). Thus, similar to partial deacetylation, the formation of carboxylate groups on the chitin surface is crucial for its individualization into ChNF, wherein negative charges enhance the mechanical effect to deconstruct the chitin bundles through electrostatic repulsion and/or osmotic effects, in a similar manner compared to that of TEMPO-oxidated CNF.^277^ For instance, a TEMPO/BrNa/NaClO system was used to oxidize α-chitin from crab shells (pH 10 at room temperature) and the obtained TEMPO-oxidated chitin was individualized in water by continuous ultrasonication in only a few minutes, eventually forming ChNF with length of the order of 1 μm.^278^ Following the same procedure, TEMPO-oxidation of highly crystalline β-chitin from tubeworms, combined with ultrasonication, resulted in ChNF with length of several micrometers and width of 20–50 nm (Figure 15c).^279^ To extend the use of TEMPO oxidation, zwitterionic ChNF, containing cationic amino and anionic carboxylate groups, was prepared by sequential chemical modification of crab α-chitin via partial deacetylation and TEMPO-mediated oxidation (TEMPO/NaClO_2_/NaClO system), followed by a mild mechanical treatment.^280^ Particularly, such TEMPO/NaClO/NaClO_2_ system was used in a weakly acidic medium (pH 6.8), rather than alkaline condition for common TEMPO system. This is because after applying partial deacetylation, TEMPO oxidation at pH 10 was found to be too intense and caused severe depolymerization of chitin, resulting in a low yield. Indeed, TEMPO-mediated oxidation is hardly used to produce carboxylated ChNF from squid pen β-chitin, likely due to the low DDA in such chitin source and its low crystallinity. Hence, an oxidation system containing ammonium persulfate (APS), combined with ultrasonic disintegration under acid conditions, was applied to produce ChNF from squid pen, resulting in ultrafine ChNF (2–4 nm width and several micrometers in length).^281^ More importantly, when 45 wt % APS was used to react with β-chitin, the carboxylate content of ChNF reached 0.802 mmol/g, a higher value compared to that of common TEMPO-oxidized samples.

While partial deacetylation and TEMPO-mediated oxidation facilitate mechanical nanofibrillation of chitin, they are not designed to install other functional groups on ChNF. Esterification of chitin surfaces with anhydrides in situ, prior to mechanical nanofibrillation, endows groups that simultaneously loosen the nanofiber bundles, further facilitating the mechanical fibrillation.^282^ For instance, superficial esterification with maleic anhydride to α-chitin that originated from crab shells significantly improved mechanical disintegration in water into uniform 10 nm width ChNF with a degree of substitution of 0.25 and well preserved crystalline structures (Figure 15d).^283^ This is explained by the formation of carboxylate groups on the surface of chitin that generated strong electrostatic repulsion and osmotic pressure during mechanical fibrillation. When applying ultrasonication to disintegrate maleic-anhydride-esterified chitin into ChNF, the smallest averaged nanofiber width was 15 nm; meanwhile, the width could be adjusted by tuning the ultrasound treatment (time and power).^284^ In addition to maleic anhydride, acetic anhydride has been used in a one-step esterification of the hydroxyl groups of α-chitin in situ in dimethylformamide by using ball milling (200 rpm), resulting in individualized ChNF (20–44 nm in width).^285^ After acetylation, the resulting nanofibers were stably dispersed in chloroform. These results demonstrate the efficiency and versatility of esterification treatments to produce ChNF with several additional benefits.

At present, although ionization of chitin surfaces shows great promise, strong mechanical treatment is still required to produce ChNF, which may be caused by the heterogeneous surface ionization. Indeed, both partial deacetylation and oxidation reactions proceed in a limited manner since the tightly bounded nanofibrils in the chitin bundles restrict accessibility to the inner nanofibrils.^286^ Thus, a more efficient process that enables thorough ionization is needed. To this end, a method involving preswelling of α-chitin with a mixture of glycerol and sulfuric acid, followed by colloid milling, was developed and found effective to break the interfibrillar H-bonding of chitin and adding surface charges.^287^ This process made the swollen α-chitin easier to disassemble into ChNF during grinding, resulting in ChNF with a width of 50 nm and micrometer in length. Beyond a simple swelling treatment, an energy-efficient method for isolating ChNF from various chitin sources was developed using pseudo-solvent-assisted maleic anhydride intercalation, which circumvented the need for strong mechanical disintegration (e.g., hand-shaking was sufficient) (Figure 14c1).^288^ In this method, a dimethyl sulfoxide/potassium hydroxide mixture, which barely dissolved chitin but disrupted intermolecular H-bonding, was effective in splitting closely packed nanofibril bundles into entangled nanofibril networks (Figure 14c2 and c3).^289^ Thus, preswelling facilitated a more open structure and improved the accessibility and diffusion of reactive maleic anhydride to the interior of the initial structures, resulting in a homogeneous and rapid surface ionization (esterification) of chitin and breaking the H-bonding between adjacent nanofibrils (Figure 14c2). More importantly, the esterified chitin surface not only expanded the interlayer spacing but prevented recombination. As a result, the self-exfoliated ChNF was 4–5 nm in width and had a high aspect ratio, up to 103 (Figure 15e). More uniquely, it possessed controllable thickness (0.8–2.2 nm), only a few molecular layers (single layer is roughly 0.5 nm). This self-exfoliation concept, together with its high yield, energy efficiency, and scalability, is a promising route for isolating ChNF in industrial settings.

One principal disadvantage of chemical modification is that the process might use chemicals that are not environmentally friendly. As such, enzyme modification, e.g., by using oxidases, deacetylases, or specific hydrolases, may be attractive options.^291^ For instance, a biocatalytic method based on chitin deacetylase produced by *Acinetobacter schindleri* MCDA01 was recently used to produce partial deacetylated α-chitin.^290^ In the deacetylation process, C2-acetamido groups on the surface of chitin crystalline fibrils were exposed and specifically hydrolyzed by the chitin deacetylase, which produced a nucleophile attack on the carbonyl carbon in the acetyl group during the catalysis of C2-acetamido groups to C2-amino groups (Figure 14d).^292^ As a consequence, after applying a combination of homogenization and ultrasonication in acidic medium, nanofibers (length of several micrometers and width of 25–45 nm) were obtained with a DDA of 32% and crystallinity of 89% (Figure 15f). Compared with the ChNF obtained from chemical-mediated partial deacetylation, longer nanofibers (aspect ratio up to 1000) were readily achieved by the enzymatic process. The enzymes specifically catalyzed the hydrolysis of C2-acetamide groups in chitin, and limited partial degradation of polysaccharide chain occurred, resulting in wider and longer nanofibers following the mild process. Meanwhile, edge or intermediate surfaces of ChNF from the chemical partial deacetylation would be degraded into the water-soluble fractions by using a hot alkali solution, thus collapsing them into fragments.^272^

#### 3.2.2 Isolation of Chitin Nanocrystal, ChNC

As is the case for the production of CNC,^27^ the isolation of ChNC requires the removal of the amorphous domains of chitin, which loosens the fibrillar structure and further enables an easier nanofibrillation via mechanical treatment (Figure 12b). Compared with ChNF, the main characteristics of ChNC is that it is assembled from well-ordered chitin structures,^293^ resulting in a higher crystallinity.^25^ Moreover, ChNC has a rodlike morphology with a short length and small aspect ratio.^294−296^ In principle, strong acid hydrolysis and chemical oxidation are the two primary methods used to produce ChNC, while other top-down strategies, e.g., acidic deep eutectic solvent treatment, are also feasible.^297^ The nanofibrillation efficiency and characteristics of ChNC, including morphology, crystalline form, crystallinity, etc., depend on both the chitin source and preparation method, thus affecting its physicochemical performance. However, a well-known disadvantage of these methods is the low yield given the loss of amorphous material and chitin depolymerization.^298^

##### 3.2.2.1 Strong Acid Hydrolysis

**Figure 16:**
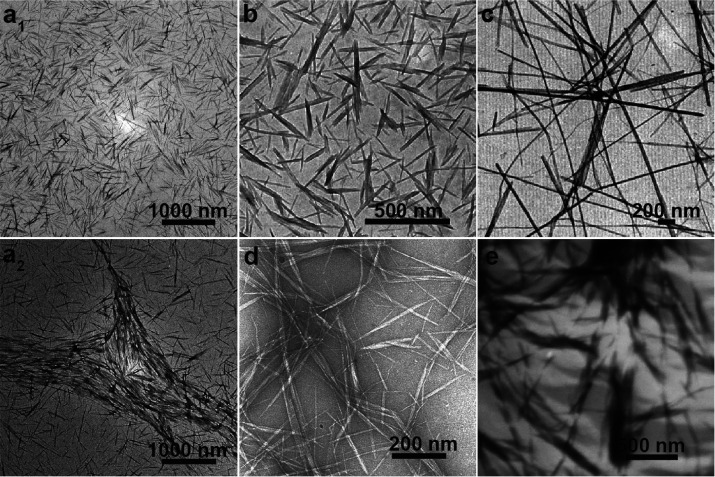
TEM images of HCl-hydrolyzed ChNC from crab shell α-chitin dialyzed in suspension at (a1) pH 3 and (a2) pH 6 followed by 10 min of ultrasonication. TEM images of HCl-hydrolyzed ChNC from (b) crab shells and (c) *Riftia* tubes. Adapted from refs (306) and (312). Copyright 2003 and 2002, respectively, American Chemical Society. TEM images of ChNC isolated from crab shell α-chitin using (d) TEMPO-mediated oxidation and (e) ammonium persulfate treatment. Adapted from ref (320). Copyright 2008 American Chemical Society. Adapted with permission from ref (327). Copyright 2017 Elsevier.

The first report regarding acid hydrolysis of chitin dates back to 1959, where purified α-chitin from crab shells was hydrolyzed with 2.5 M HCl during 1 h using a reflux system, followed by homogenization (three passes).^299^ The process resulted in a stable ChNC suspension that exhibited nematic liquid crystal (LC) ordering. Following this development, HCl was considered as an option for acid hydrolysis of chitin into ChNC. In studies, 64% sulfuric acid and maleic acid have also been used to hydrolyze chitin from crab and prawn shells, respectively, endowing ChNC with negative charges.^300,301^ In recent decades, upon optimization of the isolation conditions, a typical procedure for acid hydrolysis of chitin involves (1) HCl (e.g., 3 M HCl) at the boiling point (90–105 °C) under vigorous stirring for a certain amount of time (0.5–9 h) to hydrolyze chitin; (2) diluted as-prepared chitin/acid mixture with water to quench the reaction; (3) separation (filtration and centrifugation) and purification (dialysis) steps to remove the dissolved components and impurities in the suspension; and (4) mechanical treatment (e.g., ultrasonication) to enable full dispersion of ChNC in water.^302,303^ Compared with ChNF production, the preparation of ChNC involves more steps, often resulting in less precise control of the hydrolysis and subsequent nanofibrillation. Indeed, acid concentration and hydrolysis time as well as the strength of mechanical treatment and DA of the native chitin all influence the properties of the as-prepared ChNC.^304^ For instance, when subjecting α-chitin to HCl hydrolysis at 105 °C and 3 h three times, the crystallinity of obtained ChNC decreased rather than increased compared with native α-chitin. This is attributed to the harsh conditions used for hydrolysis that converted the crystalline structures to amorphous ones.^305^ After hydrolysis, dialysis of the ChNC suspension is conducted, which affects the pH of the system and further influences the effectiveness and quality of the ChNC aqueous suspension. This is because, to facilitate dispersion of nanocrystals via mechanical treatment, electrostatic repulsion originating from the protonated amino groups on the ChNC surfaces is required and is highly dependent on the pH of the system.^293^ For example, the nanocrystals were well dispersed after 10 min of ultrasonication when the suspension after dialysis had a pH of 3 (following hydrolysis of α-chitin with 3 M HCl for 30 min) (Figure 16a1). However, when following the same processing condition at pH = 6, clusters of nanocrystals, together with individually dispersed nanocrystals, coexisted in the system (Figure 16a2). Such irreversible aggregation of nanocrystals, even after ultrasonication, was induced by the decreased electrostatic repulsion between nanocrystals at high pH after dialysis (less protonation of amino groups) and was further strengthened by interparticle interactions.

**Figure 17:**
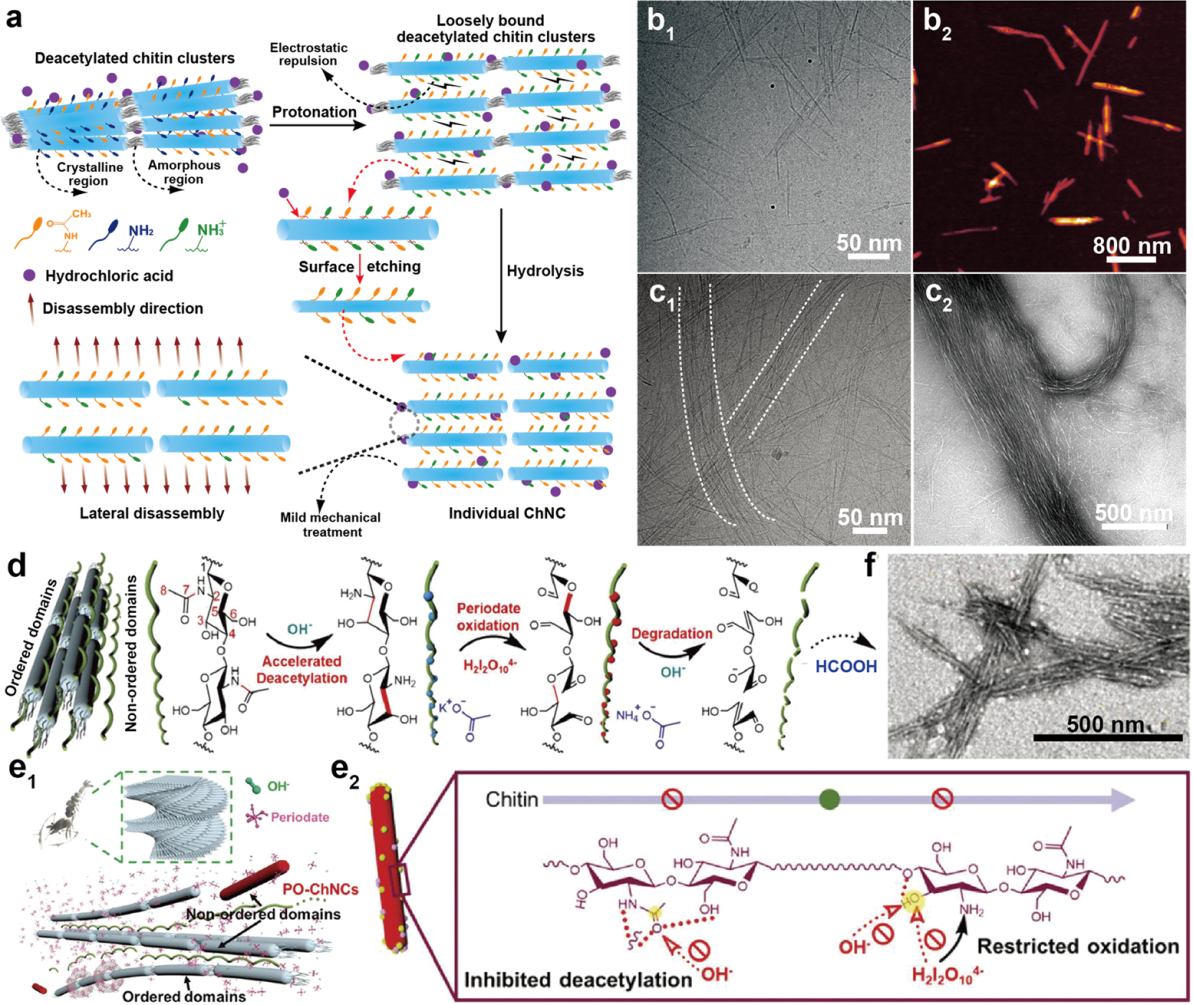
(a) Schematic illustration of nanofibrillation of deacetylated chitin clusters upon acid hydrolysis, generating single, individual ChNC. (b1) Cryo-TEM and (b2) AFM images of individual ChNC obtained by 90 min acid hydrolysis. (c1) Cryo-TEM of 30 min acid-hydrolyzed ChNC after dialysis and in the absence of ultrasonication. (c2) TEM image of 60 min acid-hydrolyzed ChNCs after dialysis and 30 s ultrasonication. The dashed lines in (c1) are added to indicate loosely bound chitin nanofibrils. Adapted from ref (315). Copyright 2020 American Chemical Society. (d) Proposed pathway for the accelerated deacetylation, oxidation, and degradation of nonordered domains in chitin upon periodate oxidation, resulting in soluble compounds containing carboxylic groups. This process demonstrates the capability of periodate to produce ChNC. Schematic representation of the (e1) production of ChNC by selective alkaline periodate oxidation of the nonordered chitin domains while keeping the ordered domains intact and (e2) restricted deacetylation and alkaline periodate oxidation on ChNC surface upon disintegrating chitin into ChNC, leading to zwitterionic nanocrystals. (f) TEM image of ChNC obtained from shrimp chitin. Adapted with permission from ref (328). Copyright 2021 The Royal Society of Chemistry.

Following acid hydrolysis for ChNC production, the characteristic dimensions of ChNC include width of 10–50 nm, length of 100–600 nm, and aspect ratio of approximately 15 (Figure 16b),^306−310^ and the corresponding specific surface area can be up to 350 m^2^/g.^311^ Uniquely, ChNC that was isolated from *Riftia* tubes following the hydrolysis of purified chitin with 3 M HCl at boiling during 1.5 h stirring reached an average length of 2200 nm and width of 18 nm (Figure 16c), which resulted in a large aspect ratio (approximately 120), enabling novel applications.^312^ However, the spindlelike shape of ChNC was obtained from acid hydrolysis (Figure 16a–c), rather than individualized, straight rodlike particles. Given the insolubility of chitin, acid treatment would mainly result in surface exfoliation,^313^ with little effect on the acetyl groups on the chitin’s surface, as confirmed by the unaltered DA before and after acid hydrolysis.^311^ Moreover, the interior nanofibrils in the chitin bundles are more resistant to acid, and etching is limited by accessibility of acid molecules. Thus, insufficient etching of the recalcitrant chitin surfaces leads to a nonhomogeneous reaction; i.e., the more accessible, disordered, and weakly bound amorphous region is first removed upon hydrolysis.^314^ More importantly, the strong H-bonding between nanocrystals prevents a complete individualization of ChNC. To overcome this difficulty and to produce individualized ChNC, a method involving HCl hydrolysis of partially deacetylated α-chitin (DDA of about 28%) was recently developed.^315^ Following such process, positively charged amino groups were present on the chitin surfaces at the very beginning of acid hydrolysis reaction, which generated strong interfibrillar electrostatic repulsion, leading to a more open structure via loosening the tightly bound nanofibrils. This effect promoted access of HCl or water molecules to the inner nanofibrils, and eventually achieved lateral disassembly in an even manner (Figure 17a). Particularly, the surface deacetylation of chitin prevented the intermolecular H-bonding and broke the regularity of lateral packing between chains given the enhanced interaction of chitin with water,^316^ while the crystallinity of ChNC remained largely unaltered.^317,318^ Such process induced homogeneous surface etching of chitin, leading to isolation of individualized, straight rodlike nanocrystals (Figure 17b), which is in contrast to those obtained from direct acid hydrolysis, as shown in Figure 16a–c. Moreover, the effect of charged surfaces on the disassembly of chitin clusters was further emphasized by the coexistence of well-fibrillated, individual ChNC with loosely bound chitin nanofibrils by limited ultrasonication after dialysis (Figure 17c).

##### 3.2.2.2 Strong Oxidation

Strong chemical oxidation is an alternative route to isolate ChNC. As described in Figure 14b, by subjecting chitin to chemical oxidation reaction, e.g., the TEMPO system, the water-insoluble chitin is converted to water-soluble products (following selective oxidation of the more active C6 primary hydroxyl groups), which removes the amorphous domains while keeping the crystallite regions intact.^319^ For instance, a TEMPO-mediated oxidation system composed of TEMPO/NaBr/NaClO was used to oxidize crab shell α-chitin in water at pH 10 to produce ChNC.^320^ The electrostatic repulsion and osmotic effect between anionically charged nanocrystals generated from carboxylate groups on the chitin surface enabled further individualization under mechanical treatment, resulting in needlelike ChNC with a length of 340 nm and a width of 8 nm (Figure 16d). Chemically, no deacetylation occurred in ChNC and the crystallinity remained similar to that of the original chitin, indicating that the C6 carboxylate groups formed by TEMPO-mediated oxidation were present only on the surfaces of chitin crystallites. In addition to the ChNC directly isolated from oxidization, strategies combining TEMPO-mediated oxidation with pre- or post-treatments via partial deacetylation have been developed for ChNC production.^321^ With this process, amphoteric ChNCs were isolated,^322^ showing similar charge distribution as that of zwitterionic ChNF. In a typical two-step treatment, 30% NaOH was first used to partially deacetylate α-chitin, followed by strong TEMPO/NaClO_2_/NaClO oxidation at pH 6.8 and 60 °C for 4 h, leading to ChNC after 10 min ultrasonic treatment.^323^ To reduce consumption of chemicals, the TEMPO-mediated oxidation system was modified by implementation of laccase enzymes,^324^ a glycosylated oxidase that contains four copper atoms in the active site, which catalyzes one-electron oxidation of small molecules.^325^ Using the O_2_/laccase/TEMPO system to oxidize α-chitin, associated with ultrasonication, ChNCs (length of 480 nm and width of 24 nm) were isolated, wherein TEMPO acted as a mediator to facilitate the oxidation of chitin by transporting an electron.^326^

Besides TEMPO-mediated oxidation, other chemical oxidation systems have been considered to produce ChNC. For example, APS was used as an oxidizing agent to produce ChNC.^327^ Oxidative reaction to remove amorphous domains of chitin can be carried out through the formation of free sulfate radicals, hydrogen sulfate, and hydrogen peroxide, simultaneously converting the hydroxyl groups at the active C6 on chitin surfaces into carboxylic groups. The resultant ChNC had a length of 400–500 nm and a width of 15 nm with a crystallinity index of 93.5% (Figure 16e), similar to that of ChNC obtained from TEMPO-mediated oxidation. In a recent effort, a 14 day, one-pot alkaline periodate oxidation reaction was used to produce ChNC at ambient temperature.^328^ The selective oxidation reaction of the disordered domains of chitin was induced by dimeric orthoperiodate ions, a major oxidizing agent in alkaline condition, significantly promoting sequential reactions involving accelerated deacetylation, oxidation, and degradation, which eventually converted amorphous domains of chitin into soluble compounds (Figure 17d). Thus, nonordered domains of chitin were selectively removed while keeping the ordered, crystalline domains intact (Figure 17e1). Uniform ChNCs exhibiting a typical needlelike morphology with an average length of 242 nm and width of 12 nm were isolated through this method (Figure 17f), with a yield of 50 wt % (nearly half of the nonordered regions were dissolved upon oxidation). A particular mechanism leading to stable ChNC in such a long period of oxidation relied on the strongly restricted deacetylation and cyclization on the ChNC surfaces in alkaline periodate oxidation at room temperature (Figure 17e2). The deacetylation of ChNC was inhibited by steric hindrance and by inter- and intramolecular H-bonding on oxygen and nitrogen in the acetamido groups. Once a few of the acetamido groups were converted into amine groups on the ChNC surfaces, the periodate oxidation was restricted,^329^ which was caused by the blockage of the C3-hydroxyl groups of chitin chains in the ordered domains, given their integration in numerous hydrogen bonds.^330^ Since general acid–base catalysis of cyclization between periodate ions, hydroxyl groups, and amine groups of oxidation requires activated hydroxyl groups on C2 or C3, limited activation of the blocked protons at the C3-hydroxyl groups hampered the occurrence of the cyclization reaction on the surface of ChNC (Figure 17e2). To conclude, such novel oxidation method for ChNC isolation broadens the available toolbox for nanochitin production.

In summary, a variety of methods have been applied to deconstruct α- and β-chitin into ChNC with different morphologies, dimensions, surfaces, and physicochemical properties (Figure 12b). Some drawbacks can be highlighted in relation to these methods: First, most of them require heating to accelerate the reaction. Second, corrosive concentrated acids or alkali solutions as well as highly reactive chemicals are commonly used. Moreover, the process often involves multiple stages, high energy input, and long cycles. Hence, developments to address the challenges associated with ChNC production are highly desirable, for instance, to advance efficient, mild, environmentally friendly, and cost-effective routes.

### 3.3 Engineering Nanochitin

To use nanochitin as a building block for multilevel assembly, both its chemical and morphological properties should be reproducible and tailorable.^60,331^ Several methods have been used to isolate nanochitin in a couple of different forms with distinctive chemical and morphological features. While the isolation process is suited to the characteristics of the chitin sources,^332^ the derived nanosized materials exhibit somewhat similar chemical properties. For example, regardless of the isolation method used, most types of nanochitin contain abundant surface hydroxyl, acetyl, and amine groups,^15,20,30^ which in some cases might be an obstacle for conversion of nanochitin into advanced materials. As a consequence, engineering nanochitin can be useful to tailor its chemistry, particularly by installing functional surface groups that bring new attributes.^333^ Surface hydrophobic groups can be added to nanochitin to reduce surface energy, facilitating dispersion and interaction with nonpolar media.^161^ We discuss next the main chemical strategies to engineer the surface chemistry of nanochitin, which uncovers functions to make nanochitin more suitable for adoption in material design and development.

#### 3.3.1 Chitin’s Hydroxyl Groups

**Figure 18:**
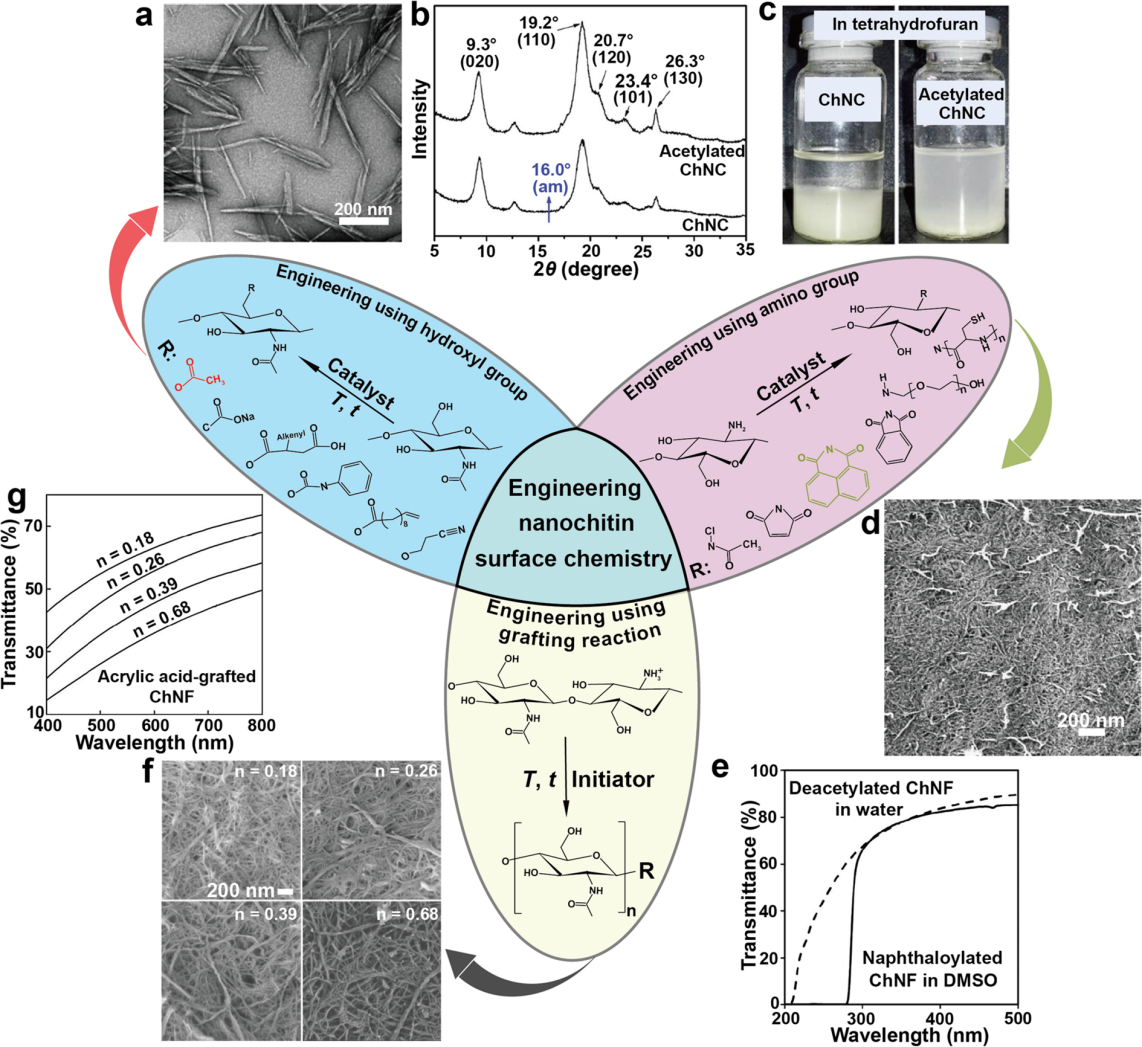
Chemical strategies used to tailor the surface chemistry of nanochitin. Hydroxyl groups: (a) TEM image of acetylated ChNC. (b) X-ray diffraction patterns of ChNC and acetylated ChNC. (c) Dispersibility of ChNC (after 30 min standing at 4 °C) and acetylated ChNC (after 12 h standing at 4 °C) in tetrahydrofuran. Adapted with permission from ref (337). Copyright 2014 The Royal Society of Chemistry. Amino groups: (d) SEM image of naphthaloylated ChNF with 14% substitution. (e) Transmittance spectra (0.1 w/v% in water) of deacetylated ChNF (dashed line) and naphthaloylated ChNF in dimethyl sulfoxide (solid line). Adapted with permission from ref (347). Copyright 2014 Elsevier. Grafting: (f) SEM micrographs of AA-grafted ChNF. (g) UV–vis transmittance spectra of AA-grafted ChNF in basic water. *n* refers to the molar ratio of grafted AA against an *N*-acetyl glucosamine unit of a ChNF. Adapted with permission from ref (354). Copyright 2012 Elsevier.

Similar to other nanopolysaccharides, nanochitin surface is covered with abundant hydrophilic hydroxyl groups that are amenable for modification with functional groups (Figure 18).^295^ Specifically, two characteristic hydroxyl groups are present in chitin, showing different reactivity. The C6 hydroxyl group is generally regarded as more active than those linked to C3.^334^ For instance, the C6 primary hydroxyl groups on ChNC surfaces can be regiospecifically oxidated to carboxylate groups by TEMPO-mediated oxidation through the formation of intermediate aldehyde structures, leading to anionic charges.^298,319^ It should be noted that deacetylation is not expected to occur by oxidation of surface acetyl groups. The dispersion of nanochitin in organic media is challenged by its inherent hydrophilic character. Moreover, some level of deacetylation is bound to occur during nanochitin isolation, converting the acetamide groups into amino groups. This effect increases the hydrophilicity of nanochitin and hence further prevents its dispersion in nonpolar solvents. The latter effect can be counteracted by introducing hydrophobic groups via chemical modification, also improving adhesion with hydrophobic matrices in composites.^15,163^ For instance, long hydrocarbon chains, such as those in 10-undecenoyl chloride, can be covalently attached to the hydroxyl groups on nanochitin, improving its dispersion and compatibility with organic solvents.^335^ The attachment of reactive allyl groups onto nanochitin further induces thiol–ene click cross-linking reactions, diversifying the possible products that can be derived from nanochitin. Apart from long-chain molecules, the surface of ChNC can be chemically engineered using a chemical reaction between small molecules and the hydroxyl groups, for instance using isocyanate groups from the phenyl and isopropenyl-α,α-dimethylbenzyl isocyanate, as well as alkenyl succinic anhydride.^163^ The produced acylated ChNCs exhibit enhanced dispersibility in solvents of medium to low polarity. Acetylation using acetic anhydride is also efficient in engineering the surface chemistry of nanochitin.^285,336^ Through controlled acetylation, the hydroxyl units on ChNC can be replaced by hydrophobic acetyl groups, a process that occurs in heterogeneous conditions and gradually advances from the surface to the core of the material.^337^ After surface acetylation, the acetylated ChNCs exhibit a rodlike morphology (Figure 18a) and maintain, to a large extent, similar structural and dimensional properties as well as crystallinity of the original ChNC (Figure 18b). Acetylated ChNC displayed outstanding dispersion and stability (measured during 12 h) in tetrahydrofuran, which was in contrast with the original ChNC that settled after 30 min (Figure 18c).

Engineering nanochitin’s surface chemistry also brings additional chemical attributes,^338^ for instance, the possibility of dual or multiple functions that enable advanced nanochitin-derived materials. Following this concept, a two-step chemical modification was applied,^339^ wherein thiol groups of 3-mercaptopropyl trimethoxysilane were linked to the hydroxyl groups of the ChNC or mediated by tetraethyl orthosilicate,^340^ followed with attachment of long-chain heptadecafluorodecyl acrylate to the existing thiol groups. The reduced surface free energy and morphological features of the fluorinated ChNCs generated superior amphiphobicity and substrate-independent, mechanically stable coatings. Other example is that a chemical strategy can link cyanoethyl groups onto the surface hydroxyl groups of ChNF via Michael addition reaction of acrylonitrile in alkaline aqueous media.^341^ The Michael addition reaction between hydroxyl groups and acrylonitrile was nonregiospecific to the C6 primary hydroxyl groups of ChNF. An application as a battery separator was explored for cyanoethyl ChNF, which not only exhibited a high ionic conductivity but also retained excellent mechanical strength compared with the unmodified ChNF.

#### 3.3.2 Chitin’s Amino Groups

Following the disintegration of native chitin, which involves predeacetylation, many of the surface acetyl groups are converted to amino groups, increasing ChNF dispersibility in water given the protonation and improved electrostatic repulsion.^272^ Thus, the combined high reactivity of amino groups and the enhanced dispersibility in acidic aqueous media of surface-deacetylated nanochitin provide a range of facile and efficient strategies to engineer nanochitin under homogeneous reaction conditions (Figure 18).^342^ For instance, *N*-halamines can be produced, exhibiting some attractive functions, such as rechargeability, resistance to microorganisms, and nontoxicity to humans, also in association with amine, amide, and imide groups,^343^ providing the possibility to engineer nanochitin amino groups. Through control of the reaction with diluted sodium hypochlorite solution, *N*-chlorination of ChNF was achieved by substituting the N–H bond with the N–Cl bond. In this process, the concentration of sodium hypochlorite and reaction time strongly affect the active chlorine content on ChNF.^344^

In chitosan chemistry, protection of the reactive amino groups at the C2 position is possible using various types of anhydrides (e.g., phthalic anhydride).^345^ Taking advantage of the similarity in surface chemistry, such reactions are useful to enhance ChNF dispersion in water. For instance, *N*-phthaloylation, *N*-maleylation, and *N*-naphthaloylation of surface-deacetylated ChNF were obtained chemo-selectively and quantitatively in aqueous media via linking of phthalic, maleic, and naphthalic anhydrides to the amino groups.^346,347^ The ChNF network structure was maintained after the reaction, similar to those formed by the original ChNF and related to the microstructure of naphthaloyl ChNF (Figure 18d). The modified ChNF produced homogeneous dispersions in several organic solvents; in particular, naphthaloyl ChNF showed enhanced dispersion in low-polarity aromatic dimethyl sulfoxide (Figure 18e), which was attributed to the high level of solvation with the phthaloyl group. More uniquely, dispersion of the naphthaloyl ChNF in aromatic solvents showed a reversible disperse-to-precipitate transition at approximately 29 °C, which was probably due to reversible changes in the interactions between the introduced functional groups and the given aromatic solvent.

Besides surface treatment of nanochitin with anhydrides, acylation of the amino groups, e.g., by forming amide bonds, is one of the most important routes for nanochitin modification.^348^ For instance, cysteine was grafted onto ChNF through the formation of amide bonds between the amino groups and the cysteine carboxylate groups mediated by *N*-(3-(dimethylamino)propyl)-*N’*-ethylcarbodiimide hydrochloride (EDC) and *N*-hydroxysuccinimide. The process resulted in the production of thiol-functionalized ChNF.^349^ Meanwhile, the morphology of modified ChNFs remained almost unchanged, an advantage compared to those obtained after modification with thiol groups. Most of amidation reactions, including those mediated by EDC, are based on the nucleophilic attack of the amino groups to electrophilic carbons in activated carboxyls.^350^ Hence, nanochitin amidation may be challenged since the amino groups confined on the surface have limited mobility. A strategy to overcome this issue is to use reductive amination interaction between the primary amino groups on deacetylated ChNC and the terminal aldehyde groups of monomethoxy poly(ethylene glycol) (PEG).^351^ Due to the fine dispersibility of ChNC in water, the reaction readily proceeds in aqueous media under mild, weak acidic conditions at room temperature. The obtained PEG-modified ChNCs exhibit high stability against electrolytes given the enhanced steric repulsion endowed by PEG. Interestingly, although the number of amino groups increased with deacetylation time, the number of PEG units linked on the surface of ChNCs remained relatively constant, 0.2–0.3 g/g ChNC. This effect is explained by the saturation of reaction sites, namely, the long PEG chains that attached to ChNC in a confined area induced chemical and spatial hindrance, limiting the accessibility of amino groups to the terminal aldehyde groups of free PEG.

#### 3.3.3 Surface Grafting

Grafting reactive components, e.g., vinyl polymer, on the backbone of nanochitin takes advantage of the chemical activity of chitin, providing another route to tailor its surface (Figure 18). For instance, high-density polymers have been grafted onto nanoparticles, which effectively changed the surface properties.^352^ For example, following the “grafting-from” concept, a copolymer consisting of ChNC-*graft*-polycaprolactone (ChNC-*g*-PCL) was synthesized by initiating the ring-opening polymerization of caprolactone monomer onto ChNC surfaces under microwave radiation, using tin(II) octoate as the catalyst.^353^ This covalent linkage produced a new type of ChNC bearing PCL brushes, which resulted in hydrophobic ChNC, facilitating applications as an enhancer of formulations of polymer-based nanocomposites.

A facile radical polymerization that was initiated by potassium persulfate in aqueous media was used to graft organic molecules from ChNF.^354^ The grafting-from process involved sulfate radicals that reacted with ChNF to form chitin radicals along the chitin polymer backbone, and the amorphous part of the nanofibers initiated graft copolymerization of acrylic acid (AA). The microstructure of AA-grafted ChNFs slightly changed after the reaction, for instance, by increasing the molar ratio of grafted acrylic acid relative to the *N*-acetyl glucosamine units of the ChNFs (Figure 18f). Eventually, the resultant ChNFs were efficiently dissociated and were dispersed homogeneously in basic water, showing relatively high transmittance even when using ChNF with a high grafting degree of AA (Figure 18g), given the electrostatic repulsion effect brought by the AA-grafted nanofibers.

In sum, surface engineering of nanochitin, either by reaction of the existing functional groups or by grafting (under given conditions of temperature, time, and catalyst), provides a versatile platform to endow new functionalities, further expanding the uses of nanochitin (Figure 18). Unfortunately, more often than not, multiple steps are required to chemically modify nanochitin. Thus, further investigation on this topic should emphasize versatile strategies that enable one-step, customizable production of nanochitin, for instance, those that can be applied in situ during nanofibrillation.

## 4 Assembly of Nanochitin at Multiple Length Scales

**Figure 19:**
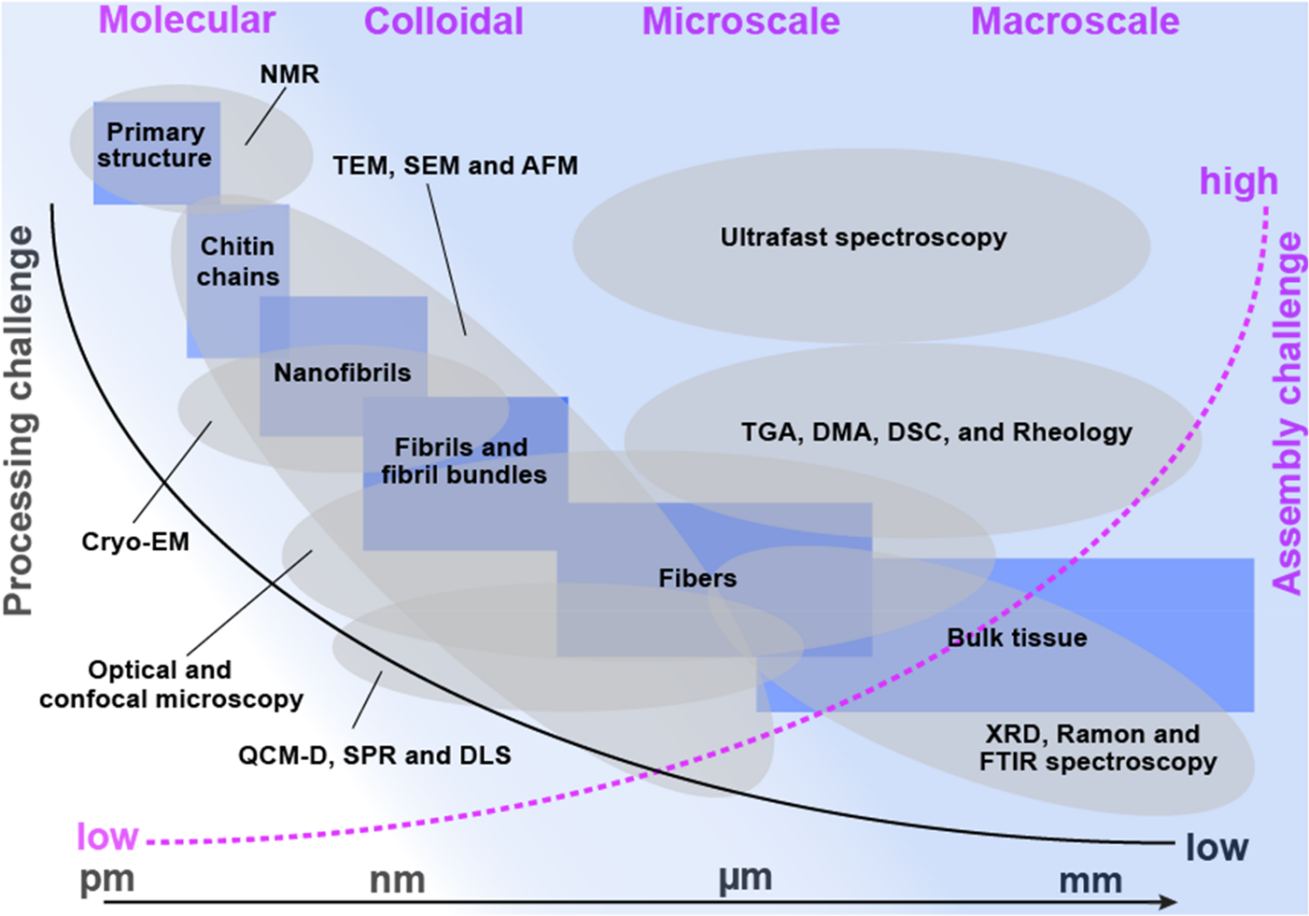
Hierarchal structures (blue boxes) and challenges for processing and assembly of (nano)chitin at different length scales, as noted. Experimental techniques that can be used for the design and characterization of nanochitin assemblies are introduced as a function of the characteristic length scale: NMR, nuclear magnetic resonance; TEM, transmission electron microscopy; SEM, scanning electron microscopy; AFM, atomic force microscopy; Cryo-EM, cryogenic electron microscopy; QCM-D, quartz crystal microbalance with dissipation; SPR, surface plasmon resonance; DLS, dynamic light scattering; TGA, thermogravimetric analysis; DMA, dynamic thermomechanical analysis; DSC, differential scanning calorimeter; FTIR, Fourier transform infrared spectroscopy; XRD, X-ray diffraction.

A common route to generate complex structures involves particle assembly guided by forces acting at multiple scales.^355,60^ The functions and applications associated with the structures are determined by the assembly process and the nature of the particles involved. However, it is difficult to identify the interactions relevant to the assembly, which are usually multiple in their origin and operate simultaneously. This applies to nanochitin since diverse chemical and hierarchical features influence the processability of chitin into nanochitin, resulting in a wide range of properties (Figure 19). For instance, even if the same source is used, treatments with small differences in conditions result in nanochitin of different sizes and surface properties.^315,356^ Hence, an in-depth understanding of nanochitin assembly with efficient and precise control is necessary before considering nanochitin as a material precursor. Here, different advanced techniques fit the needs in efforts to probe the properties of the material at different length scales, most relevant when it comes to understanding the assembly process (Figure 19). Computational techniques, *e.g*., molecular dynamics simulation,^22^ can be included as a useful approach to predict or study self-assembly. In this section, we discuss the interactions relevant to nanochitin assembly, the process and structures that are formed under given conditions, and multiscale factors, all of which inspire new routes for material design.

### 4.1 Multiscale Assembly

In nature, equilibrium structures formed by assembled particles follow the principles of thermodynamics. It is well accepted that at least two factors govern the assembly of particles: the particle shape or morphology and the forces acting during the assembly process.^355^ For nanochitin, the morphology is usually associated with rigid nanorods or flexible nanofibers. However, the assembly forces can be effective at multiple scales (Figure 2) and might operate simultaneously (Figure 19). Moreover, in addition to interaction forces involving nanochitin itself, other forces of relevance originate from the surrounding environment, for instance, involving water molecules and ions.^357,358^ These factors make the assembly of nanochitin difficult to predict or control. In this subsection, the interaction forces that operate at multiple scales as well as the resultant nanochitin assemblies are reviewed. We also discuss the structure–process–property relationships involved in nanochitin for material design and development.

#### 4.1.1 Molecular-Scale Interactions

Multiple forces act at the molecular level to control the assembly of chitin chains into fibril-like chitin nanoparticles. Generally, a synergy of H-bonding, vdW forces, electrostatic interactions, and hydrophobic interactions is applied.^60,359^ In most cases, these forces are strongly associated with the presence of water, with H-bonding being most relevant given the abundant hydrogen donors and acceptors available in nonionized groups of chitin chains. In the case of vdW forces, the polarity of accessible groups in chitin significantly affects the interactions. Particularly, when associated with H-bonding, vdW forces are critical in relation to supramolecular interactions that take place within the crystalline domains of nanochitin. This also applies to other nanopolysaccharides such as nanocelluloses.^360^ Compared with vdW forces and H-bonding, electrostatic forces are relatively stronger in magnitude and range. Nevertheless, the relatively weak but more dynamic nature of the former interactions exerts a more significant effect on the assembly of chitin chains, as well as the properties of formed nanochitin.

**Figure 20:**
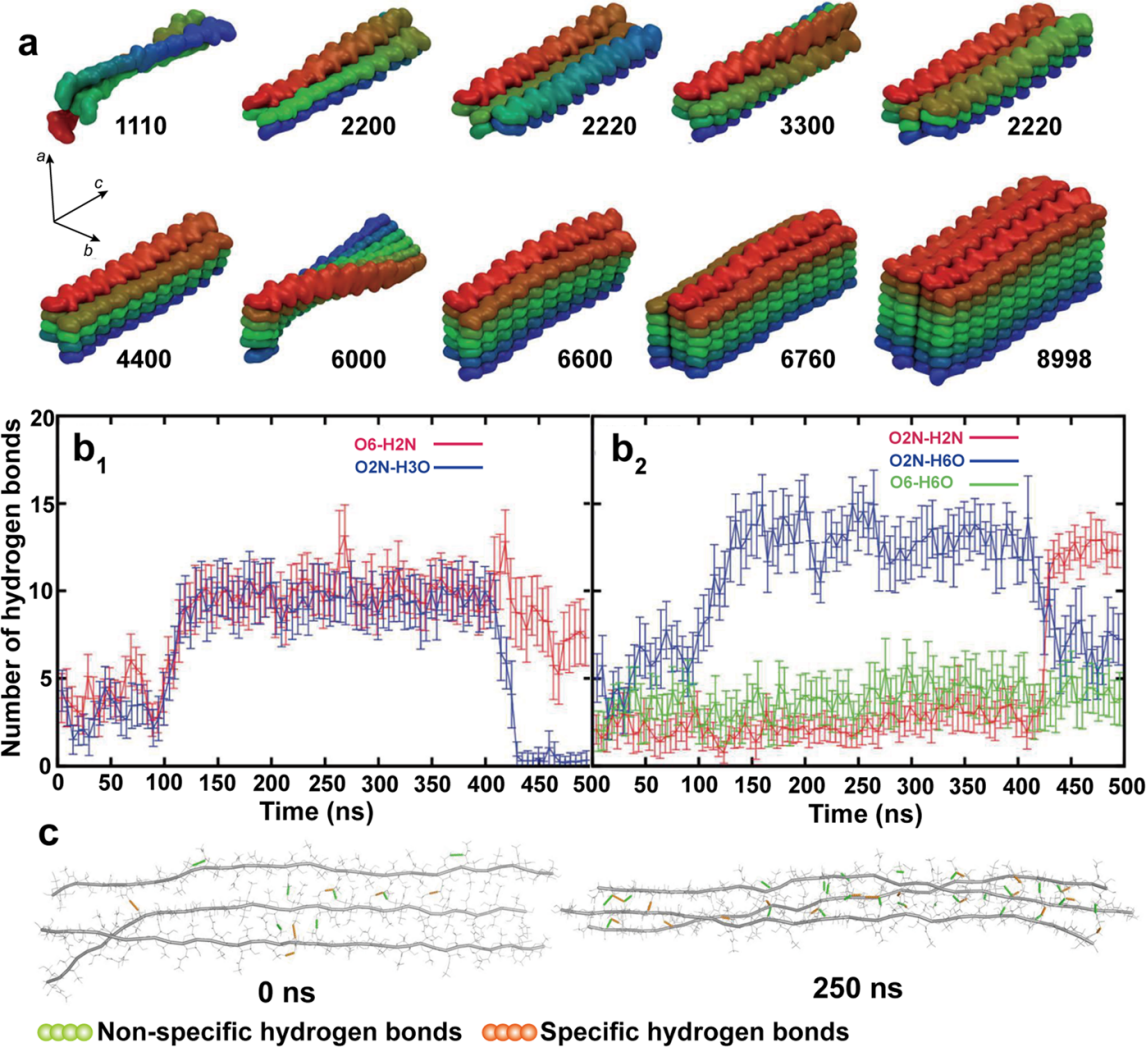
(a) Schematic illustration of average structures of simulated chitin nanofibril systems using color-coded building blocks to highlight structural features. The number of nonzero digits in the name is the number of chitin chains in the *ab* plane, whereas the numeral at a given position is the number of chains in the corresponding *ac* plane. Number of (b_1_) nonspecific and (b_2_) specific H-bonds emerging during simulation using the (1110) model. Lines represent running averages over 5 ns time intervals, and vertical bars are variances in the same time interval. (c) Selected structures of the (1110) system observed at 0 (left panel) and 250 ns (right panel) during the MD simulations with highlighted H-bonding. Nonspecific H-bonding shows green and specific ones show orange. The chain backbones are shown as gray tubes. Adapted with permission from ref (316). Copyright 2016 The Royal Society of Chemistry.

Dispersion-corrected density functional theory (DFT)^361^ and molecular dynamics (MD)^316^ have been used to analyze the forces involved in the assembly of chitin chains.^362^ These usually predict the interactions (mainly H-bonding) within the chains and crystal domains they form upon assembly.^363^ MD simulation facilitates the understanding of the structure, dynamics, and energy characteristics of the assembly in aqueous media of chitin chains into chitin nanofibrils (Figure 20).^316^ Following Figure 20b1 and c, chitin nanofibrils were assumed to contain three chitin chains (1110), and nanofibril formation was driven by a synergy of weak nonspecific H-bonding during the early stages (100–150 ns) but eventually transformed into the (3000) system (150–400 ns), where the most dominant interaction upon stacking was specific H-bonding (Figure 20b2). This model revealed that, despite being nonspecific, H-bonds formed by atom pairs of O_6_–H_2_N and O_2_N–H_3_O were rare in crystalline chitin or assembled nanofibrils. Their presence influenced the efficiency of nanofibril assembly and highlighted the importance of molecular-level forces in controlling the assembly of chitin chains. MD simulation is useful in predicting the performance of nanochitin since it has been demonstrated that some of the inherent properties of individual chitin nanofibrils (e.g., mechanical strength) are governed by the modes of molecular packing rather than the cross-sectional dimension and crystallinity.^42^

**Figure 21:**
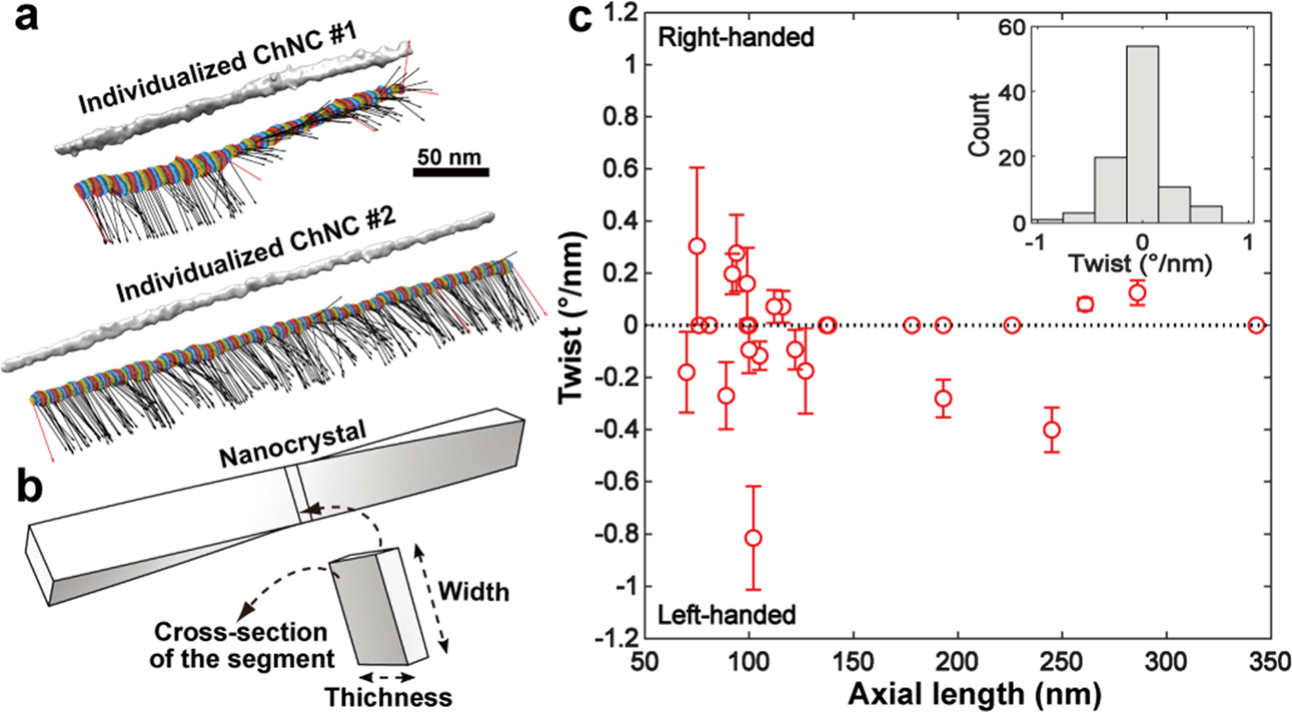
(a) Tomography reconstructions and respective chirality profiles obtained from Cryo-TEM images of a ChNC based on principal component analyses of nanocrystals showing left-handed (Individualized ChNC #1) and nonchiral (Individualized ChNC #2) twist profiles along the axial direction. (b) Schematic diagram showing a standard model used for analyzing the twisting features along the axial direction of the nanocrystal. (c) Chiral twist (±99% confidence interval) of ChNCs as a function of axial length (±standard deviation) of the cross-sectional segments in (b). The inset in (c) summarizes the number of ChNC samples with different twisting behavior. The total number of analyzed ChNC samples was 28. Adapted from ref (315). Copyright 2020 American Chemical Society.

A unique property of nanochitin that is revealed from MD simulation is its chirality, which is possibly induced by the H-bonding that occurs during assembly.^316^ The axial chirality was observed from nanofibril MD models (Figure 20a), particularly for the (2220) and (6000) systems, which was speculated to be a consequence of intrinsic axial chirality of single chitin chains and intermolecular interactions between the chains. During assembly, stacking of chains on top of each other, in the *a* direction, preserved their axial chirality. Meanwhile, the presence of additional nanofibrils in the *b* direction significantly decreased the chirality, which might be caused by a dipole–dipole interaction between the acetamide groups from adjacent stacked chitin layers, as well as the H-bonding distribution within the chains. This is in contrast to results of cryogenic transmission electron microscopy (cryo-TEM) and electron tomography together with corresponding 3D simulation (Figure 21a and b), which indicated that individual ChNC extracted from a never-dried chitin precursor exhibited limited chirality, with no obvious twisting preference (Figure 21c).^315^ This phenomena might be ascribed to the existence of H-bonding networks that form upon assembly, generated from the interactions of the hydroxyl and amino groups within chitin macromolecules and surrounding water molecules.^364^ The water molecules particularly competed with the interactions between macromolecules in the amorphous domains.^365^ Such effect upsets the natural tendency of chitin chains and prevents or limits development of chirality. The experimental results demonstrate the effect of water molecules and corresponding interactions in the development of the chirality of nanochitin. Additional efforts should consider more fundamental aspects associated with the thermodynamics of assembly under the framework of enthalpic and entropic effects.

#### 4.1.2 Colloidal-Scale Interactions

**Figure 22:**
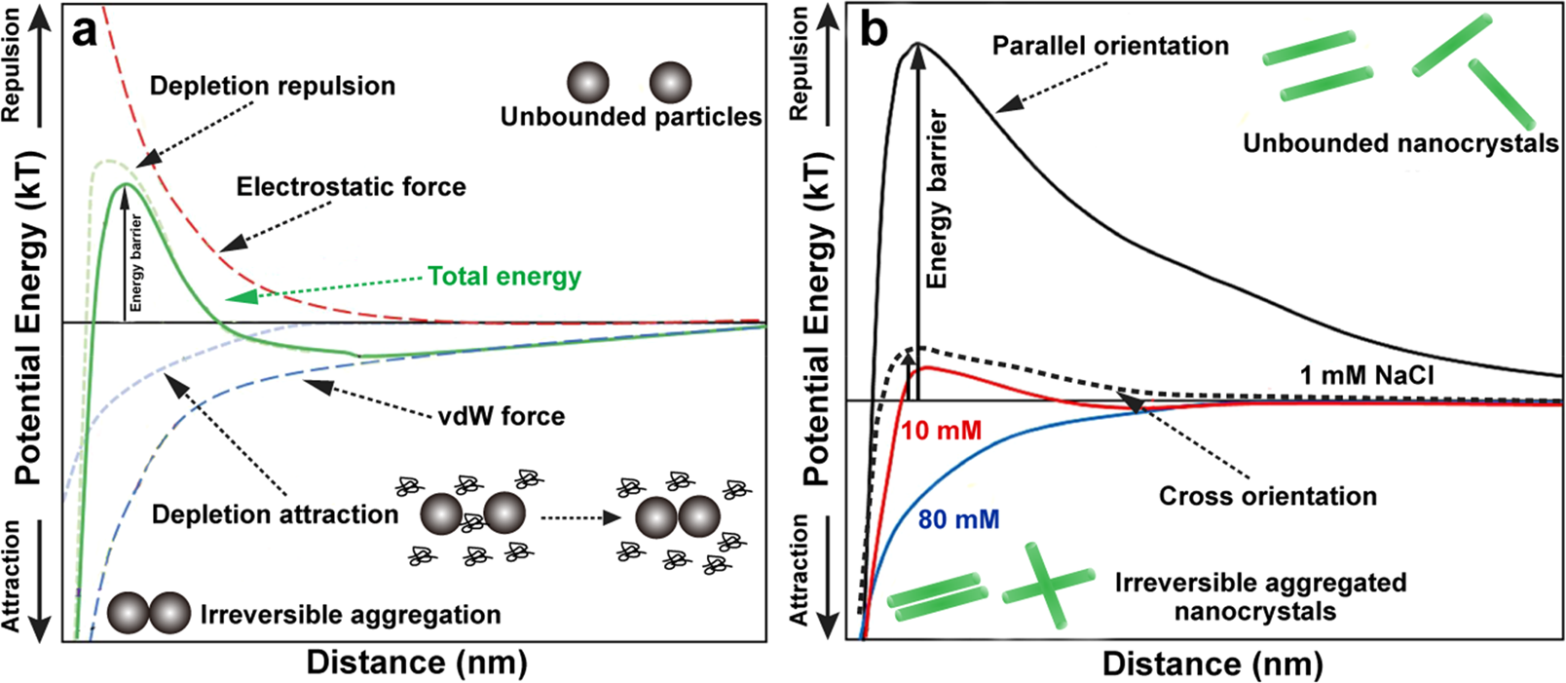
Schematic illustration of DLVO profiles of (a) spherical nanoparticles subjected to interactions at the colloidal scale, including van der Waals (vdW) forces, electrostatic, and depletion interactions and (b) rodlike ChNCs in aqueous suspensions of given ionic strength (1, 10, and 80 mM NaCl), considering parallel and cross orientations and under relevant forces operating at the colloidal scale (vdW and electrostatic). Adapted with permission from refs (366) and (370). Copyright 2020 and 2017, Elsevier.

Theoretically, assembly at colloidal scales can be described in the framework of net energy potential or forces in the system, unifying the molecular scale interactions. In principle, the interparticle colloidal interactions guiding particle assembly can be estimated considering vdW and electrostatic forces acting on the particles, for instance, as a function of interparticle separation distances.^366^ Accordingly, the net effect is the result of net attractive or repulsive forces that can be analyzed by the Derjaguin–Landau–Verwey–Overbeek (DLVO) theory (Figure 22a),^367^ together with considerations on the geometrical and surface properties of the particles. This enables a prediction of the dispersion behavior, for instance, colloidal assembly and stability. While DLVO theory is often applied to spherical (nano)particles, it can be extended to describe assembly of rodlike nanochitin at the colloidal scale.^368^

The shape of ChNC in a dispersion can be assumed to be either a cylindrical rod or straight parallelepiped. Taking such morphological characteristics into account, a rodlike ChNC experiences different configurations during the assembly at the colloidal scale. Among them, two ideal conditions include parallel and cross orientations that define the boundaries for net potential energy during ChNC assembly. In most ChNC suspensions, counterions are brought into the systems during preparation, generating additional effects on nanocrystal assembly, for instance, reducing the length of the electric double layer (EDL). Combining these factors, the profiles of net potential energy (the sum of vdW and electrostatic forces) acting on the assembly of ChNCs under different environmental conditions (*e.g*., variation of the salinity) for both parallel and cross orientations can be obtained, as depicted in Figure 22b. At very short distances, the vdW attractive force dominates over electrostatic (repulsion) interactions, thereby resulting in irreversible nanocrystal aggregates. In this range, even the thermal energy of nanocrystals, Brownian motion, is not sufficient to overcome the vdW attractive force.^369^ At intermediate separation, the repulsive electrostatic force dominates in the system, which prevents the nanocrystals from aggregating by overlapping EDL of each nanocrystal. Finally, at large separation distances (far from each other), nanocrystals do not interact except for very weak long-ranged vdW forces (Figure 22a).

Considering the two ideal configurations in the assembly of ChNC, repulsive energy barriers are much higher in the case of parallel orientation compared to those in cross-orientation at similar environmental conditions (Figure 22b). This is because orientation plays a significant role in defining the net energy barrier as a consequence of the interactions between ChNCs.^370,371^ In practice, ChNCs hardly arrange in suspension under the ideal configurations, parallel or cross orientations, and the actual net potential energy takes a value in between. Changing the salinity of the surrounding aqueous environment for ChNCs in a parallel configuration significantly affects their assembly (Figure 22b). More specifically, increasing salinity would reduce the effective nanocrystal surface charge, resulting in the compression of the EDL, which reduces the repulsive forces.^372^ At a slight increase in salinity (red line, Figure 22b), the repulsive energy barrier sharply decreases but still remains repulsive for a wide range of distances. In the meantime, a secondary minimum for net potential energy exists, indicating the occurrence of weak nanocrystal aggregation. Such an effect is caused by the weak net attraction forces, so that interparticle bridging is easily broken under certain external perturbances. Increasing the salinity (blue line, Figure 22b), the potential energy becomes attractive, which results in instant aggregation of nanocrystals due to the formation of strong interparticle vdW attraction. In sum, the interaction forces that operate at the colloidal scale during nanochitin assembly are relatively sensitive to the variation in the surrounding medium.^373^ The DLVO profiles are useful in understanding the net interactions between the particles, but one should be aware of the many factors that are at play,^374^ including dielectric constant, valence of the ions, ionic strength, and temperature. Moreover, apart from the vdW and electrostatic forces, other interactions control particle assembly, for instance, depletion and nonspecific hydrophobic forces, both of which are relevant to polysaccharides nanoparticles.^273,375,376^ In particular, depletion forces can be integrated into DLVO theory (Figure 22a). When nanoparticles are codispersed with non-adsorbing substances, they may experience depletion forces that originate from excluded volume effects.^377^ Theoretical calculations suggest that both short-ranged depletion attraction and long-ranged depletion repulsion can take place during colloidal assembly,^378^ depending on the distance between particles and dimension of the non-adsorbed substances. For instance, depletion forces originating from protein molecules induce interparticle attraction between ChNCs, promoting *in situ* assembly into segmented gelation domains in the dispersion.^379^ In sum, colloidal-scale forces enable various nanochitin assemblies; however, the association between chitin nanoparticles and final properties of the assembled structures requires more detailed studies.

#### 4.1.3 Micro- and Macroscale Interactions

**Figure 23:**
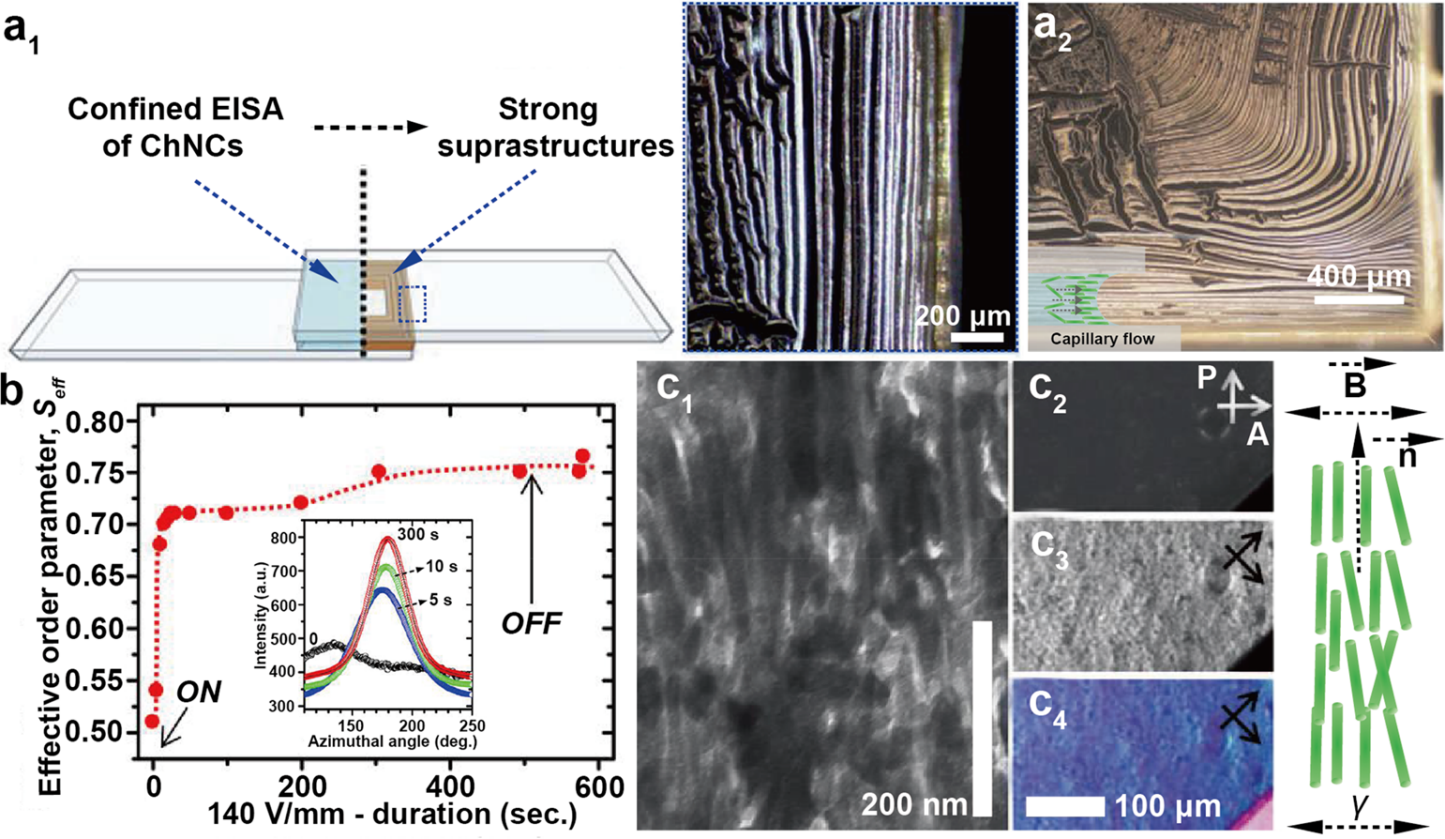
(a1) Schematic representation of the bonds formed from ChNC suspension via confined evaporation-induced self-assembly (EISA). The dashed box indicates the long-range order of the lamellar structure in (a1), image obtained by polarized optical microscopy (POM). (a2) Schematics showing a POM image of the lamellae formation and distribution of ChNCs along the bond under capillary flow. Adapted with permission from ref (384). Copyright 2021 The Royal Society of Chemistry. (b) Order parameter *S*_eff_ of ChNC/siloxane suspension inferred from *I* = *f*(ψ) traces as a function of duration of the applied electric field. The inset corresponds to azimuthal intensity profiles *I* = *f*(ψ) at a different time intervals after applying the field and revealed (SAXS) a gradual alignment of ChNCs. Adapted from ref (395). Copyright 2013 American Chemical Society. (c1) TEM micrograph of a mesoporous ChNC/siloxane nanocomposites with aligned pores induced by self-assembled ChNCs. (c2–c4) Series of representative polarized-light micrographs of the composite in (c1) for different orientations with respect to the directions of both the magnetic field **B** and the polarizers (P for polarizer and A for analyzer). Homogenous birefringence of uniaxially oriented sample is revealed by a 4-fold increase in light intensity after a 45° rotation (c2 to c3). (c4) Blue color obtained after introducing a first-order λ retardation plate (γ = slow axis direction), indicating the aligned structure along ***n*** perpendicular to the direction of the magnetic field **B**. Adapted with permission from ref (397). Copyright 2010 John Wiley and Sons.

Assembly forces at the micrometric and macrometric scales closely depend on molecular- and colloidal-scaled effects. Through interactions at larger scales, the most intriguing phenomenon involving nanochitin assembly is the formation and optimization of ordered alignment. A range of possible forces that are relevant to nanochitin systems will be introduced in this subsection. Capillary force is considered to be one of the strongest forces in a wide range of scale levels.^380,381^ They are mainly responsible for the formation of assemblies upon drying, which are held together in such a way that the interactions surpass the interfacial potential between particles and the substrate, resulting in the assembly of macroscopic materials.^382^ In the framework of the Laplace–Young equation, the capillary force can be enhanced by increasing wettability or confinement.^383^ Taking advantage of the capillary force in a confined area, evaporation-induced self-assembly (EISA) of ChNCs was achieved, generating strong ChNC superstructures with long-range ordering (Figure 23a1). Within such unique structure, a birefringent ChNC lamellae was formed, with ChNCs being aligned parallel to the lamellae orientation, as observed by polarized optical microscopy (POM) (Figure 23a1).^384^ Upon EISA, capillary flow-induced alignment of ChNCs in suspension was considered to be the main mechanism responsible for the accumulation of nanocrystals near the three-phase contact line (Figure 23a2).

Shear enables flow-induced particle alignment at the micrometric and macrometric scales.^385^ While shear is complex and dynamic, e.g., interfacial shear and internal flow simultaneously occurs and it indeed enhances long-range ordering of particles or particle mixtures. This particularly applies to rodlike nanochitin, where shear-flow alignment is more efficient for anisotropic systems,^386^ maximizing the shape effect in directing the assembly. For example, the cholesteric nature of shear-aligned ChNCs was identified using small-angle X-ray scattering (SAXS).^387^ Moreover, a layer containing well-ordered ChNCs can be vertically coated onto a polylactide film through the action of shearing force with the assistance of a polydopamine interlayer.^388^ In some cases, the structural and chemical features of nanochitin, e.g., longitudinal and transversal elastic moduli, shape responsiveness,^389^ and its interaction with surrounding viscoelastic environment,^390^ are also key for the assembly under shear. For instance, unidirectional shearing induced alignment of the mesophase in ChNC and AA mixtures, wherein aligned ChNCs were well retained in solidified ChNC/PAA composites.^391^ Finally, nanochitin suspensions display a shear-thinning behavior,^356^ and it becomes more pronounced with increasing nanochitin concentration, particularly for long ChNF.

Besides the assembly forces directly imposed to particles, noncontact forces generated from magnetic or electrical fields can control particle assembly at the micrometric and macrometric scales.^387,392−394^ The application of such forces relevant to the assembly depends on the electric and magnetic dipole moment, which relates to the intrinsic dielectric properties and anisometric nature of the given particles as well as the surrounding environment. For example, particles can be polarized under an electric field due to the accumulation of surface charges, which results in dipole orientation in the long axis of the particles along the field.^395^ The orientation of ChNCs along the direction of the electric field in a ChNC/siloxane suspension was revealed by SAXS, wherein the effective nematic order parameter, *S*_eff_, rapidly increased from 0.50 (without electric field) to 0.75 (under electric field), resulting in almost defect-free nematic single domains of ChNCs (Figure 23b).^395^ Moreover, extending the duration of the electric field, the azimuthal intensity profiles of the suspension gradually exhibited stronger peaks, located at an angle (180°) corresponding to the direction perpendicular to the electric field, producing an enhanced ChNC alignment (Figure 23b, insert). A similar orientation effect in nanochitin suspension is achieved under magnetic fields.^396^ For instance, under a magnetic field (9 T), the ChNC/siloxane suspension resulted in a composite suspension with homogeneous long-range alignment of chitin nanorods perpendicular to the magnetic field, increasing the light intensity for homogeneous birefringence (Figure 23c).^397^ In sum, the micro- and macroscaled assembly forces, either intrinsic or external, influence the formation and control of nanochitin or its composites into various suprastructures and their order and alignment in large-scaled systems.

### 4.2 Assembly of Nanochitin at Interfaces

During materials design and manufacture, especially involving nanostructured hybrids, the interface (air and liquid, liquid and liquid, or solid and liquid) is of utmost significance to the structure and performance of the obtained materials. This particularly applies to materials derived from nanochitin due to its colloidal nature and inherent hydrophilicity. By exploiting multiscale assembly forces, nanochitin has a great capability to assemble under different conditions to form given suprastructures. More importantly, during assembly at multiple interfaces, nanochitin presents a percolation threshold at low volume fractions.^398,399^ Nevertheless, uncontrolled nanochitin assembly via adsorption or adaptation at different interfaces challenges the process of manufacturing and the development of desired functionalities. Thus, appropriate nanochitin wettability, which dictates the contact, assembly, adaption, and arrangement at different interfaces, is a prerequisite for any material application.^400,401^ While the behavior of isotropic particles (e.g., spheres) at interfaces has been extensively investigated, that for anisotropic fibril-like nanochitin, with its high aspect ratio and cationic surface, is yet to be fully understood. In this subsection, the assembly of nanochitin at different interfaces and relevant physicochemical phenomena will be reviewed while keeping in context the potential of nanochitin as a building block for material fabrication.

#### 4.2.1 Nanochitin at Liquid/Liquid Interfaces

**Figure 24:**
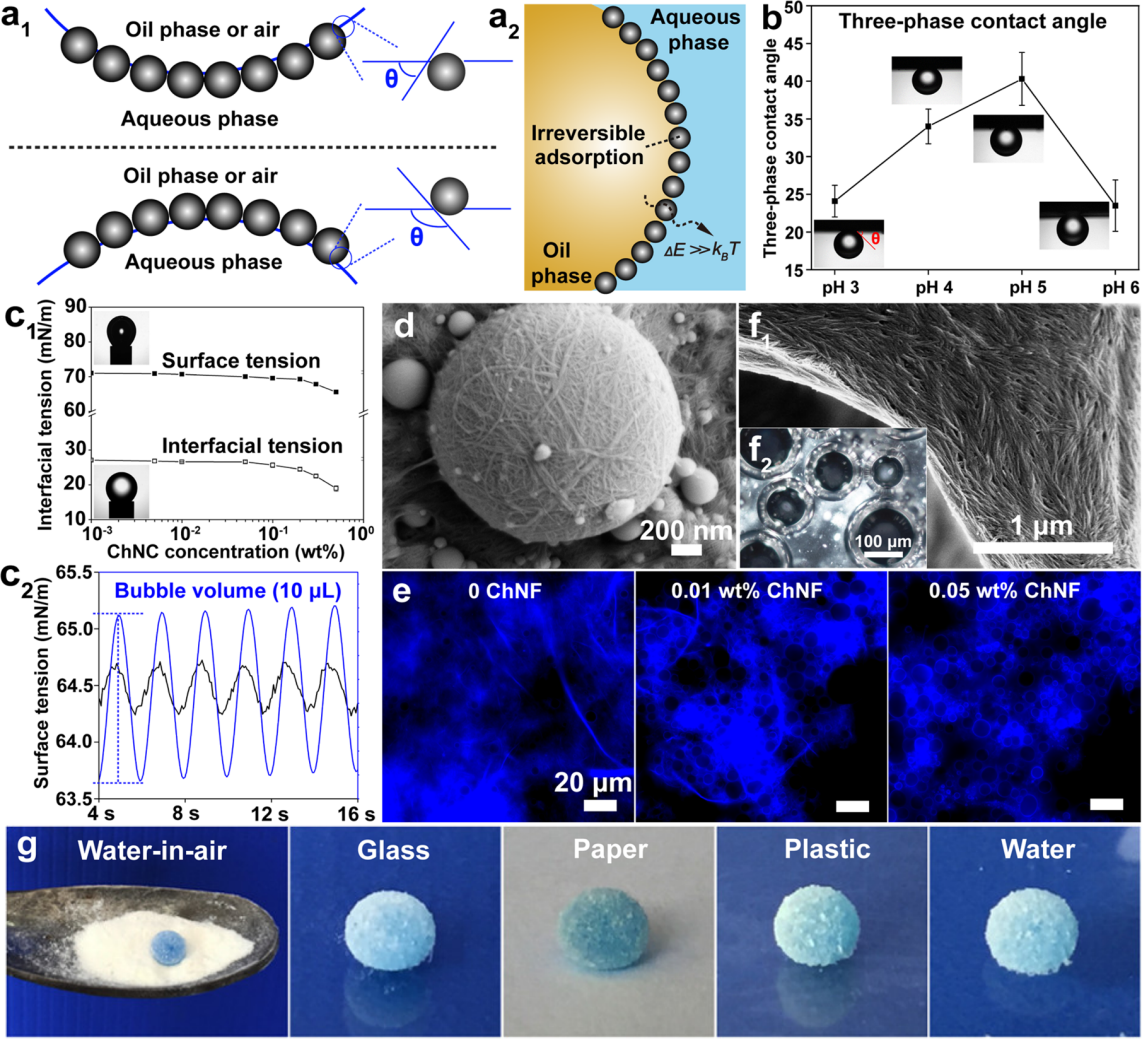
(a1) Schematic illustration of the adsorption of spherical particles at the oil/water interface according to the contact angles (θ). Adapted with permission from ref (407). Copyright 2002 Elsevier. (a2) Adsorption of particles with a contact angle < 90° in an oil-in-water (O/W) system. The desorption of the particles from the interfaces is prevented if the energy barrier for detachment is higher than the thermal energy. Adapted from ref (428). Copyright 2021 American Chemical Society. (b) Three-phase contact angle of ChNF films spin-coated from ChNF suspensions of varying pH (3–6). θ was measured by injecting a sunflower oil droplet on a solid ChNF film in contact with water. Adapted from ref (410). Copyright 2020 American Chemical Society. (c1) Surface (air/water) and interfacial (oil/water) tension of ChNF suspensions at given concentrations. The inset shows the shape of the bubble or droplet during measurement. (c2) Interfacial dilatational rheology of air/water interfaces using a 10 μL bubble oscillating at 0.5 Hz frequency, demonstrating the interfacial adsorption of ChNFs in a 0.5 wt % suspension. Adapted from ref (273). Copyright 2019 American Chemical Society. (d) SEM image of ChNF-stabilized polystyrene sphere showing a high ChNF surface coverage. Adapted from ref (417). Copyright 2021 American Chemical Society. (e) Fluorescent images of ChNF/CNF-stabilized sunflower O/W Pickering droplets at different ChNF concentrations. CNF/NCh complexes were stained using Calcofluor white. Adapted from ref (417). Copyright 2021 American Chemical Society. (f1) SEM image of a ChNC-stabilized Pickering foam after drying. (f2) Polarized optical micrograph of a ChNC-based foam in liquid state. Adapted with permission from ref (425). Copyright 2015 The Royal Society of Chemistry. (g) Images of water-in-air liquid marbles (10 μL of water, dyed with methylene blue) wrapped by superamphiphobic ChNC powder on the surfaces of glass, paper, plastic, and water. Adapted from ref (339). Copyright 2020 American Chemical Society.

Emulsion forms by process of mixing two immiscible liquids, with one of the liquids being dispersed in the other, the continuous phase.^402^ Since emulsions are thermodynamically nonequilibrium systems, surface-active emulsifiers are required to reduce the interfacial tension between the immiscible phases, preventing or delaying macro-phase separation, eventually enabling kinetical stability.^403^ Over a century ago, Ramsden^404^ and Pickering^405^ demonstrated that colloidal particles assemble at liquid/liquid interfaces to form and stabilize multiphase systems, referred to as Pickering stabilization. The interfacial assembly of certain type of colloidal particles depends on their wettability with respect to each phase,^406^ resulting in oil-in-water (O/W, θ < 90°) or water-in-oil (W/O, θ > 90°) emulsions when the colloidal particles are preferentially wetted by water or oil, respectively (Figure 24a1). Based on Binks’ postulates,^407^ the interfacial adsorption of spherical particles of proper size and wettability is considered to be irreversible, given the large energy or barrier required for particle (Δ*E*) from the interface compared to the thermal energy (*k*_B_*T*) (Figure 24a2). Nanochitin is anisotropic and rodlike, which results in the added consideration of particle shape as far as interfacial orientation and packing during Pickering stabilization,^408^ In such a case, Δ*E* can be calculated according to^409^
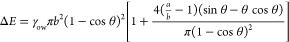
where α and *b* are dimensions of long and short semiaxes, respectively, γ_ow_ is the interfacial tension between oil and water phases, and θ is the equilibrium three-phase contact angle reflecting the partial wetting of the particles by the two fluids. Comparing the cross-sectional areas at the interface for rodlike and spherical particles, the inequality condition Δ*E*_rod_ > Δ*E*_sphere_ applies for any value of the contact angle between 0 and 180°.^409^ This suggests that nanochitin generates an even stronger physical adsorption and more easily assembles at the liquid interface compared to the equivalent spherical particles. Thus, nanochitin assembled at the interface via the Pickering mechanism makes the system become extremely stable.

Owing to the hydrophobic acetyl and hydrophilic amine groups on the surface of nanochitin, its wettability with respect to the oil and water phases can be balanced by adjusting the number density of these groups,^369^ facilitating the interfacial adsorption and assembly. Unfortunately, there are no straightforward means to achieve a precise control of nanochitin surface chemistry. Experimentally, by adjusting the pH of ChNF suspensions, the contact angle of ChNF films can be slightly increased with an increase in pH (i.e., via deprotonation) in a three-phase contact angle test (Figure 24b). Despite such a fact, ChNF films are hydrophilic with a contact angle < 90° at the tested pH range.^410^ This indicates the possibility of controlling the surface chemistry of nanochitin to change the wettability at the oil/water interfaces.^411^ The inherent hydrophilicity of unmodified nanochitin, favoring affinity with the aqueous phase,^412^ indicates the preferred formation of O/W emulsions (Figure 24a). In contrast to molecular-based emulsifiers, which exhibit fast adsorption/desorption kinetics, nanochitin does not adsorb at the interfaces spontaneously at low concentrations, given its high hydrophilicity and dimensions (Figure 24c1).^273^ With increasing the loading levels, the surface/interfacial tension of ChNF suspensions slightly decreases (Figure 24c1). More clearly, dilatational tests show the periodic change in surface tension of ChNF suspensions with a synchronous change in bubble volume during oscillation (Figure 24c2), demonstrating the adsorption and assembly of ChNFs at the interfaces. This observation implies that a high energy input is required to facilitate the interfacial assembly of nanochitin.^413^ In most of the cases, ultrasonication or homogenization is used to promote the adsorption of nanochitin at the oil/water interfaces.^414^ On the other hand, the enhanced interaction between nanochitin and the dispersed phase facilitates its adsorption.^415^ For instance, if the lipids contain hydroxyl and epoxy groups, the interactions between such chemical groups in the oil droplets and the amine and hydroxyl groups of nanochitin are strengthened through the effect of affinity with the polar groups, promoting interfacial adsorption, assembly, and rearrangement of nanochitin on the oil droplet.^416^

In a typical Pickering stabilization with ChNF *via* ultrasonication, the fibrils assemble tightly on the droplets, forming a dense, randomly-distributed network (Figure 24d).^417^ While nanochitin has high surface charge, the inhomogeneous distribution of cationic groups results in a weak interparticle repulsion, so that the charges are screened prior to interfacial adsorption. This phenomenon does not apply to case of nanocelluloses.^376^ The rodlike, anisotropic features enable the possible orientation and rearrangement of chitin nanorods at the liquid/liquid interfaces, which is not possible for (isotropic) spheres;^418^ thus, nanochitin at a low concentration is sufficient to stabilize the interfaces.^419^ Moreover, the aspect ratio of nanochitin determines its assembly at the liquid/liquid interfaces.^420^ For instance, it has been reported that an ultralow concentration of ChNF with low aspect ratio (0.005 wt %) is sufficient to successfully stabilize Pickering droplets of several micrometers in size but with a surprisingly low total surface coverage.^273^ By increasing the ChNF concentration, the surface coverage of the droplets is significantly improved, resulting in smaller droplet size. These results demonstrate an excellent capability of ChNF to assemble at the interface in a wide range of concentrations, much better than any reported biobased nanoparticle, including nanocelluloses.^376^ At a high concentration (0.3 wt %), ChNF of high aspect ratio still achieve a lower surface coverage but form an interconnected network among droplets and in the continuous phase, stabilizing the droplets.^273^

Apart from its ability to assemble at liquid/liquid interfaces, nanochitin coassembles with other biomass-derived structures. For instance, a higher interfacial elasticity is measured for a soy oil droplet in aqueous suspension (pH 3) in the presence of ChNC and a surface-active protein (β-lactoglobulin) compared to that for the system stabilized with either of the single components.^421^ Another route to coassembly is based on the cationic character of nanochitin, which forms electrostatic complexation with anionic substances. The combination of hydrophilic and hydrophobic acetyl groups enables an enhanced adsorption of the complexes at the interfaces;^422^ for instance, this is the case of a combination of ChNF with anionic CNF.^376,417^ More importantly, dynamic interfacial coassembly of ChNF/CNF can be tuned by the concentration of CNF freely dispersed in the aqueous phase, going from low ChNF to full CNF/ChNF surface coverage (Figure 24e). This approach can be used to improve the interfacial adsorption efficiency of biocolloids that have a lower tendency for interfacial adsorption.

#### 4.2.2 Nanochitin at Air/Liquid Interfaces

Particle assembly at the air/liquid interfaces can lead to the stabilization of air-in-water Pickering foams or water-in-air liquid marbles,^423,424^ depending on the wettability of the particles with respect to each phase (Figure 24a1). Long nanochitin shows limited amphiphilicity, a high aspect ratio, and no apparent surface activity (Figure 24c1). This challenges spontaneous adsorption or assembly at the air/water interfaces via Pickering mechanism. Native ChNCs stabilize liquid Pickering foams only if high-energy ultrasonication is applied to generate air bubbles and by partial screening of the positive surface charge of ChNC (e.g., by increasing the pH).^425^ Thus, under the conditions of weak interparticle electrostatic repulsion, nanocrystals form close-packed coverage at air/water interfaces and tend to form a parallel nematically oriented organization (Figure 24f1). This unique assembly of ChNCs present at the air/water interfaces was further revealed by POM (Figure 24f2), where clear, intense birefringent regions were identified at the bubble surface, indicating the presence of an ordered arrangement of ChNCs at the interfaces. Hence, rodlike chitin nanoparticles can orient locally at the air/water interfaces within the foam, which is useful to create lightweight materials with hierarchical structuring. The assembly of nanochitin at the air/water interfaces can be promoted by coadsorption of nanochitin and a surface-active surfactant,^426^ with the latter component acting as foam-forming agent. Meanwhile, nanochitin improves the integrity of the foam. The interfacial adsorption and assembly of anionic ChNC produced by TEMPO-mediated oxidation were promoted by enhancement of hydrophobicity through electrostatic complexation with cetyltrimethylammonium bromide, a cationic surfactant.^427^ Moreover, the role of surface charge and size to improve assembly at the air/water interfaces was demonstrated by changing the size of ChNC (varying TEMPO conditions). Hydrophobized ChNCs (using thiol groups and highly fluorinated long chains) stabilized the air/water interfaces in liquid marbles,^339^ which were stable when placed on various substrates, showing no signs of collapse (Figure 24g).

#### 4.2.3 Nanochitin at Solid/Liquid Interfaces

Besides nanochitin assembly at the liquid/liquid and air/liquid interfaces, that at solid/liquid interfaces is relevant to the formation of nanostructured coatings or surface patterns, which can be transferred to solid surfaces consisting of dried nanochitin. Long-range order of nanochitin coating formed at the solid/liquid interface can be enhanced by controlling the capillary forces or by deposition upon drying.^429^ For instance, spin-coating facilitates nanochitin assembly at solid surfaces via spreading a nanochitin suspension by centrifugal forces until the desired thickness is achieved.^430^ To this end, the properties of the substrate, e.g., surface chemistry, energy, roughness, etc., are critical to control the formation and assembly of nanochitin at the solid/liquid interfaces. ChNFs assembled selectively onto a solid cotton fabric modified by partial carboxymethylation, where the ionized anionic groups on the cotton fabric enabled a strong electrostatic affinity with ChNFs.^431^ This coating process produced uniform surface porous nanostructures with a randomly assembled network of ChNFs and created unique functions. Apart from the assembly on solid surfaces, nanochitin can coassemble with other components at solid/liquid interfaces, which brings additional attributes. In this regard, ChNCs coassembled with a water-based acrylic resin on a surface, forming a close-packed structure with biaxially oriented solid polypropylene film.^432^ As the composite coating consolidated, the modulus and surface energy of the material were enhanced while the optical transparency was maintained.

### 4.3 Assembly of Nanochitin in Water

Materials development based on the assembly of nanochitin at interfaces relies on the heterogeneity of the construction. Direct use of nanochitin dispersions, as a route for materials innovation, benefits from the properties encoded in chitin structures, such as anisotropy, guiding the assembly under appropriate interaction forces.^433^ One example is the formation of long-range ordered LCs in ChNC suspensions,^382^ mimicking the helicoidal Bouligand structures found in the biological tissues of the exoskeletons of crustaceans. Assembly of nanochitin is governed by the environment surrounding the nanoparticles and the components that coexist in the suspension.^379^ New advances in materials design will need a better understanding of the unique colloidal, geometrical, and spatial features that regulate nanochitin assembly as well as its interactions in water. External control of such factors at the nanoscale will allow the hierarchical assembly of nanochitin for novel biomimicry and the fabrication of functional nanochitin materials for emerging applications. In this subsection, we discuss the assembly of nanochitin in water as well as the forces and phenomena that affect the assembly.

#### 4.3.1 Self-Assembly of Chitin Nanocrystals in Liquid Crystalline Phases

**Figure 25:**
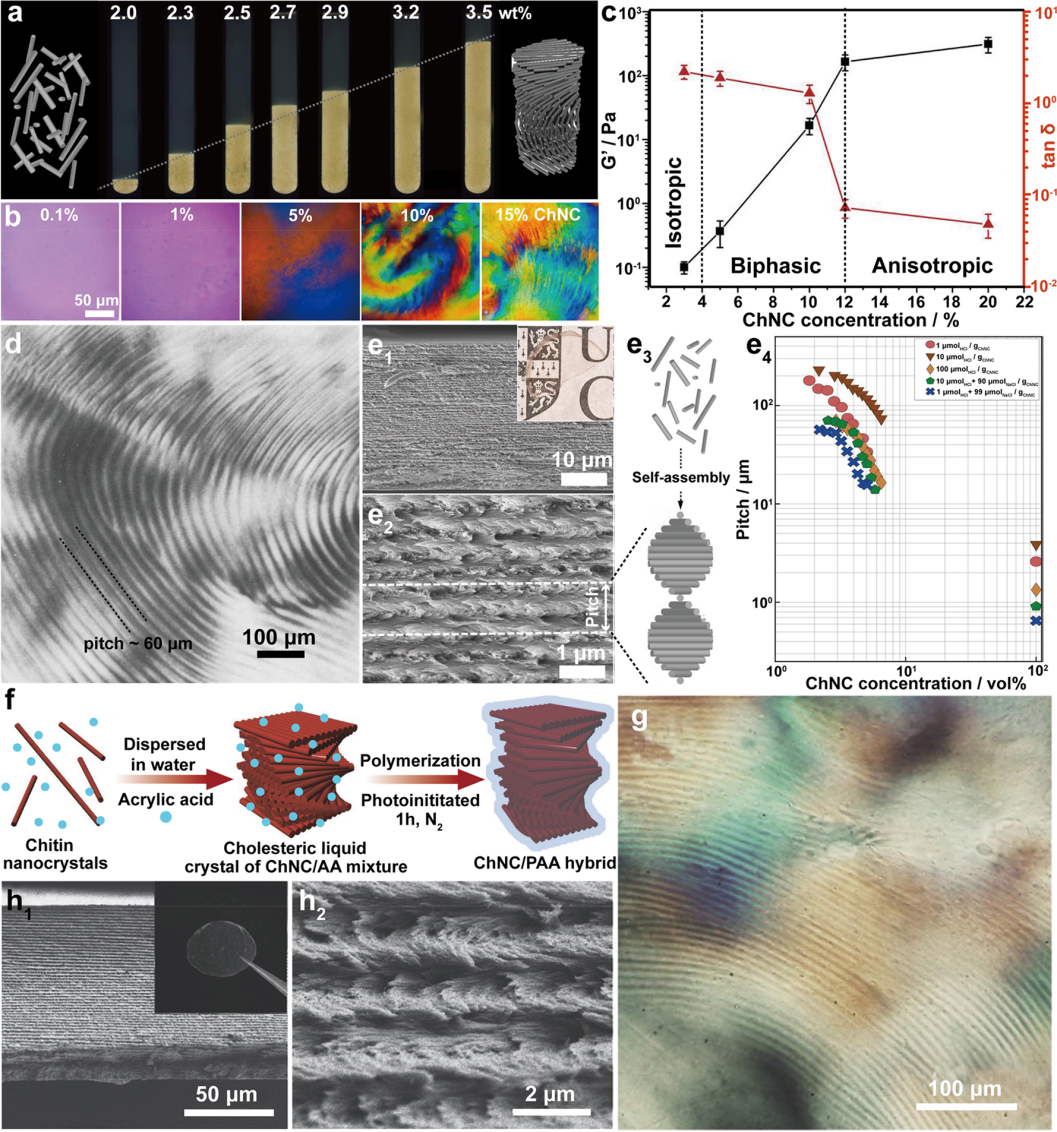
(a) Lyotropic liquid crystalline transitions of ChNC as a function of concentration, showing a dark, upper isotropic phase and a bright, bottom nematic phase. The left- and right-hand side schematics illustrate the disordered organization and ordered liquid crystals of ChNCs, respectively. Adapted with permission from ref (439). Copyright 2006 IOP Publishing. (b) POM images of ChNC dispersions of different concentrations as droplets suspended in silicone oil. Adapted from ref (442). Copyright 2018 American Chemical Society. (c) Concentration dependence of *G*′ and tan δ of ChNC suspensions with 0.1% strain at 20 °C. Adapted with permission from ref (443). Copyright 2019 Elsevier. (d) POM image of fingerprint pattern of the anisotropic phase formed from a ChNC suspension (5 wt %) stored for 1 day after treatment. The cholesteric texture exhibits periodic lines with spacing of ∼30 μm, corresponding to half of the cholesteric pitch. Adapted with permission from ref (438). Copyright 1993 Elsevier. SEM image of the cross section of the films prepared by drying a nematic ChNC suspension imaged at (e1) low and (e2) high magnifications, showing helicoidal architecture and Bouligand arches. The inset is a transparent film producing by evaporating the ChNC nematic phase. (e3) Schematic illustration showing the assembly of disordered ChNCs in suspension to ordered nematic organization, fitting the cholesteric pitch in d2. (e4) Chiral nematic pitch of ChNCs in suspension and in solid state at varied HCl and NaCl concentrations. Adapted from ref (304). Copyright 2019 American Chemical Society. (f) Illustration of the formation of a helically ordered ChNC/PAA hybrid via photopolymerization of ChNC/AA LCPs. (g) POM image of a fingerprint texture of ChNC/AA LCPs. SEM image of the cross section of cross-linked ChNC/PAA hybrid at (h1) low and (h2) high magnifications. The inset is the visual appearance of a typical composite film. Adapted with permission from ref (456). Copyright 2015 John Wiley and Sons.

The dynamic assembly in suspension occurs following the contribution of ChNC surface charge, anisotropy, rodlike shape, and conformability, together with the interparticle interactions. As a result, the most intriguing assembly of ChNC in a suspension is in the form of a two-phase equilibrium system, with isotropic and nematic liquid crystalline phases (LCPs),^434,435^ similar to the case of CNC.^436^ The long-range order corresponds to unidirectional alignment within loose planes, wherein each pseudoplane is partially rotated into a helicoidal arrangement along a principal axis. However, different from the self-assembly of CNCs into LCs,^26,27,437^ ChNC LCPs have been largely unexplored after the first report nearly 30 years ago.^438^ Colloidal ChNCs in suspension spontaneously self-organize into a nematic LCP after reaching a threshold concentration and undergo a transition from a disordered dispersion (isotropic phase) to a biphasic dispersion (coexistence of isotropic and anisotropic phases) and to a fully nematic suprastructure (completely anisotropic phase) (Figure 25a).^439^ This phenomenon follows the Onsager theory,^440,441^ where a nematic structure is induced following entropic effects, leading to a parallel alignment of anisotropic ChNCs. In principle, upon LCP transition, anisotropic ChNC droplets first form and sediment, given the higher density of the nematic phase, leading to droplet coalescence and to the formation of a continuous phase, until phase separation is complete. This results in a sharp interface separating the two coexisting phases.^439^ The isotropic-to-nematic phase transition in ChNC suspensions has been confirmed under polarized light, showing birefringence and local lamellar ordering of ChNCs at high concentration (Figure 25b).^442^ However, high concentration was required for ChNC to form nematic ordering and the transition occurred prior to kinetic arrest or gelation of the dispersion (Figure 25c).^443^ This is attributed to a thermodynamic self-adaptation of rigid nanocrystals in the suspension,^444^ wherein an increased interaction energy among nanocrystals occurs at increasing particle loadings, forming nematic-like ordered suprastructures, even at relatively low concentrations (5% in Figure 25c).^445^ Using POM, the anisotropic ChNC phase has been recognized by the characteristic fingerprint patterns that are formed and the nematic pitch, which corresponds to twice the periodicity of the measured fingerprint pattern and the well-ordered suprastructure of ChNCs, assembled in the suspension (Figure 25d).^438^

Based on theoretical analysis of the interactions acting on nanochitin (subsection 4.1), its self-assembly in suspension, to form a stable LCP, is found to be induced by a synergy of various colloidal assembly forces. This phenomenon is an entropy-driven process that has its origin in the anisotropic shape and mutual interactions between the charged, rodlike nanocrystals. Such a process starts by a nucleation and growth mechanism until a macroscopic phase separation occurs in the ChNC suspension.^304,438,446^ Factors such as pH, temperature, surface charge, and aspect ratio as well as electrostatic interaction exert an impact on the phase separation of ChNCs in suspension toward the formation of a LCP.^369,372,447−450^ Under the effect of increased ionic strength, a linear change of the volume fraction of the isotropic and anisotropic phases occurs.^387^ Moreover, the nematic pitch in ChNC suspensions varies with the addition of acid (concentration of HCl). In particular, when the ionic strength is over a certain level, phase separation of the bulk suspension ceases and, instead, small anisotropic (birefringent) ChNC droplets form, preventing coalescence or sedimentation. The change of surface charge of ChNC, from positive to negative via H_2_O_2_ hydrolysis of mechanically defibrillated chitin, produces a small impact on the formation of long-range nematic LC behavior of ChNC.^451^ Negatively charged ChNC exhibits a typical lyotropic LCP transition after reaching the critical concentration (Figure 25a). Uniquely, spontaneous self-assembly from a hierarchical 1D-nanofiber to a well-ordered 2D nanobelt and to a 3D multilayered lamellae occurs following a transition. The critical ChNC concentration does not obey the Khokhlov-Semenov and Odijk-Lekkerkerker theory of lyotropic phase transition of charged, rodlike colloidal dispersions,^452^ indicating the differences in theoretical and experimental results for ChNC assembly in LC systems.

For any application of hierarchically assembled ChNC LCPs, a uniform structure is a prerequisite to materialize novel functions. Typically, direct acid hydrolysis of chitin can be used to produce ChNC that forms nematic ordering;^438^ nevertheless, large-sized ChNC aggregates form and sediment in such systems, which influences the homogeneity of the suspension and results in a lack of control on self-assembly. Thus, selection of the extraction parameters and resultant nanocrystal properties are of critical relevance. As described previously, sequential treatment involving surface deacetylation and acid hydrolysis is a suitable strategy to improve the homogeneity of ChNC in suspension via increasing the positive surface charge density. Using this method, more uniform ChNCs were produced and capable of forming nematic LCs, which showed a strong birefringence given the formed optically anisotropic rodlike textures.^453^ More comprehensively, fine-tuning the colloidal properties of ChNC by adjusting the hydrolysis conditions and other treatments enables precise control over the LC behavior in suspension.^304^ For instance, decreasing the ChNC concentration in an aqueous dispersion lowered the LC pitch; meanwhile, higher surface charges led to an increased LC pitch, up to a maximum of 250 mm. An elegant strategy to explore the self-assembly of ChNCs in suspension is to follow the consolidation of the ChNC LCP suspension into solid films. Upon evaporation, the LCP of a ChNC suspension yields a solid, transparent film (inset, Figure 25e1). The nematic ordering of the self-assembled ChNCs can be retained in the dry film, forming a well-defined helicoidal nanoarchitecture (Figure 25e1 and e2). In this structure, ChNCs were locally aligned along a direction that spatially rotated in a left-handed fashion about an axis (Figure 25e3). Basically, the pitch of the helicoidal structure in dry ChNC films was relevant to the self-assembly of ChNCs in the nematic phase and the distortion experienced upon further drying of the ChNC suspension after the onset of kinetic arrest.^454,455^ The ChNC suspension turns from liquidlike to solidlike at a given concentration,^372,443^ which prevents nanocrystals from rearranging collectively.^449^ Upon evaporation during the onset of kinetic arrest, a vertical compression of the suspension occurs, which forces nanocrystals to become closer to each other, thus drastically reducing the pitch (Figure 25e2). Since both the ionic strength and pH influence the pitch prior to kinetic arrest and the onset of kinetic arrest itself, they are two main parameters that determine the pitch of the final helicoidal film. Thus, through a combination of pH and ionic strength, ChNC films with a well-ordered helicoidal arrangement and pitch values were achieved, spanning the range from 650 to 3720 nm (Figure 25e4). Thus, it is possible to unlock the potential of ChNC to construct novel materials bearing adaptive LCPs.

So far, we have discussed the self-assembly of ChNC into long-range ordered LCPs, as well as the transition of ChNC LCPs into homogeneous, smooth, and transparent dry films with preserved helicoidally layered structures.^457^ However, no chiral twisting within the nematic structures of the ChNC system has yet been reported. Thus, the desired attribute of photonic films assembled from ChNC is yet to be realized which might be a result of a possible lack of chirality (handedness) of chitin nanorods, as shown in Figure 21, subsection 4.1.1. Fine-tuning the helicoidal organization of nanocrystals and corresponding pitch values would lead to a tunable structural iridescence associated with chiral nematic order.^304^ Grafting monomethoxy PEG onto ChNC to trigger their chirality has been attempted to enhance chiral nematic organization.^351^ Unfortunately, no chiral structure was identified even with improved long-range order of the anisotropic mesogens. The failure to form chiral nematic phases toward structural color in dry ChNC films may also be ascribed to the natural variations among chitin sources and extraction methods.^434^ As a result, future investigation of LC self-assembly of ChNC may be focused on the chiral twisting of the chitin nanorods.

#### 4.3.2 Coassembly of Chitin Nanocrystals in Liquid Crystalline Phases

The formation of nematic LCs from ChNC has considered its coassembly with suitable components into the LCPs. Early studies have demonstrated that when certain amount of nonrodlike materials (e.g., silica precursors) is loaded into a ChNC suspension, reaching its threshold concentration, coassembly of ChNCs and the components present in the suspension can lead to nematic ordering.^397,453,458^ Remarkably, the heterogeneous components showed little effect on ChNC LCPs, demonstrating the robustness of the nematic organization of assembled ChNCs. For instance, a mixture of ChNC and chitosan coassembled in suspension formed different nanoscale structures, depending on the initial ChNC concentration.^435^ When chitosan was loaded into nematic ChNC LCPs, the nematic order of the ChNCs was retained, which could be further transferred to the solid chitosan matrix upon drying. Synthetic materials have also been used for coassembly, for instance, spontaneous coassembly of concentrated ChNC and an organic monomer, AA, led to cholesteric LCPs (Figure 25f), as revealed by the observed fingerprint texture of the formed ChNC/AA suspension (Figure 25g).^456^ More importantly, after photopolymerization of the ChNC/AA mixture (Figure 25f), cross-sectional SEM of the cross-linked structure confirmed an in situ cholesteric LC with a preserved helical order frozen inside the ChNC/PAA composite (Figure 25h). The stacked nanocrystals were found to adapt their orientation helically inside the composite, where the helical axis was oriented perpendicular to the surfaces (Figure 25h2). This result clearly indicates the opportunity to develop versatile photonic materials by combining LCPs of ChNC and functionalities of auxiliary ingredients in a coassembled system.

In sum, despite some limitations in the formation of LCs in a ChNC system, there are interesting prospects for the fabrication of multiscale and hierarchical architectures by controlling the structural complexity or templating periodically ordered suprastructures of ChNC. Further understanding of the means to control LC behavior of ChNC and codispersed components in suspension is pivotal in related endeavors.

#### 4.3.3 Other Forms of Nanochitin Assembly in Water

As described above, long-range ordered LC assembly of ChNC in water occurs at high solid concentrations. The assembly of nanochitin dispersion in a diluted system is influenced by long-range forces but in nonordered mode. Moreover, the interactions of nanochitin controlled by colloidal-scale assembly forces are more sensitive to the surrounding aqueous environments at low solid concentrations, given the relatively large interparticle distance and access to surface ionic charges,^369^ as introduced in Figure 22b. Such tunable colloidal properties of nanochitin make its assembly dynamic. For instance, the thermodynamic incompatibility of rodlike ChNC and globular whey protein isolate in a mixed suspension in acidic condition led to a gel-like, heterogeneous assembly containing ChNC-rich and ChNC-poor domains.^379^ This separation assembly was controlled by depletion attraction acting between ChNCs due to the imbalanced osmotic pressure induced by exclusion of globular protein biopolymers from the interspace between ChNCs. This is in line with theoretical analysis of the colloidal assembly forces.

**Figure 26:**
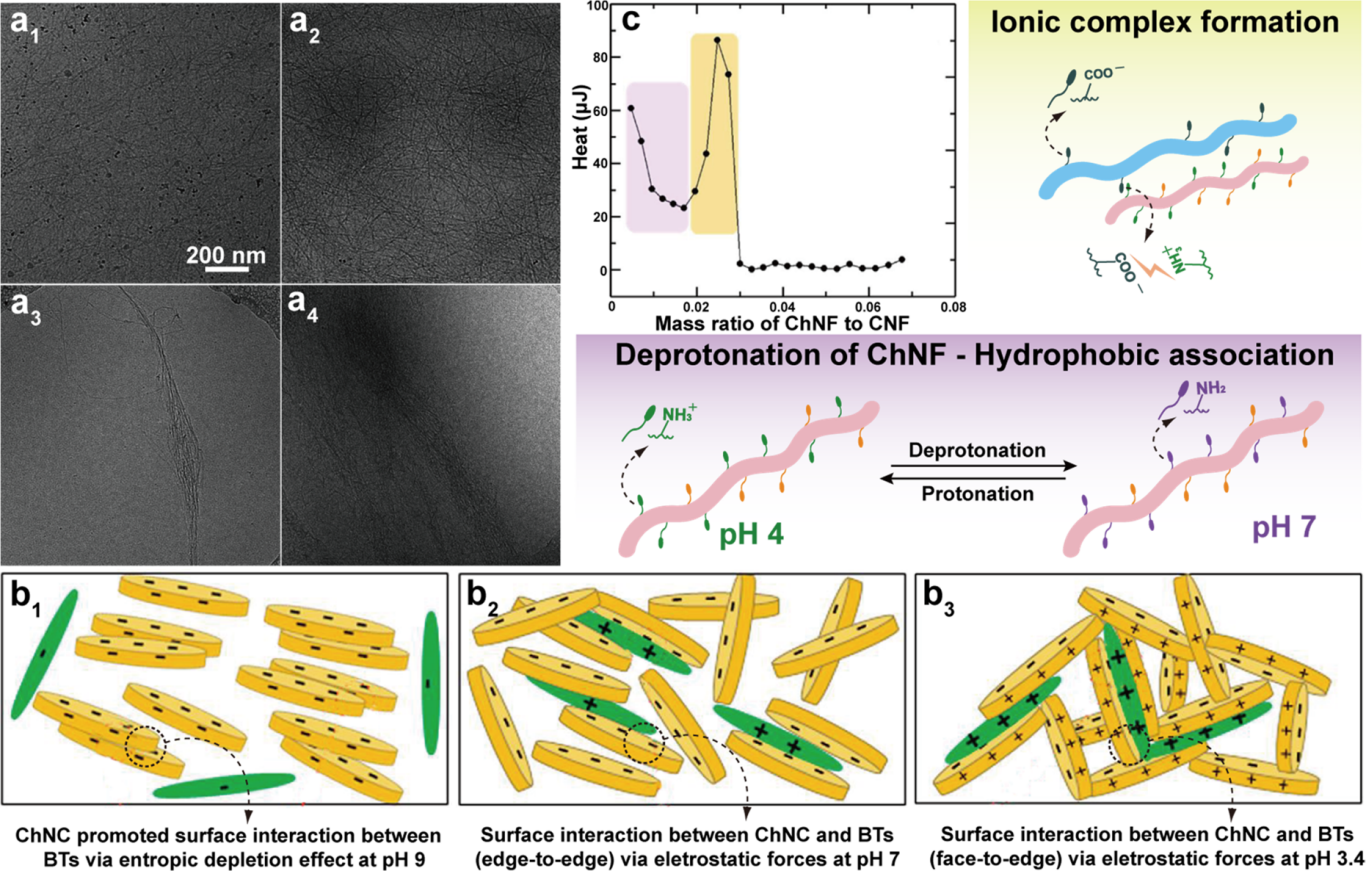
(a1–a4) Cryo-TEM images of β-ChNFs that underwent self-assembly at pH 8 in four different stages (followed by time of observation). (a1) is the start of the assembly, while (a2) to (a4) occurred after 30 s. The visualization of the self-assembly after longer time periods was not possible given the increased fiber thickness that prevented electron transmission. Adapted rom ref (460). Copyright 2019 American Chemical Society. (b) Schematic illustration showing the dispersion state and nanostructured surface interactions of ChNC and bentonite under alkaline (pH 9), neutral (pH 7), and acidic (pH 3.4) conditions. The assembly of ChNC can be tuned by the conformation of bentonite at different pH values. Adapted from ref (462). Copyright 2018 American Chemical Society. (c) Magnitude of heat signal and schematics of interactions for ChNF and CNF under different pH conditions. The heat signal upon deprotonation decreases as the pH is reduced, and equilibrium shifts to favor the protonated form of NCh (purple area). The pH is adjusted due to the high acidity of the ChNF suspension. The heat signal for ionic complex formation initially increases as the ionization of ChNF is increased, and the driving force for complexation improves (yellow area); meanwhile, the heat signal subsequently decreases as the charge groups on CNF are consumed. Adapted with permission from ref (463). Copyright 2021 Elsevier.

Changing the pH is a direct route to adjust surface functions and the state of the dispersion of nanochitin in water.^369,410,459^ When nanochitin surface charge is screened at high pH, colloidal-scale forces, e.g., hydrophobic interaction, dictate the assembly of nanochitin. For instance, precise control over nanochitin assembly in suspension in a less-charged surface condition resulted in hierarchically structured chitin microfibers or fiber bundles.^460^ In this process, β-ChNFs were used since they have a more open structure and weaker H-bonding network.^461^ Microscopically, despite their nonspecific interactions, β-ChNFs showed a propensity to aggregate in large 1D microfibers through laterally packing to micrometric loosely organized bundles (Figure 26a). Such self-assembly was triggered by varying the pH of the β-ChNF suspension and favoring the previously grown fibers. The kinetics of this process was regulated by electrostatic repulsion acting on the highly charged β-ChNFs. Coassembly of nanochitin and oppositely charged materials in the suspension can also lead to the formation of organized structures, depending on the pH. For example, interactions between oppositely charged ChNC and bentonite in the dispersion formed nanostructured assemblies at different pH values owing to the reversible protonation and deprotonation of ChNC and bentonite (Figure 26b).^462^ The coassembly in the suspension of ChNF and CNF altered the electrophoresis and dispersion state of the ChNF. Nevertheless, the optical turbidity of the complex suspension remained almost unchanged, implying a unique assembly behavior.^417^ Furthermore, a complex assembly process involving ChNF and CNF in the suspension was achieved by adjusting the pH, wherein the resultant long-range interaction of ChNF and CNF depended on the initial charge of the ChNF and CNF.^463^ Two distinct regimes that dominated the system were identified by isothermal titration calorimetry, including hydrophobic associations at increased pH values and ionic attraction at intermediate pH (Figure 26c). The assembly of neutralized ChNF that originated from hydrophobic associations was found to contribute strongly to an increased elastic modulus of the system, while ionic complex formation enhanced the stability of the ChNF/CNF assembly under broader pH conditions. These results demonstrate the possibility to construct pH-dependent nanochitin assemblies for versatile applications.

### 4.4 Process-Induced Nanochitin Assembly

In previous subsections, the assembly of nanochitin under multilevel assembly forces is described, highlighting the properties of nanochitin and prospects to control the microstructure and interfibrillar bonding upon assembly.^464^ However, it is still essential to consider low-cost and energy-efficient routes to process nanochitin, in both wet and dry forms. This is because at high concentrations the highly viscous nanochitin dispersion limits the homogeneity and irreversible aggregation occurs upon water evaporation. Thus, a thorough understanding of the mechanisms involved in process-induced nanochitin assembly is needed, for instance, to control drying and to simultaneously produce homogeneous redispersion, relevant to transport, storage, and redispersion of chitin nanomaterials.

**Figure 27:**
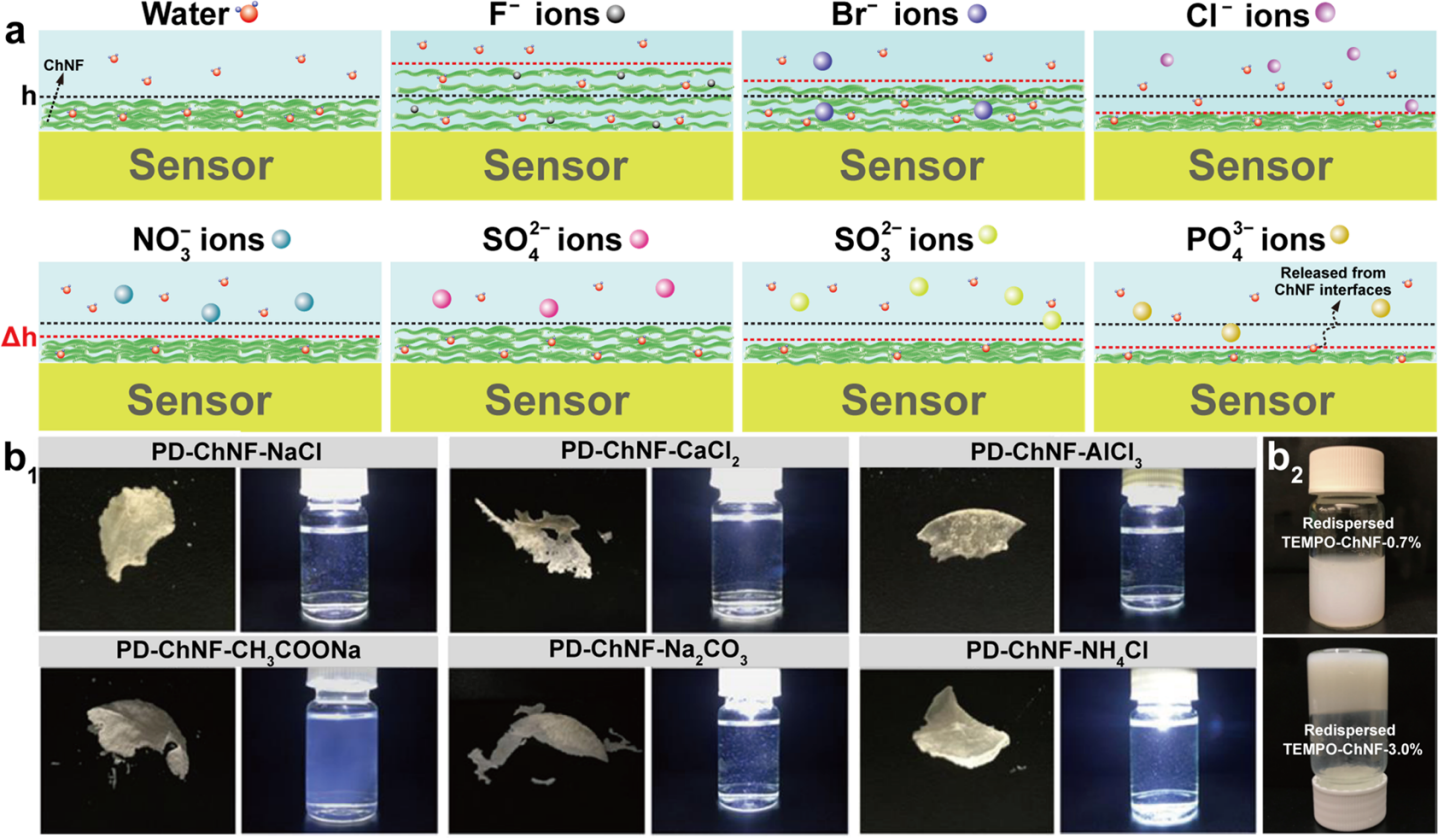
(a) Effect of the coupling of monovalent Na and multivalent anions on swelling/deswelling properties of ChNF films supported on a quartz crystal microbalance sensor. The black (*h*) and red (Δ*h*) dashed lines are drawn to show the thicknesses of ChNF film before and after exposure to electrolytes, respectively. Adapted from ref (466). Copyright 2021 American Chemical Society. (b1) Photographs of partially deacetylated ChNF (PD-ChNF) in the dry state (left panel) and redispersed in water (right panel) after the addition of different salts into the initial aqueous dispersions. (b2) Photographs of redispersed TEMPO-oxidized ChNF (TEMPO-ChNF) suspension at 0.7 and 3.0% concentration, respectively. Adapted from ref (278). Copyright 2018 American Chemical Society.

The interactions of nanochitin with water, relevant to dispersion and drying, as well as the strategies to manipulate such interactions, are fundamental to achieving control over processing. The strong intermolecular and interfibrillar H-bonding associated with nanochitin limit the penetration of water molecules in the internal of elementary chitin nanofibrils. Hence, nanochitin–water interactions depend heavily on the surface chemistry and are modulated by the effect of counterions in the system.^465^ The dynamics and strength of interactions between ChNF and water molecules have been investigated with quartz crystal microgravimetry using monovalent or multivalent counterions, including F^–^, Br^–^, Cl^–^, NO_3_^–^, SO_3_^2–^, and PO_4_^3–^ and in the presence of sodium.^466^ ChNF films equilibrated in water swelled with the addition of F^–^ ions. The same occurred for Br^–^ at low ion concentration, while deswelling occurred at increased ion concentration. Similarly, the thickness of ChNF films monotonically decreased when exposed to Cl^–^ ions (Figure 27a). Based on the Hofmeister series,^467,468^ and compared with Br^–^ and Cl^–^, the smaller F^–^ ions formed clusters with water molecules, were more electronegative, and displayed a stronger affinity with amino groups. Hence, interfacial water molecules were freed in the presence of F^–^ ions and produced an unequal anion distribution across the ChNF film, promoting swelling. The affinity with ChNF decreased with Br^–^ and Cl^–^, in the same order, so that they were less efficient to displace interfacial water molecules, leading to water expulsion and deswelling.^469^ These results indicate the possibility for controlling the swelling/deswelling behavior of ChNF films depending on the ion type and concentration. ChNF films deswelled in the presence of multivalent anions (NO_3_^–^, SO_3_^2–^, and PO_4_^3^), and a higher valence induced a larger effect (Figure 27a), which was a consequence of the relatively higher ionic strength upon dissociation in water.^470^ Given that the Debye length of charged particles is influenced by the ionic strength of the medium,^471^ a greater reduction of the EDL of ChNF was achieved in the presence of PO_4_^3–^, followed by SO_3_^2–^ and NO_3_^–^. The EDL compression in ChNF translates into conformational changes and a lower repulsion barrier. Accordingly, interfibrillar interactions supersede those with the surrounding water molecules, facilitating water exclusion from the film.

The control of nanochitin assembly and the effects of processing depend on nanochitin–water interactions and the counterion strength and type. Inorganic salts alter the stability of macromolecules dissolved in water,^472^ and it is established that electrolytes alter the lyotropism of biobased nanoparticles in aqueous suspension and subsequent structuring in the dried forms.^464,473^ Thus, exploiting this concept, the addition of given electrolytes to a ChNF aqueous suspension enables fractionation, drying, and eventual redispersion of the nanofibers without altering their properties.^278^ Partially deacetylated ChNF (PD-ChNF) and TEMPO-oxidized ChNF (TEMPO-ChNF) were used to evaluate the effect of counterions in adjusting the processability of ChNF. While the visual appearance of dried ChNF remained unchanged (Figure 27b1), nanofibers treated with sodium carbonate (Na_2_CO_3_) were readily and homogeneously redispersed in water under mild shearing. This observation can be rationalized in terms of the dispersion stability from specific counterion adsorption, nanoparticle association, and electrostatic-charge development. These effects play combined roles in ChNF assembly, interfibrillar H-bonding, and nonspecific interactions generated upon drying, endowing redispersibility.^474^ For TEMPO-ChNF, while no significant effect of salt type was noted, given weak interactions between anionic nanofibers and monovalent counterions (Na^+^ and NH_4_^+^), the properties of aqueous dispersions reconstituted from dried TEMPO-ChNF were tunable. A TEMPO-ChNF suspension at 0.7% concentration displayed low viscosity, while a highly viscous, gel-like system occurred at 3.0% (Figure 27b2). In sum, processing-induced assembly and interaction under the influence of salts allow for nanochitin to be dried and redispersed, which is of practical importance and necessary for upscaling.

## 5 Applications of Nanochitin in Multidimensional Materials

**Figure 28:**
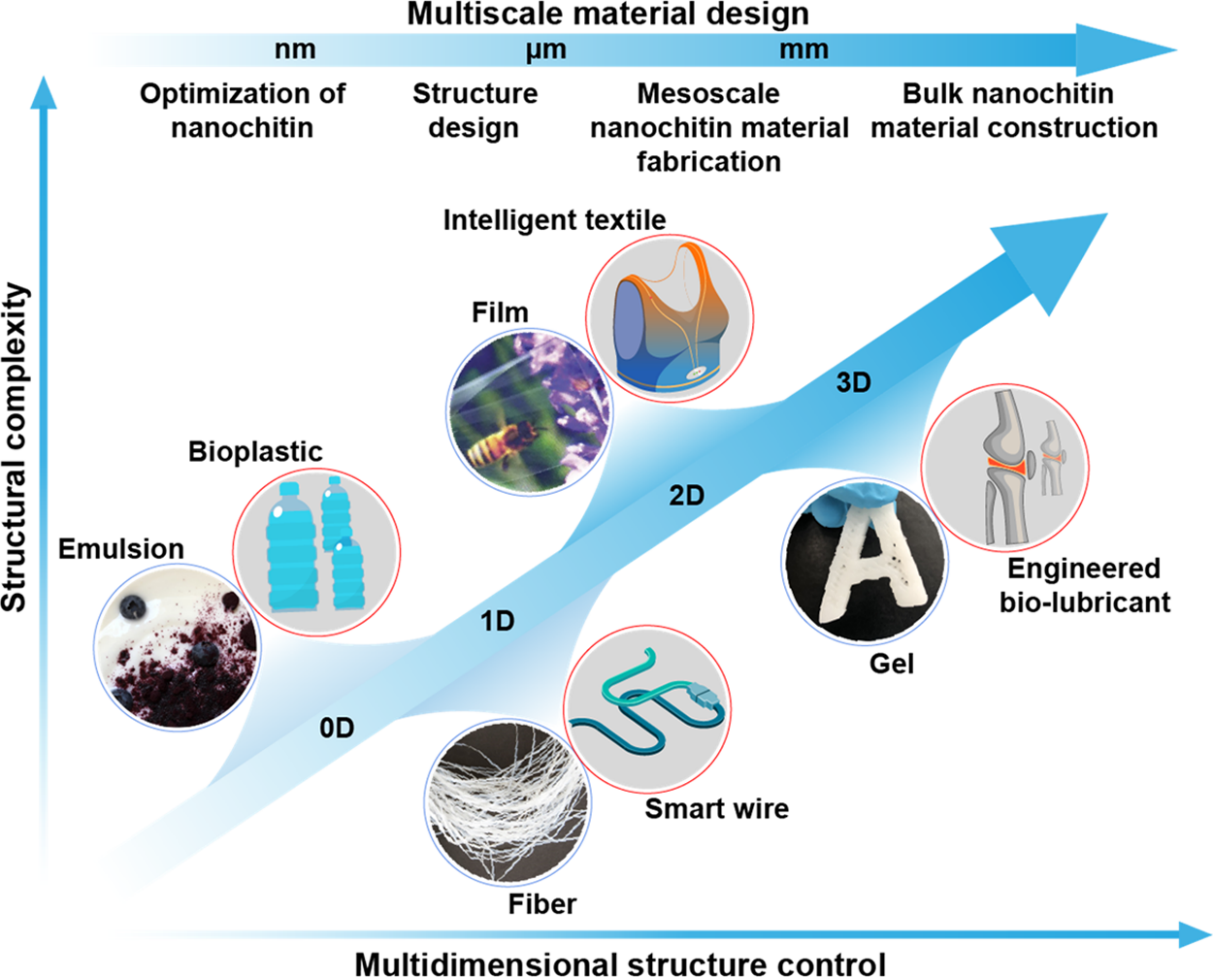
Roadmap toward multidimensional materials built from nanochitin with multiscale design principles, including current applications (blue circles) related to 0D emulsions, 1D fibers, 2D films, and 3D gels, and future perspectives (red circle) in 0D bioplastics, 1D smart wires, 2D intelligent textiles, and 3D engineered biolubricant. Here, “0D” refer to objects that make use of nanochitin as a primary building block. Adapted with permission from ref (478). Copyright 2017 John Wiley and Sons. Adapted from ref (479). Copyright 2018 American Chemical Society.

Nanomaterials have attracted attention as new class of materials, demonstrating great potential in diverse fields.^475^ However, biobased nanomaterials with properties and functions that compare with those produced from synthetic polymers have been challenging. Benefiting from its chemical and morphological attributes and excellent adaptability,^9,14^ nanochitin is a promising starting material for the fabrication of advanced nanomaterials.^476^ Hierarchical assemblies, from the primary nanofibrils to the bulk tissue found in chitin, inspire novel design strategies and rational fabrication approaches for creating nanochitin-based materials (Figure 28).^477^ Nanochitin shows a high level of spatial and structural sophistication, including helical and periodic arrangements over a wide range of length scales. Thus, an in-depth understanding of nanochitin assembly and interactions with an eye on the applications will significantly boost materials development. Nanochitin shows great promise in building materials at different dimensional aspects, including 1D fibers, 2D films, and 3D structured gels (Figure 28). In this section, the structure–process–property relationship of nanochitin-based materials will be examined from multidimensional aspects using various experimental techniques.

### 5.1 Application of Nanochitins as Building Blocks

The properties and functions of materials usually depend on the components and the processing techniques that are used in their fabrication. The building elements should be predictable and stable to enable reproducible and precise manufacture. These factors are far more important for nanochitin-based 0D applications due to the direct relationship between nanochitin and the end materials. Nanochitin shows great potential to fulfill these requirements in 0D applications, since chemical and morphological attributes encoded within chitin may be predicted according to the chitin source. In this subsection, the direct use of nanochitin as a building block for a variety of 0D applications will be discussed.

#### 5.1.1 Nanochitin as a Pickering Stabilizer

**Figure 29:**
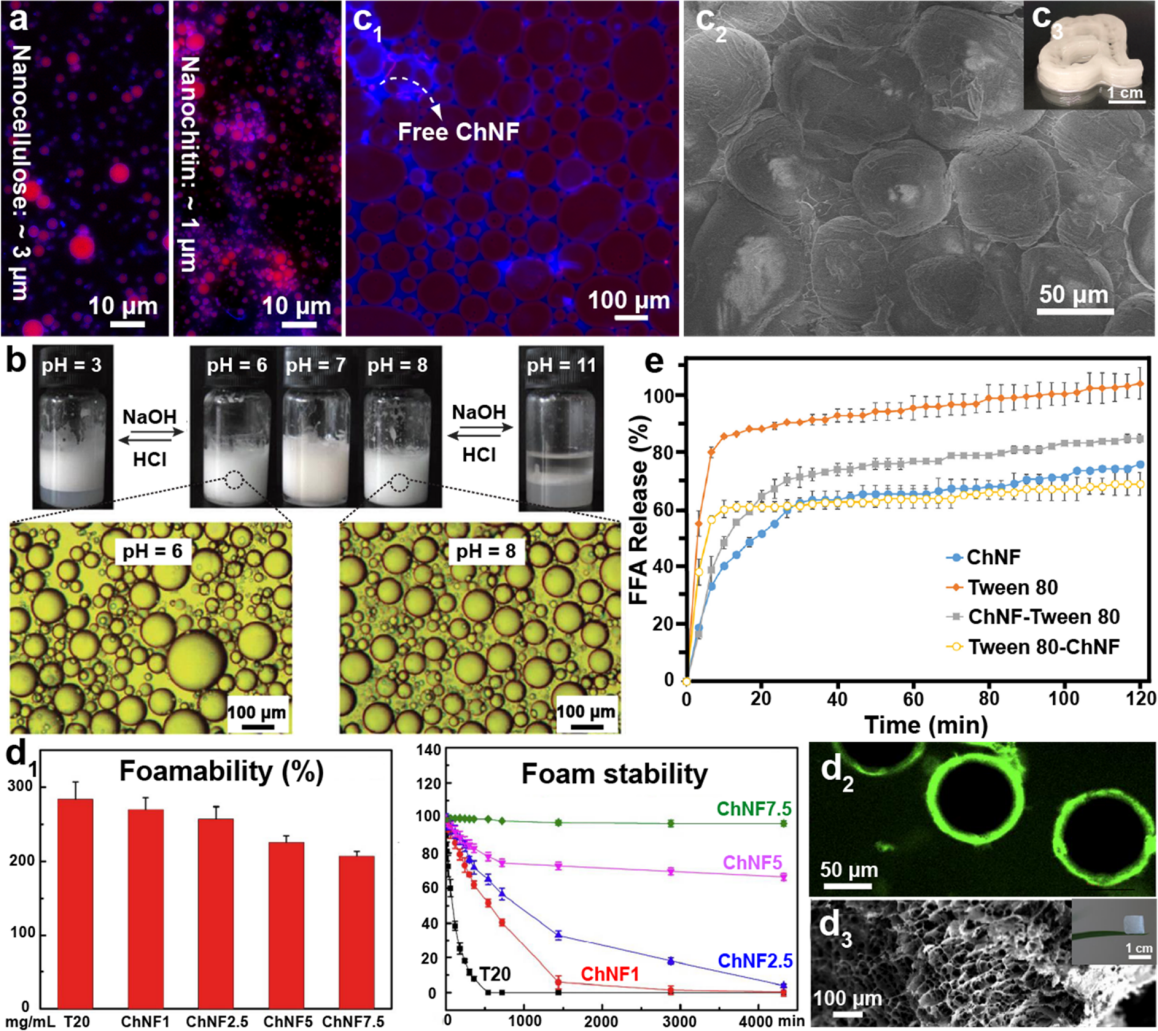
(a) Fluorescent microscopy images of Pickering emulsions stabilized by CNC and ChNF. Adapted with permission from ref (376). Copyright 2018 The Royal Society of Chemistry. Adapted from ref (273). Copyright 2019 American Chemical Society. (b) Visual appearance and optical microscopy images of pH-reversible, paraffin-in-water Pickering emulsions prepared at different pH using zwitterionic ChNF. Adapted with permission from ref (280). Copyright 2017 The Royal Society of Chemistry. (c1) Fluorescent microscopy images of HIPPE solely stabilized by ChNF. (c2) Cryo-SEM image of the HIPPE droplets (emulsions of 88% oil fraction) showing the distribution of ChNFs on the droplet surface and in the continuous phase. (c3) 3D-printed object produced from HIPPEs. In (a) and (c1), the nanopolysaccharides are stained blue and the oil phase is dyed red. Adapted from ref (410). Copyright 2020 American Chemical Society. (d1) Foamability (upper panel) and foam stability (bottom panel) of liquid foams stabilized by a mixture of a nonionic surfactant (Tween 20, T20) and ChNF at different concentrations (mg/mL). The T20 concentration was 0.5 wt % in all the samples. (d2) Confocal image of the liquid foam stabilized by FITC-labeled ChNF (7.5 mg/mL). (d3) SEM image of the cross section of ChNF-stabilized foam upon drying. The inset shows a photograph of a piece of solid foam on the leaf of a bracket plant. Adapted from ref (426). Copyright 2018 American Chemical Society. (e) Reduced free fatty acid (FFA) release of ChNF-stabilized Pickering emulsion under simulated small intestinal conditions. The emulsions named as “ChNF-Tween 80” or “Tween 80-ChNF” refer to the main droplet stabilizer, either ChNF or the nonionic surfactant (Tween 80), respectively. Adapted with permission from ref (498). Copyright 2020 Elsevier.

Pickering multiphase systems, emulsions and foams, have been used in formulating a range of materials, particularly in the fields of foodstuff, pharmaceuticals, and cosmetics.^428,480^ Nanochitin assembly at the oil/water interfaces brings unique attributes in the actual implementation of Pickering emulsions.^481^ One of the key emulsion properties toward applications is the droplet size.^482^ Compared to Pickering emulsions stabilized by other types of nanopolysaccharides, e.g., nanocelluloses, Pickering droplets are typically smaller when stabilized by PD-ChNF (Figure 29a) and show extended storage stability against coalescence.^273^ For example, hollow ChNF spheres can be synthesized using a ChNF-stabilized styrene-in-water Pickering emulsion as a sacrificial template, showing applications in encapsulation and delivery.^483^ While nanochitin usually stabilizes O/W Pickering emulsions, enhanced dispersibility of nanochitin in solvents via covalent grafting of hydrophobic alkane chains on the surface enables water-in-oil systems, extending the range of opportunities for Pickering stabilization.^335,359^ In this Review, O/W type nanochitin-based Pickering emulsions will be the main focus.

Apart from the simple use of nanochitin as a Pickering emulsion stabilizer, taking advantage of the naturally occurring cationic surface properties, nanochitin has been used to develop responsive emulsions. Following an in situ modification strategy, a sequential process of partial deacetylation (amine groups) and TEMPO-mediated oxidation (carboxyl groups) was applied to nanochitin, producing zwitterionic ChNF with switchable surface charges.^322^ Exploiting the changeable surface chemistry, a doubly pH-responsive paraffin-in-water Pickering emulsion, solely stabilized by zwitterionic ChNF, was achieved (Figure 29b).^280^ With changes in pH value, it is possible to achieve reversible emulsification and de-emulsification. Particularly, the droplet morphology can be altered upon a shift in pH (Figure 29b), making the zwitterionic ChNF attractive in the formulation of responsive emulsions.

Another application of nanochitin as a Pickering stabilizer is to generate tunable high-internal-phase Pickering emulsions (HIPPEs). Different from conventional emulsions, stabilization of HIPPEs often requires not only interfacial adsorption of nanoparticles but also the formation of a particulate network in the continuous phase.^484^ Such an effect fits nanochitin very well given its low critical gelation concentration.^335^ Exploiting Pickering stabilization of ChNC, an O/W HIPPE containing up to 96% internal phase (hexadecane) was successfully formed as a textured gel.^485^ In another study, ChNF was used to produce O/W HIPPEs with a volume fraction of the internal phase (food-grade sunflower oil) as high as 88% (Figure 29c1).^410^ The stabilization ability of ChNF originated from the restricted coarsening, droplet breakage, and coalescence upon emulsion formation, which were the result of both irreversible adsorption at the interface and structuring in highly interconnected fibrillar networks in the continuous phase (Figure 29c2). Furthermore, due to the elastic behavior and resilience to compositional changes, the HIPPEs were easily textured in, e.g., a direct writable ink suitable for molding and 3D printing (Figure 29c3).

In addition to Pickering emulsions that are solely stabilized by nanochitin, nanochitin-based composite stabilizers have been explored for the formation of multiphase materials.^422,486^ This is because the cationic nature of nanochitin facilitates modification using the more typical anionic nanoparticles derived from renewable resources.^487^ For instance, the stabilization ability of nanochitin was enhanced with the addition of CNF.^417,488^ Integration of ChNF with CNF facilitated the interfacial adsorption of CNF and the formation of a thick particulate layer at the droplet surface, restricting droplet coalescence during storage. In the meantime, the obtained CNF layer on the droplet surface provided a remarkable emulsion tolerance to environmental stresses, e.g., in the pH range between 3 and 11 and ionic strength between 100 and 500 mM, which significantly extends the application range of ChNF in the field of emulsions. Apart from rodlike nanoparticles, spherical zein colloid particles (ZCPs) have been used to modify the emulsifying capability of ChNF *via* H-bonding and hydrophobic interaction between the two components, ChNF and ZCPs.^489^ The addition of ZCPs to ChNF increased the probability of adsorption of ChNF at the oil/water interface, thereby providing a better steric hindrance between oil droplets. In another route, mixed Pickering emulsions have been considered by blending two types of oil droplets, with adsorbed ChNF and CNF, respectively.^490^ Under appropriate conditions, composite droplets were formed comprising a shell of ChNF around CNF-coated droplets, creating special emulsion textures. The latter system exhibited better coalescence stability compared with the single emulsion counterparts.

Apart from Pickering emulsions, Pickering foams have attracted great attention due to their ability to resist disproportionation, for days or weeks, compared to the lifetimes of typical systems (less than an hour for surfactant-stabilized bubbles).^424^ Indeed, ChNC-stabilized liquid Pickering foams, formed by ultrasonication^425^ or air injection,^427^ exhibited extended storage stability and limited degradation. However, although Pickering stabilization allows the conversion of liquid foams into solid counterparts while retaining the microstructure,^410^ this process is not possible in the case of ChNC-stabilized systems given the lack of resistance of the structure upon drying. The latter limit is related to weak ChNC interfibrillar adhesion. As a way to overcome this challenge, long, flexible ChNF has been used with the assistance of a nonionic surfactant, yielding long-lived liquid Pickering foams.^426^ With the increased ChNF loading, reduced foamability but improved foam stability was achieved, indicating the ability of ChNF to adjust the foam properties (Figure 29d1). The formation of gel networks by self-aggregated ChNFs at the air/water interfaces was also explored (Figure 29d2), significantly preventing coalescence and disproportionation. Accordingly, the formed interfacial network within foams facilitated the fabrication of solid porous matrices following the removal of water by air drying. On the other hand, porous solid ChNF-stabilized foams obtained from freeze-drying was ultralight and displayed a uniform microstructure (Figure 29d3). The method used for drying had a great impact on the mechanical properties of the porous nanochitin materials. For a similar density, foams obtained by air-drying displayed better mechanical performance compared to those obtained by freeze-drying. The results were explained by the more compact packing and restored H-bonding that were induced upon air drying.

Pickering emulsions are particularly suitable for developing functional delivery system designed to improve human health and well-being.^480,491^ Integration of unique chemical and morphological features of nanochitin enables the modification and development of functions. Consumers demand foodstuff with less or no synthetic preservatives, which positions natural agents as promising alternatives. Antimicrobial essential oils derived from plants offer a route to achieve all-natural systems.^492^ Nevertheless, direct incorporation of essential oils for use as preservatives is still rare due to low water solubility, high volatility, strong odor, *etc*.^493^ Due to the renewability and interfacial activity, as well as the antimicrobial effect, the nanochitin-stabilized Pickering multiphase system is a promising platform for efficiently delivering essential oils and, therefore, achieving antimicrobial functions. A ChNF-stabilized *Cinnamon cassia* oil-in-water Pickering emulsion showed a strong antimicrobial activity against *Escherichia coli*.^415^ This was attributed to the increased stability of the emulsion, which inhibited the volatility and oiling-off of essential oils. In the meantime, the release pattern of essential oils from the emulsions showed prolonged antibacterial activity and enhanced diffusion efficiency, which turned out to be a good encapsulation system for controlled release of the essential oils. This showed the promising advantages of nanochitin-stabilized Pickering emulsions as delivery systems. Moreover, Pickering emulsions can regulate the digestion of lipids.^494^ This is important in addressing obesity,^495^ for instance, by food formulations that induce satiety as dietary therapy. Nondigestible nanochitin is particularly suitable for this purpose because it produces highly stable lipid droplets with full interfacial coverage.^496^ The cationic characteristics of nanochitin make it suitable as an efficient agent to interact with various anionic components within the human gastrointestinal tract (GIT).^497^ Consequently, nanochitin forms physical barriers that prevent digestive enzymes from reaching lipids and reduce the activity of digestive enzymes, bile acids, or other gastrointestinal components. An in vitro human GIT model was used to evaluate the ability of ChNF-stabilized Pickering emulsions to regulate the lipid digestion, and a 30% reduction in lipid digestion rate was shown (Figure 29e).^498^ Besides the above-mentioned reasons, inhibition of lipid digestion using nanochitin systems may be also caused by the reduced lipid accessible area to lipases in nanochitin-coated droplets that aggregate in the GIT. This opens the possibility to develop high-satiety diets using nanochitin as a Pickering stabilizer. On the other hand, the bioaccessibility and bioavailability of the encapsulated oil-soluble nutrients may be affected given the limited release of the lipid phase upon digestion, which would be undesirable from a nutritional perspective.^499^ Overall, the potential impact of nanochitin-based Pickering emulsions should be considered in the formulation of functional foods.

#### 5.1.2 Nanochitin as a Functional Additive

**Figure 30:**
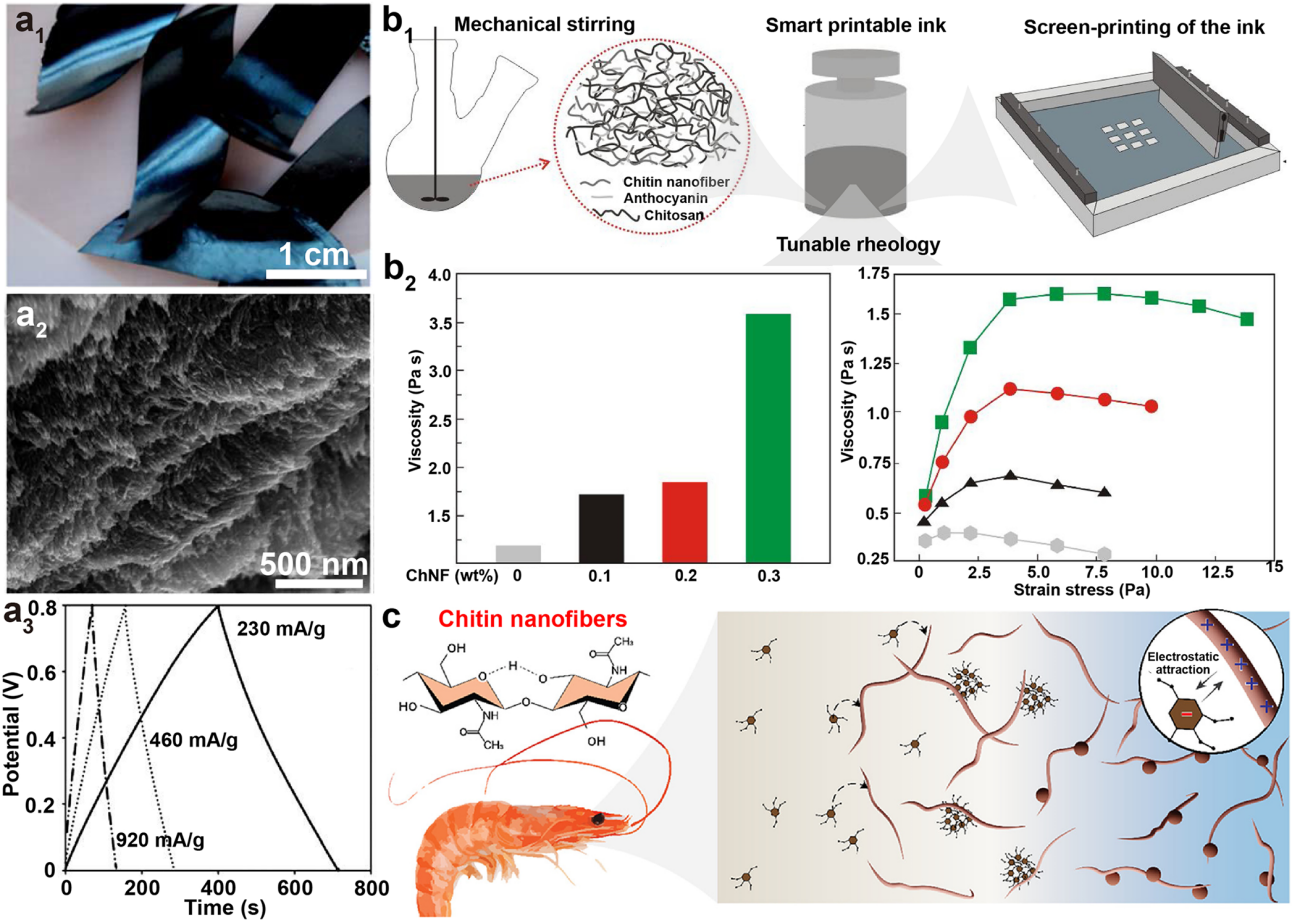
(a1) Photograph of mesoporous nitrogen-doped carbon films produced from nematic LCPs coassembled from ChNC and silica. (a2) SEM image of the cross section of the carbon film in (a1), showing a layered structure with embedding carbon nanorods in each layer. (a3) Galvanostatic charge/discharge curves of a typical carbon film in 1 M H_2_SO_4_ recorded at the different current densities. Adapted with permission from ref (458). Copyright 2014 The Royal Society of Chemistry. (b1) Schematic illustration of the preparation and application of the smart printable inks wherein ChNF was loaded in the aqueous phase as an additive to adjust the rheological behavior of the inks. (b2) (left panel) Viscosity at 0.1 s^–1^ and (right panel) yield stress of the inks at different ChNF loading levels (wt %). Adapted with permission from ref (516). Copyright 2020 Elsevier. (c) Schematic illustration of the solvent exchange process that leads to the nucleation and growth of lignin nanoparticles in the presence of shrimp-derived ChNF, used as templating support. Adapted from ref (520). Copyright 2021 American Chemical Society.

Nanochitin can be used as a functional additive or nanofiller,^338,500−504^ by taking advantage of the inherent chemical, morphological, and biological features of nanochitin, *e.g.*, surface charge and chemistry, anisotropy, flexibility, fibril dimension, aspect ratio, and nitrogen content.^505−508^ Hence, nanochitin enables novel material design,^384^ for instance, to reinforce different materials,^509,510^ and as a biomimicry template of periodic structures (ChNC LCP).^453,456,511^ This takes advantage of the combination of nematic structural properties of ChNC and a reinforcing phase to create advanced materials, showing potential uses in sensing, electrochemical, and optical applications, among others.^331,434,435,442,512^ Templating ChNC LCP was used to synthesize silica-based mesoporous nitrogen-doped carbon films in a layered nematic organization (Figure 30a).^458^ By transferring the long-range ordered organization of helicoidal ChNCs to the solid phase, a carbon film was produced and used as electrode in a supercapacitor that showed an improved electrochemical performance (Figure 30a).

The geometrical features of nanochitin enable interesting flow behavior in suspension,^273^ similar to that of nanocelluloses,^513^ which depends on the solid content as well as the detailed morphology and interactions between the particles.^514^ Particularly, nanochitin suspensions become viscoelastic at relatively low solid content,^273^ showing great potential in rheology modification.^462^ Partially deacetylated nanochitin has been successfully used to adjust the rheological behavior of alginate-based solutions.^515^ The electrostatic interaction between oppositely charged components enabled the reassembly of fibril-like chitin nanoparticles surrounding alginate molecular chains. Such systems behaved as non-Newtonian fluids, in which an increased solids content resulted in an increased yield stress, higher Newtonian viscosity, and longer relaxation time. The main contribution of rheological modification is the possibility to enable control on the flow behavior of the materials,^479^ which can result in suitable properties, *e.g*., for printing-based manufacturing. In this respect, ChNF has been used to endow excellent printability to anthocyanin-containing smart inks, e.g., by adjusting the rheological properties, including yield stress and shearing viscosity (Figure 30b).^516^ Immobilization of anthocyanin and enhancement of the rheological behavior of the composite ink were achieved by the formation of H-bonding between different components.

The cationic surface of nanochitin offers unique opportunities in applications where electrostatic attraction between oppositely charged components is required.^196,517,518^ For instance, incorporation of ChNF with carboxymethyl cellulose enhanced the mechanical and antimicrobial performance of films given electrostatic coupling effects.^519^ The positively charged surface of ChNF and the inertness of the formed networks allow templates suitable for heterogeneous reaction and in situ formation of nanoarchitecture in aqueous media. For example, ChNF-templated production of lignin nanoparticles (LNPs) was achieved following the solvent shift approach (Figure 30c).^520^ The surface chemistry of the templating ChNF dictated the dynamics of nucleation and growth of LNPs. A strong interfacial electrostatic interaction between the oppositely charged ChNF and lignin molecules facilitated instantaneous and extensive lignin adsorption, followed by nucleation and growth into nanoparticles. As expected, electrostatic attraction enabled a strong coupling of the formed nanoparticles to the ChNF support while retaining their homogeneous distribution in the suspension.

### 5.2 Fibers and Filaments

1D materials, including fibers and filaments, are often produced from synthetic materials, usually nonrenewable and nondegradable.^475^ Benefiting from manufacturing methods that use biobased nanoparticles, following spinning techniques,^521^ it is possible to develop 1D fibers. This has been demonstrated for nanocelluloses,^522,523^ yielding systems with performances that compare to those of synthetic ones.^524^ Inspired by these advances, nanochitin has been considered for constructing 1D fibers. The cationic nature of nanochitin brings unique functional attributes to biobased 1D fibers. Moreover, spinning enables regulation of the structure and orientation of the starting material,^22^ offering the possibility of creating nanochitin-based fibers with special performances and novel functionalities. This subsection discusses the performance of nanochitin in 1D fibers, which are obtained by different spinning methods.

#### 5.2.1 Wet-Spinning

**Figure 31:**
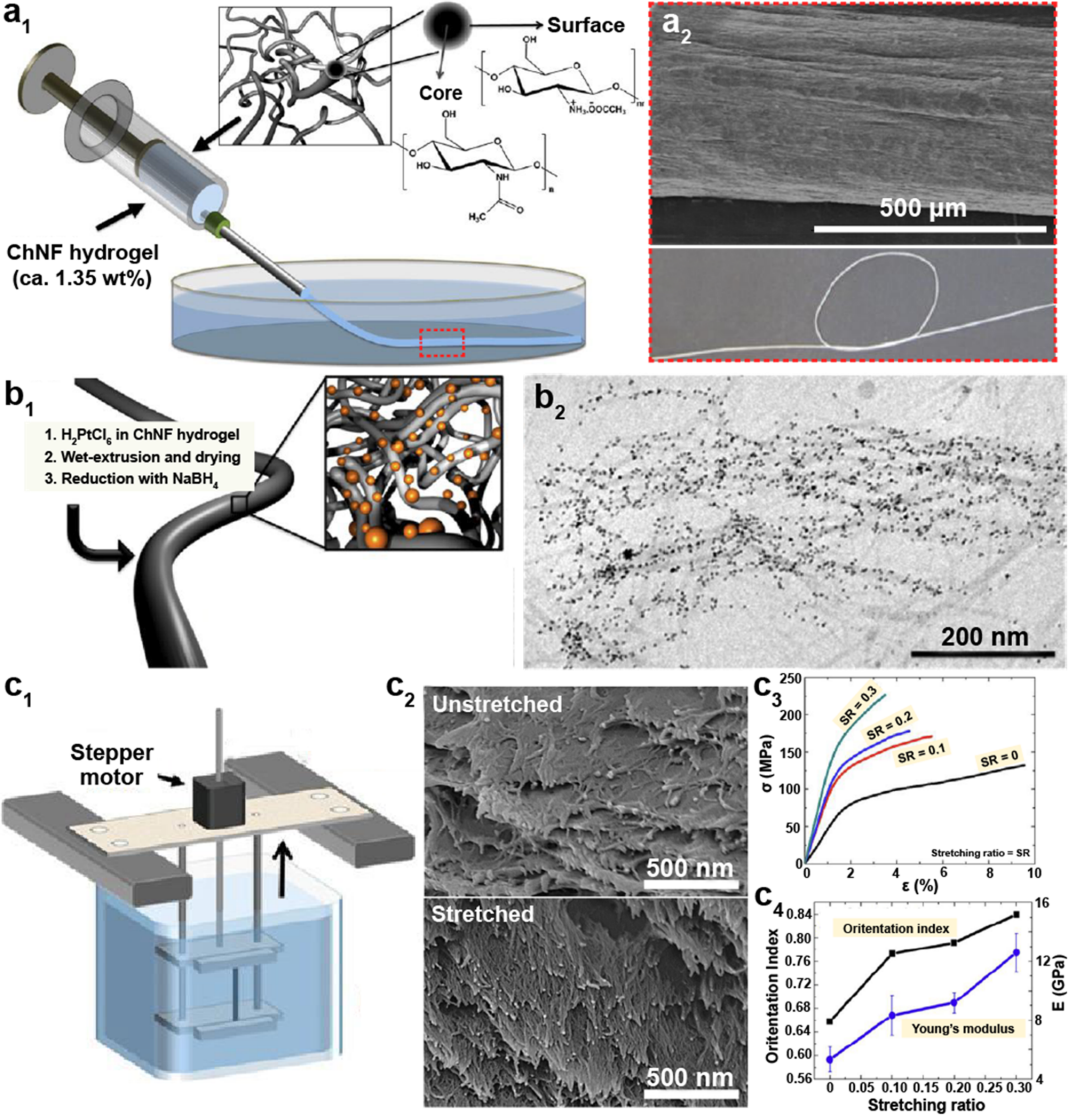
(a1) Schematic illustration of the preparation of chitin-based macrofibers or filaments by extrusion of ChNF hydrogels into a coagulation bath and drying. (a2) SEM and digital images of the as-prepared macrofiber. (b1) Schematic illustration of structure of the hybrid macrofibers that were extruded from a mixture of ChNF and metal ions. (b2) Cryo-TEM image of metal nanoparticles bound to ChNF aggregates in the gel state. Adapted from ref (527). Copyright 2012 American Chemical Society. (c1) Schematic drawing for wet-stretching device into which a macrofiber (as shown in a1) is clamped in water and stretched under controlled strain rates. (c2) SEM images of a cross section of unstretched and stretched ChNF-base macrofibers. The stretching ratio was set to 0.3. Influence of stretching ratio on (c3) the tensile mechanical properties and (c4) orientation index and Young’s modulus (*E*) of ChNF-based filaments. Adapted from ref (532). Copyright 2014 American Chemical Society.

Wet-spinning is a strategy to assemble nanostructures into 1D filaments by fast and efficient dehydration of aqueous dispersions or hydrogels,^523^ suitable for operation in large scales.^525^ In this process, a spinning dope is extruded through a spinneret into a coagulation bath, in which the dope solidifies into a filament through rapid solvent exchange and solidification. Biobased nanofibrillar systems have been used in wet spinning, for instance, with CNF;^526^ extending such a technique to hydrogels containing surface-deacetylated ChNF enables production of micrometer-wide filaments (Figure 31a1) that exhibit flexibility (Figure 31a2). For this purpose, the antisolvent used in the coagulation bath can be ethanol or THF.^389,527^ Antisolvents such as acetone, sodium hydroxide, or ammonium solutions also enable ChNF filaments with diameters in the micrometer range,^528,529^ depending on the spinning parameters. Heterocoagulation of oppositely charged components has been used to generate nanochitin-based filaments by wet-spinning.^530^ In this strategy, a suspension mixture of carbon nanotubes and TEMPO-oxidized CNF (TEMPO-CNF) was extruded into a ChNF suspension to form filaments where their formation was triggered by electrostatic attraction.

One interesting finding from wet-spun nanochitin-based filaments is that once dried they become difficult to redisperse in water. This is attributed to the strengthened interfibrillar H-bonding between ChNFs upon spinning and drying.^161^ The high water resistance of nanochitin-based filaments, together with their internal porous structure, enable a high water sorption capacity.^528^ Such systems can be also considered as a platform for cell cultue, showing excellent proliferation and viability for given cell lines. Apart from the physical contribution of nanochitin, its surface chemistry also allows additional functionalities, e.g., upon hybridization with catalytically active noble metal nanoparticles (Figure 31b).^527^ The latter has been demonstrated for cyclic, fast catalytic reduction of model compounds with no loss of activity.

One bottleneck for practical applications of nanochitin-derived 1D filaments is the limited mechanical strength. Indeed, although macrofibers fabricated from ChNF display good mechanical properties with large plastic strain,^527^ they are still weak compared with wet-spun filaments produced from CNF.^531^ This motivates the design of ingenious spinning processes to tailor nanofiber orientation and mechanical performance. Wet-spinning allows control of the structure and orientation of nanofibers,^22^ providing a pathway to improve the mechanical properties. By controlling the flow regime and dehydration of the nanofibers before, during, and after spinning, they can be spun into highly oriented filaments. The orientation and alignment of nanofibers are influenced by many factors, and a strong interfibrillar cohesion is desirable. Due to the strong interparticle repulsion, this effect is challenged in the case of highly charged ChNF. To overcome this issue, postdrawing of wet-spun nanochitin filaments has been proposed to further improve the mechanical strength (Figure 31c1).^532^ A strain-rate controlled wet-stretching of rehydrated macroscale filaments composed of ChNF induced a high degree of nanofiber orientation and alignment (Figure 31c2), favored by strongly disengaged networks that were swollen in water. As a consequence, the mechanical performance of the obtained filaments was improved upon stretching (Figure 31c3) and the macroscale mechanical stiffness increased with the orientation index, reaching values as high as 12 GPa (Figure 31c4).

#### 5.2.2 Dry-Spinning

**Figure 32:**
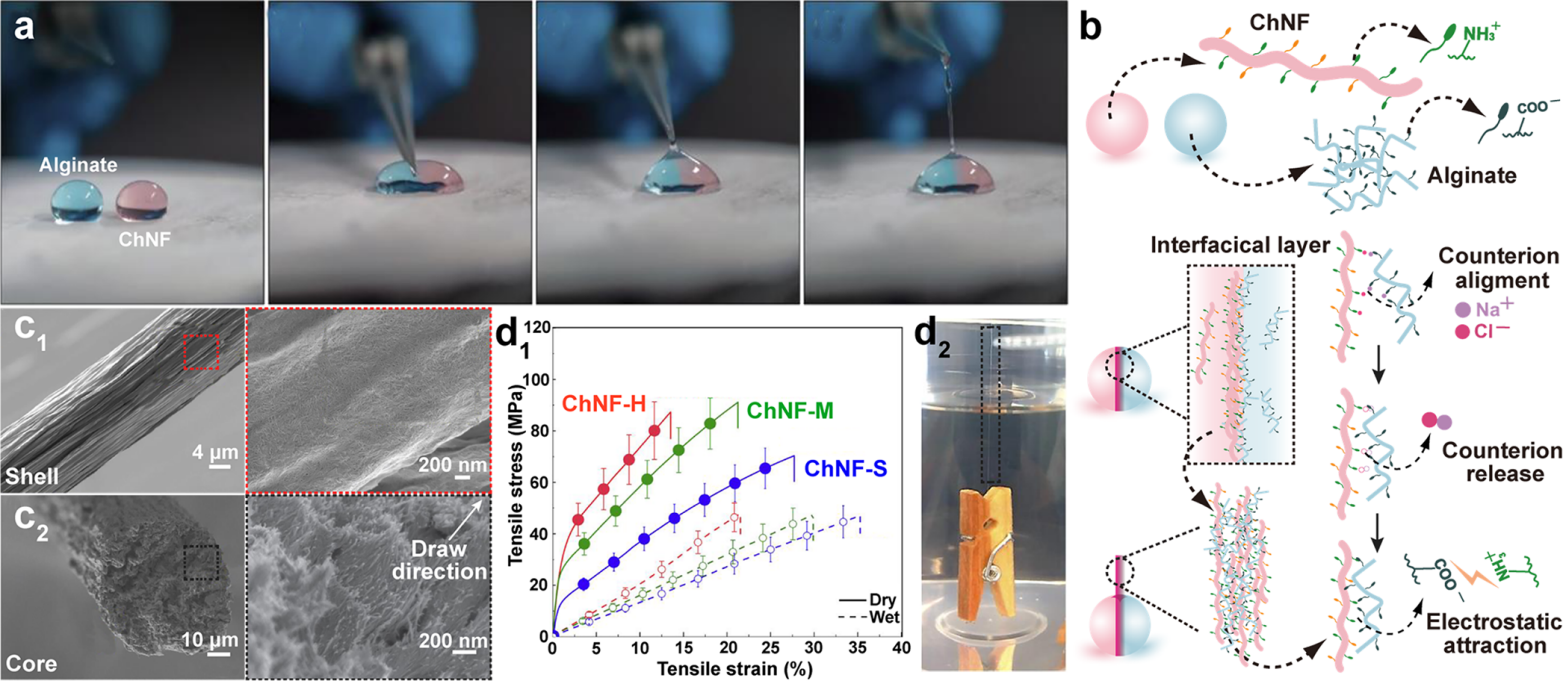
(a) Dry-spinning produce filaments by direct drawing of a viscous interface generated by contact of a ChNF suspension (red) and an alginate solution (blue). (b) Schematic illustration of the mechanism responsible for interactions upon interfacial complexation of ChNF and alginate. SEM images of (c1) surface shell and (c2) cross section core of dry composite filament. The dashed squares correspond to a higher magnification, and the arrow in the magnified image in (c2) shows the drawing direction. (d1) Tensile tests of filaments produced from ChNF with varying aspect ratios, in dry and wet conditions. ChNF-S, -M, and -H stand for ChNF with small, medium, and high aspect ratio, respectively. (d2) Visual appearance of a single filament (red dashed square) immersed in water and under load, demonstrating wet stability. Adapted from ref (537). Copyright 2020 American Chemical Society.

Dry-spinning is a process where solidification of generated 1D fibers occurs in air, through evaporation of the solvent or after a cooling process.^521^ This is similar to the natural process where aqueous solution is directly extruded into air at ambient temperature and low hydrostatic pressure. In one process, dry-spinning is used to produce filaments by ionic interactions between polyelectrolytes bearing opposite charges.^533^ This approach exploits the spontaneous self-assembly of the polymers into filaments by simply drawing the complexes that form at the fluid/fluid interface.^534^ This process has been employed to fabricate filaments consisted of biobased nanoparticles, e.g., nanocelluloses,^535,536^ referred to as “interfacial nanoparticle complexation, INPC”. Transferring this technique to nanochitin, filaments have been continuously drawn following INPC at the interface between two contacting suspensions containing ChNF and alginate (Figure 32a).^537^ The main complexation mechanism involves electrostatic interactions and entropic effects that originate from the release of counterions and water expulsion upon contact between ChNF and alginate, as illustrated in Figure 32b. The resulting composite microfibers or filaments exhibited a core–shell structure, wherein ChNFs were arranged in a hierarchical assembly comprising axially aligned nanofibers (Figure 32c1). A more randomly oriented shell was formed by a sleeve of alginate chains (Figure 32c2). Taking advantage of the unique morphological characteristics and the possibility to control the aspect ratio of ChNF, it was possible to tailor the mechanical performance of the as-spun microfibers. Thus, filaments prepared from ChNF with a large aspect ratio displayed the highest Young’s modulus, nearly doubling that found in filaments produced from the shorter fibers (Figure 32d1). Mechanical tests conducted underwater demonstrated the wet stability of the filaments (Figure 32d2), with <50% strength loss and up to 35% strain gain. Apart from the nanoparticle/polymer pair, the nanoparticle/nanoparticle system, for instance, ChNC and TEMPO-CNF, has been used in INPC, demonstrating the efficiency and universality of this approach.^538^ More importantly, functional components, e.g., carbon nanotubes, could be easily embedded into the microfibers by incorporating either ChNC or TEMPO-CNF, forming functional filaments. In sum, dry-spinning provides a new perspective toward the creation of high-performance nanochitin-derived 1D filaments.

#### 5.2.3 Electrospinning

Electrospinning of a viscoelastic fluid through electrostatic forces, following with fiber solidification with an evaporating solvent,^539^ has been used as a simple method to produce filaments and respective webs.^540^ The process leads to ultrafine fibers with diameters down to submicrometer or nanometer scales,^541^ and with the possibility of controlling fiber properties by adjusting the spinning parameters (e.g., solution and electrospinning setup).^542^ Electrospinning has been used to fabricate fibers from biobased nanoparticulate components,^543−545^ including nanochitin, for applications in the biomedical field such as wound dressing,^546^ antimicrobials,^198,540^ and cell culturing platforms.^547^ ChNC exerts a strong affect on the spinnability of multicomponent spinning suspensions, leading to different filament characteristics.^540^ This is the case, for instance, for ChNC/poly(ethylene oxide) composite systems.^548^ Overall, expanding from wet- or dry-spinning, electrospinning is known for its capability to produce nanochitin-containing filaments and webs.

### 5.3 Films

Considering the morphology of nanochitin and its relatively large aspect ratio, one can expect that self-entangled or aggregated nanostructures can be formed by simply concentrating or drying the precursor suspensions.^196,474^ Thus, nanochitin is suitable as a building block for creating films.^18,549,550^ As such, structural nanochitin composites have been reported with biobased or synthetic polymers.^508^ Nanochitin films can be manufactured from suspension via casting,^358^ filtration,^551^ dialysis,^245^ and spray-coating.^552^ In these processes dewatering, water evaporation, annealing, and hot pressing are necessary to consolidate the films. Generation of structural coatings on given supports offers another route to create nanochitin-based 2D materials.^553^ This subsection introduces some aspects relevant to the formation of nanochitin-based films and coatings.

#### 5.3.1 Films Obtained from Nanochitin Suspensions

**Figure 33:**
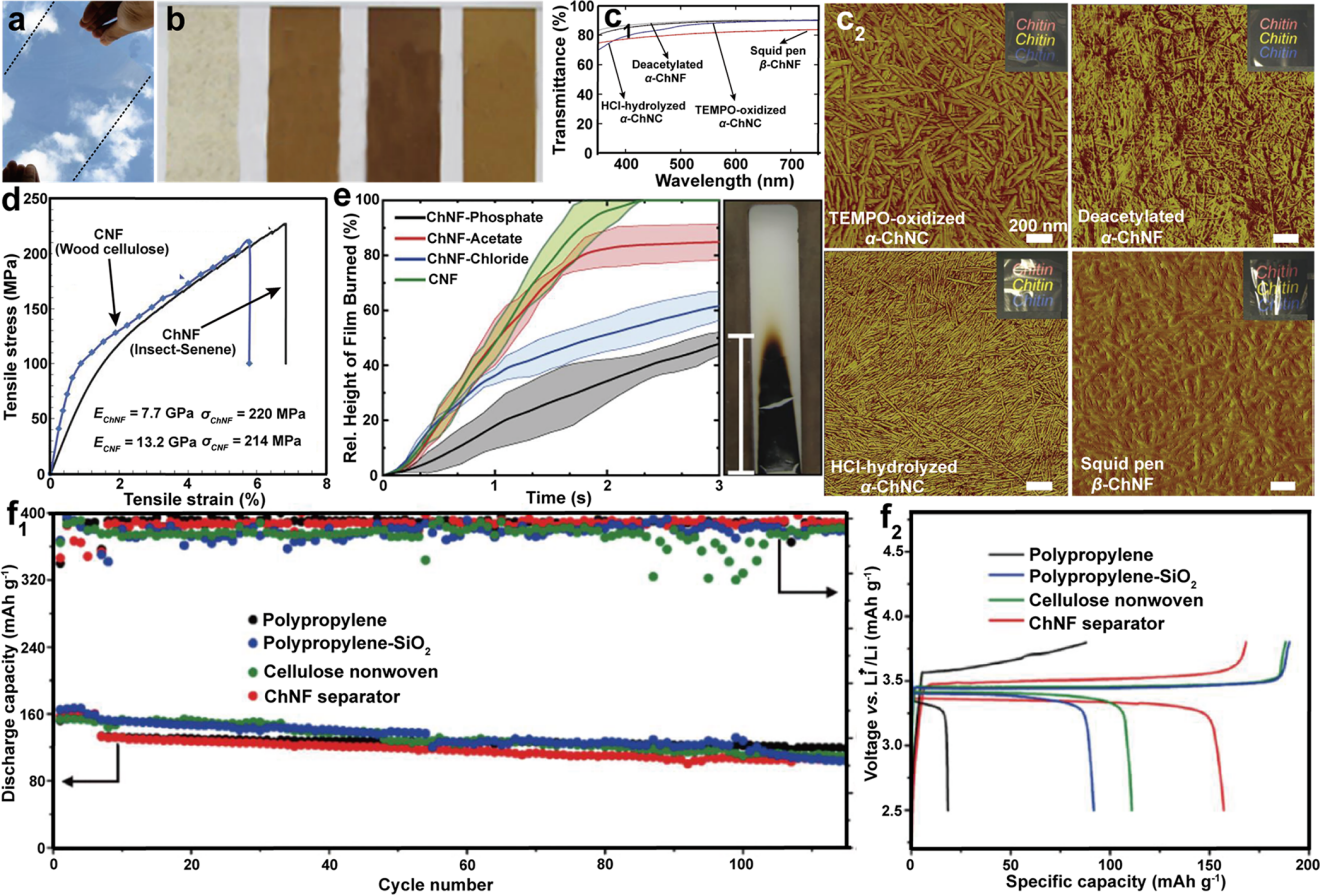
(a) Visual appearance of an optically transparent and flexible ChNF film produced from α-ChNF. Adapted with permission from ref (245). Copyright 2013 Elsevier. (b) Optical appearance of 80 g/m^2^ nanopapers with a thickness between 60 and 80 μm produced from *Cancer pagurus* and *Agaricus bisporus* stalk, cap, and whole mushroom (from left to right), respectively. Adapted from ref (555). Copyright 2019 American Chemical Society. (c1) UV–vis transmittance and (c2) AFM images of self-standing films (25 μm thickness) of TEMPO-oxidized ChNC, deacetylated ChNF, HCl-hydrolyzed ChNC, and squid pen ChNF. Adapted with permission from ref (557). Copyright 2012 Elsevier. (d) Comparison of tensile strength and Young’s modulus of insect-derived ChNF and wood-derived CNF, indicating comparable mechanical properties of ChNF and CNF. Adapted from ref (257). Copyright 2020 American Chemical Society. (e) Time-dependent evaluation of vertical flame tests for different ChNF samples (CNF sample used as a reference). Note: the shaded areas correspond to the confidence intervals from triplicated tests. The image shows the burning height of ChNF-phosphate nanopaper after exposure to a flame for 3 s. Adapted from ref (465). Copyright 2019 American Chemical Society. (f1) Cycling performance of cells with using polypropylene, polypropylene-SiO_2_, cellulose nonwoven, and ChNF separators operated at 17 mA g^–1^ for the first five cycles (used for activation) and 85 mA g^–1^ for the following cycles. (f2) Charge/discharge voltage profiles of the LiFePO_4_/Li half-cells using different separators under 58.5 mA g^–1^ rate at 120 °C. Adapted from ref (574). Copyright 2017 American Chemical Society.

Nanochitin can be directly applied as a versatile building block to produce films. The latter can be optically transparent following dense packing and reduced light scattering/adsorption (Figure 33a).^253,554^ Films produced from mushroom-derived ChNF (containing glucans) were translucent and exhibited a strong brown color originated from melanin contained in the spores and gills of the mushrooms (Figure 33b).^169,555^ Moreover, porous and aggregated structures produced a mismatch in the RI between air and nanochitin, producing strong light scattering and leading to opaque films.^556^ To obtain highly transparent films, nanochitin of suitable structural dimensions and with proper annealing processes should be considered. For instance, different types of finely isolated nanochitin, including TEMPO-oxidized α-ChNC, deacetylated α-ChNF, HCl-hydrolyzed α-ChNC, and squid pen β-ChNF, produced films by suspension casting; all of the films showed high light transmittance (Figure 33c1).^557^ Highly transparent films were produced from deacetylated α-ChNF with a high aspect ratio, which showed very limited nanofiber aggregation (Figure 33c2).^558^

Besides visual appearance, mechanical strength is a key factor when considering a nanochitin precursor for film manufacture.^559^ A film produced from deacetylated ChNF reached a tensile strength of 140 MPa, Young’s modulus of 5 GPa, and elongation break at 10%.^557^ To improve the elongation, a plasticizer, glycerol, was added to the suspension prior to film formation, increasing the elongation to 16.3% while maintaining high light transmittance and low thermal expansion coefficient.^560^ The tensile strength of films from low-protein ChNF reached a higher value, 153 MPa with a similar modulus (7.3 GPa), likely due to the presence of protein, which engaged in strong interactions with the nanofibers to limit their interfibril slippage.^259^ Mechanical strengthening of ChNF films has been achieved following better individualization of ChNF.^561^ This can be carried out by using high-pressure homogenization in the presence of chitosan, which under acidic conditions acted as a sacrificial polymer to facilitate fibrillation and to prevent aggregation. Films produced from these nanofibers contained certain residual chitosan, showing improved tensile strength (187 MPa) and work of fracture (12.1 MJ/m^3^). These results relate to the nanoscale network structure and the better individualization of ChNF stabilized by the residual chitosan. It is shown that single α- and β-chitin nanofibrils display a tensile strength of 1.6 and 3 GPa, respectively,^42^ indicating that, in addition to the effect of network formation, the isolation of nanochitin from native chitin, e.g., by using strong processing methods, deteriorates the mechanical strength. ChNF films with high tensile strength (and with high optical transmittance > 90%) were achieved for α-chitin (220 MPa)^257^ and β-chitin (277 MPa)^258^ through minimizing chitin degradation during the process of disintegration, *i.e*., preserving the inherent strength of chitin. The colloidal stability of the nanofiber suspension also led to a dense packing with low number of defects; the films, in fact, were as strong as those from CNF (Figure 33d).^562^ The modulus of ChNF film was about half that of CNF-based films, which was caused by the inherent differences in the modulus of chitin (which is lower than that of cellulose).^563^ In addition, the acetyl substitution on the surface of nanochitin may also be a factor that reduces interfibril bonding.

Robust ChNF films with homogeneous mesoporous structures have been utilized in applications related to advanced materials.^564^ For example, highly flexible and optically transparent α-ChNF films had low gas permeabilities (O_2_: 0.006 barrer and CO_2_: 0.018 barrer),^253^ comparable to those of commercial poly(ethylene terephthalate) (PET) with permeabilities of O_2_ at 0.015–0.076 and CO_2_ at 0.08–0.15,^565^ demonstrating the promise of ChNF in the design of sustainable barrier packaging. Inspired by the underwater superoleophobicity of shrimp shells, a novel filtration membrane was fabricated from TEMPO-ChNF (obtained from shellfish waste), also with antifouling behavior.^566^ The resultant ChNF membranes exhibited excellent mechanical strength, recyclability, thermal stability, pH-resistance, superhydrophilicity, and underwater superoleophobicity. Thus, together with the tunable thickness and pore size, such membranes were effective platforms for separation of O/W emulsions, showing high separation efficiency (>95%) and water flux (>1500 L·m^–2^·h^–1^·bar^–1^). Deacetylated ChNF was used in filtration membranes with a chelation ability given the amino groups on the chitin surface, allowing effective extraction from O/W emulsions of noble metal ions, such as Au^3+^, Ag^+^, Pt^4+^, and Pd^2+^.^567^ After in situ reduction of metal ions adsorbed on the membranes, metal nanoparticle loaded ChNF membranes were obtained, with catalytic activity for applications as biosensing and as green catalyst.

One of the limitations for biopolymer films is their flammability, often requiring chemical modifications or additives to endow fire-retardancy. The intrinsic elemental composition of chitin, containing nitrogen, from abundant −NH_2_ and −CHNO- groups, provides an inherent flame-retardancy feature given that the formation of ammonia during combustion results in an increased thermo-oxidative stability.^568^ For instance, phosphorylated ChNF was used as a novel flame retardant and enabled self-extinguishing behavior of flammable papers, with no afterglow.^569^ Further exploiting the unique chemical characteristics of chitin, additive-free, flame-retardant, self-extinguishing, and strong nanopapers were developed from surface-deacetylated ChNF. Therein, the synergy between water evaporation and counterion exchange was quite relevant.^465^ The counterions were exchanged using the respective acid, providing electrostatic stabilization. The flammability of the ChNF nanopaper was critically reduced by exchanging the counterions with phosphate, which, comparing with those of the acetate or chloride types, significantly improved the flame-retardancy (Figure 33e). This was associated with the beneficial elemental combination of high nitrogen/phosphorus atoms in the final nanopapers, endowing halogen- and heavy metal-free flame-retardant materials.^570,571^ Furthermore, in situ hybridization of ChNFs with nanoclay platelets yielded composite nanopapers with exceptional shape-persistence and fire-barrier properties against direct exposure to a gas torch and increased the stiffness by 40% (Young’s modulus) compared to the pure ChNF nanopaper. These ChNF-based nanopapers represent a significant advance in the field.

There is great demand for battery separators (between cathodes and anodes) in energy devices.^572^ Key factors in such separators relate to the environmental friendliness and eco-efficiency as well as high mechanical strength, excellent thermal stability, and good electrolyte wettability. ChNF films show great promise to fulfill these demands.^573^ For instance, a new type of separator for Li/Na-ion batteries was proposed through the self-assembly of ChNFs in nanofibrous membranes, associated with the adjustment of the pores (by using a pore generation agent, sodium dihydrogen citrate).^574^ The pore size of the ChNF membranes was optimized with the pore agent, which prevented tight stacking of nanofibers during water evaporation and promoted multiple channels within the films. As a result, the electrochemical performance of the LiFePO_4_/Li half-cell with a ChNF membrane separator was comparable to that of other separators (including commercial polypropylene). For example, excellent specific discharge capacity and Coulombic efficiency of the cell were found during cycling tests when using ChNF separators (Figure 33f1). In addition, such a system showed a much better performance at 120 °C (Figure 33f2), indicating the potential of the ChNF separator for high temperature Li-ion batteries, given the excellent thermal stability, mechanical strength, and electrochemical stability at elevated temperatures. Following this path, chemically modified cyanoethyl-ChNF was used in high-performance separators,^341^ which not only exhibited great ionic conductivity but maintained excellent mechanical strength. The mechanism of high Li^+^ ion transport in the cyanoethyl-ChNF membrane was investigated through DFT calculation, which was found to be related to the weakening of Li^+^ ion binding over that of PF_6_^–^ ions, given the cyanoethyl modification.

#### 5.3.2 Films Obtained from Nanochitin Composites

**Figure 34:**
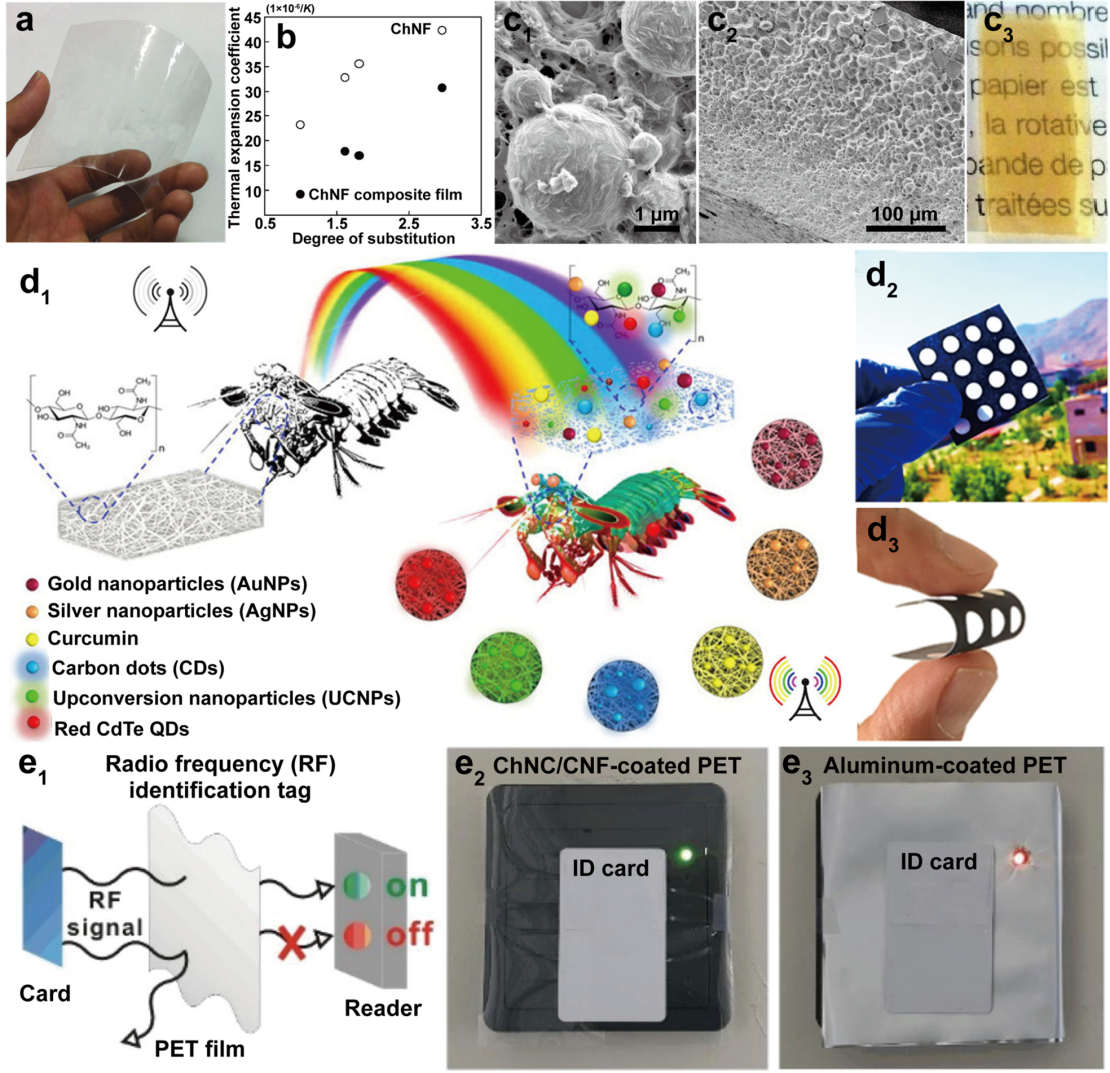
(a) Visual appearance and flexibility of transparent ChNF composite film containing a methacrylic resin. Adapted with permission from ref (578). Copyright 2011 The Royal Society of Chemistry. (b) Coefficient of thermal expansion of a series of acetylated ChNF and ChNF composite films. Adapted from ref (336). Copyright 2010 American Chemical Society. (c1) SEM micrograph of UV-cured acrylated epoxidized soybean oil droplet that is emulsified using ChNC in the presence of a photoinitiator. (c2) Cross-sectional SEM image of UV-cured ChNC composite polymer film fractured in liquid nitrogen, showing a well-preserved, spherelike beads. (c3) Visual appearance of UV-cured ChNC composite film. Adapted rom ref (592). Copyright 2021 American Chemical Society. (d1) Schematic illustration of the fabrication of different ChNF nanopaper-based composites via embedding/immobilizing various types of nanoparticles/components, which are suitable for a range of optical (bio)sensing applications. (d2) Visual appearance and (d3) flexibility of a ChNF nanopaper-based sensing platform, created with an office laser printer via direct-printing the hydrophobic ink onto a ChNF nanopaper, endowing hydrophobic and hydrophilic test zones. Adapted from ref (551). Copyright 2020 American Chemical Society. (e1) Schematic illustration showing a radiofrequency (RF) identification tag used for card reading through (e2) ChNC/CNF- and (e3) aluminum-coated PET film indicating the capability of ChNC-based nanocoating to transmit information. Adapted from ref (605). Copyright 2019 American Chemical Society.

Nanochitin films produced from neat suspensions can be tailored to achieve high transparency and mechanical strength. The design and development of composite nanochitin films consider the incorporation of secondary components to fabricate high-performance films,^575^ also adding new functions.^342,576,577^ For instance, optically transparent ChNF films that include (meth)acrylic resins, using either pristine or acetylated ChNF, were developed (Figure 34a), showing significant increases in the moduli and tensile strength.^578^ In particular, the films exhibited low coefficient of thermal expansion (Figure 34b).^336^ Biocompatible poly(vinyl alcohol) has been integrated with nanochitin in environmentally friendly films for improved performance.^308,579−581^

From the demand of green conversion, a series of composite films were proposed combining nanochitin and nanocellulose.^582^ For instance, an antifungal film resulted from a combination of ChNC and CNF via sequential processing with vacuum filtration and hot pressing (110 °C) at a curing pressure of 180 bar for 15 min. The main mechanism to inhibit fungi growth (*Arpergillus* sp.) originated from the amine groups in ChNC.^583^ Moreover, exploiting the oppositely charged nature of ChNF and CNC, their electrostatic interactions supported dense fibril packing during film formation, enabling all-biomass 2D materials with tunable barrier and mechanical properties.^584^ Compared to neat ChNC or CNC films, the ChNF/CNC composite films exhibited lower O_2_ permeability (1.4–1.9 cm^3^ μm/m^2^/d/kPa) and increased strain at break, indicating their potential as packaging materials. The low permeability was attributed to structural effects within the films originating from the aggregation of ChNF with CNC, driven by electrostatic attraction and H-bonding. Two possible mechanisms were proposed related to film formation: (1) dense layered lamellar structures with an increased O_2_ diffusion time scale and (2) long diffusion length scales by interfibril tortuosity within the lamellae.

Beyond simple mixing of nanochitin with other components, structural design into complex systems has been considered in composite films.^585^ One example is that of oily films using nanochitin-based Pickering emulsions, wherein nonvolatile oils act as the dispersed phase, stabilized by the nanochitin, also acting as matrix in the continuous phase.^586^ Nanochitin-derived oily films are similar to those produced from nanocellulose but with added bioactivity for active packaging applications.^587−589^ For instance, ChNC-based transparent oily films containing paraffin and starch were fabricated using ChNC-stabilized paraffin-in-water Pickering emulsions, where the starch acted as the comatrix in the continuous phase.^590^ The critical factor leading to oily films was the use of nanochitin as the stabilizer via the Pickering mechanism, allowing molding and drying upon film formation. Through controlling the structure and formulation of Pickering emulsions, the properties of nanochitin-based oily films can be adjusted. For instance, better barrier properties and enhanced bioactivity can be achieved by reducing the oil droplet size. However, these effects come with a cost since drying the emulsion to form the film involves coalescence of oil droplets. Moreover, the plasticizing effect of the oil phase may impair the mechanical performance of obtained films, which negatively affects the development of barrier properties and water resistance. Another emulsion route for structured composite films relates to Pickering emulsion polymerization.^483^ Nanochitin-stabilized monomer droplets, the dispersed phase, can be polymerized into nanochitin-based polymer dispersion, facilitating film formation.^591^ For example, a ChNF-stabilized Pickering emulsion system containing UV-curable acrylated epoxidized soybean oil (dispersed phase) was considered (Figure 34c1), where the oil phase was further UV-cured to form ChNF-based nanocomposites with well-preserved spherical-like microstructures (Figure 34c2).^592^ After water evaporation, yellowish but translucent thin composite films were obtained with an increased stiffness and strength (Figure 34c3).

Owing to their high transparency and robustness as well as chemical and structural versatility nanochitin composite films have been utilized in a range of applications. Novel packaging materials can be manufactured given the inherent biological activities of nanochitin.^593^ For instance, immobilization of anthocyanins in ChNC-based composite barrier film, in association with the targeted interaction or release of anthocyanins, endowed controlled color changes upon exposure to environments containing volatile components or with changing pH.^594,595^ This is the most useful in intelligent food packaging systems that require active responsiveness and freshness monitoring. By immobilizing active molecules, a miniaturized, optical, easy-to-use sensing bioplatform was developed by embedding/immobilizing various functional components. They included transparent, biocompatible, flexible ChNF nanopapers combined with plasmonic (silver and gold nanoparticles) and photoluminescent (quantum dots, carbon dots, and up-conversion systems) nanoparticles as well as colorimetric curcumin (Figure 34d1).^551^ The integration of such components was achieved via laser printing onto dried ChNF nanopaper in several forms: 2D multiwall patterns with hydrophobic walls and hydrophilic test zones (Figure 34d2), without compromising flexibility (Figure 34d3). Various model analytes were tested, confirming the efficiency and applicability of fabricated ChNF nanopaper-based sensing bioplatforms.

#### 5.3.3 Nanochitin Coatings

Coating is an efficient strategy to modify the surface of a substrate, e.g., by using polymers, colloids, or molecules.^596^ Coating allows multilayered constructs, enabling customized design of materials with desired functions.^597^ To fabricate nanochitin-based coatings, it is important to select a proper coating technique. For nanochitin alone, rod-coating produces homogeneous ChNC layers on polypropylene.^432^ Micropatterning of ChNC coatings onto cellophane films was achieved by inkjet printing of a ChNC aqueous dispersion. This was an efficient route to achieve functional biomedical systems, control over cellular shapes, precise monitoring of molecular events, and drug screening.^598^ On the other hand, spray-coating offers enhanced drying rate because of the large surface area of droplets under the contactless delivery of material on the substrate surface. Dip-coating that relies on alternative immersion and rinsing is used for the generation of multilayered nanochitin/nanocellulose nanocomposite coatings on various substrates.^552,599^ Accordingly, compared with coatings made from ChNF and CNC alone, the multilayer structures are more effective in reducing the O_2_ and water vapor transmission together with a low haze. These properties are associated with generation of densified multilayer barrier by electrostatic interfacial adhesion and bridging the nanofibers and nanocrystals.

The reported coating techniques represent a toolbox to create nanochitin coatings that can be precisely designed and synthesized for a range of functional applications.^553^ For instance, a superhydrophilic, antifouling membrane was developed by coating ChNC onto electrospun cellulose acetate membranes. The coating process was achieved by filtrating a ChNC suspension with the electrospun web, which was used as filter.^600^ This ChNC-coated membrane exhibited high water flux and low (bio)fouling, useful in water filtration. Superamphiphobic coatings based on nanochitin have been shown for uses that demand self-cleaning and antifouling effects.^601^ ChNC with reduced surface free energy was achieved by modification with fluorinated chains, through thiol linkages, which was used for self-cleaning and antifouling coatings.^339^ They were robust against mechanical and thermal treatments. Different liquid droplets formed quasi-spherical shapes on the coating, with high contact angles (>156.2°) and low roll-off angles (<9.3°). More importantly, such ChNC-based superamphiphobic coatings did not depend on the characteristics of the support: good resistance and repellency were realized on nonwoven fabrics, glass, sponge, plastic membranes, cotton, and filter paper. Surface coating enables antireflection and antifogging, useful in a wide variety of optical technologies, which reduces light reflectance loss and hence maximizes light transmission.^602,603^ A novel ChNF-derived antireflection coating was developed using an aqueous-based layer-by-layer self-assembly method involving sequential immersion and rinsing.^604^ During coating, an increased number of air voids inside the coating led to a low RI, which was derived from ChNF stacking, and precise control over the pH of ChNF and PAA led to systems with a low RI, which resulted in high-quality antireflection performance. Moreover, the durable coating was antifogging even at −20 °C, owing to the hydrophilicity of the ChNF and strong inter/intraparticle H-bonding.

A transparent and flexible gas barrier coating was demonstrated for food packaging by spray-assisted layer-by-layer nanoscale assembly of ChNC and CNF on PET films.^605^ The interplay between the oppositely charged nanomaterials afforded effective ionic cross-linking within the multilayer, forming water-resistant and mechanically stable coatings. Owing to their crystallinity and dimensions, the ChNC/CNF coating minimized the number of defects (which promoted gas permeation), resulting in outstanding barrier performance. The coating reduced the haziness of PET with a negligible loss of transparency and provided effective inhibition of antibacterial growth. More importantly, the radiofrequency transparency of the coating was shown by performing an identification tag reading test (Figure 34e1). As a proof-of-concept, the tag covered by nonconductive ChNC/CNF-coated PET was readable as a green light (Figure 34e2), whereas the tag remained red through conductive aluminum-coated PET (Figure 34e3), which indicated electromagnetic shielding. These results show the promise of nanochitin-based coatings in next-generation intelligent packaging materials.

### 5.4 Gels and Three-Dimensional Structures

Nanochitin serves as a suitable building block to support 3D constructs, mainly as hydrogels and aerogels/cryogels.^606^ Structured gels can be derived from an aqueous nanochitin suspension through a range of concentration methods, e.g., dialysis and neutralization, pH adjustment, crystal transformation, and exchange, among others.^19,245,607−609^ Composite gels that include nanochitin and other components have been developed for multiple functions.^610−612^

#### 5.4.1 Nanochitin Hydrogels, Aerogels, and Cryogels

**Figure 35:**
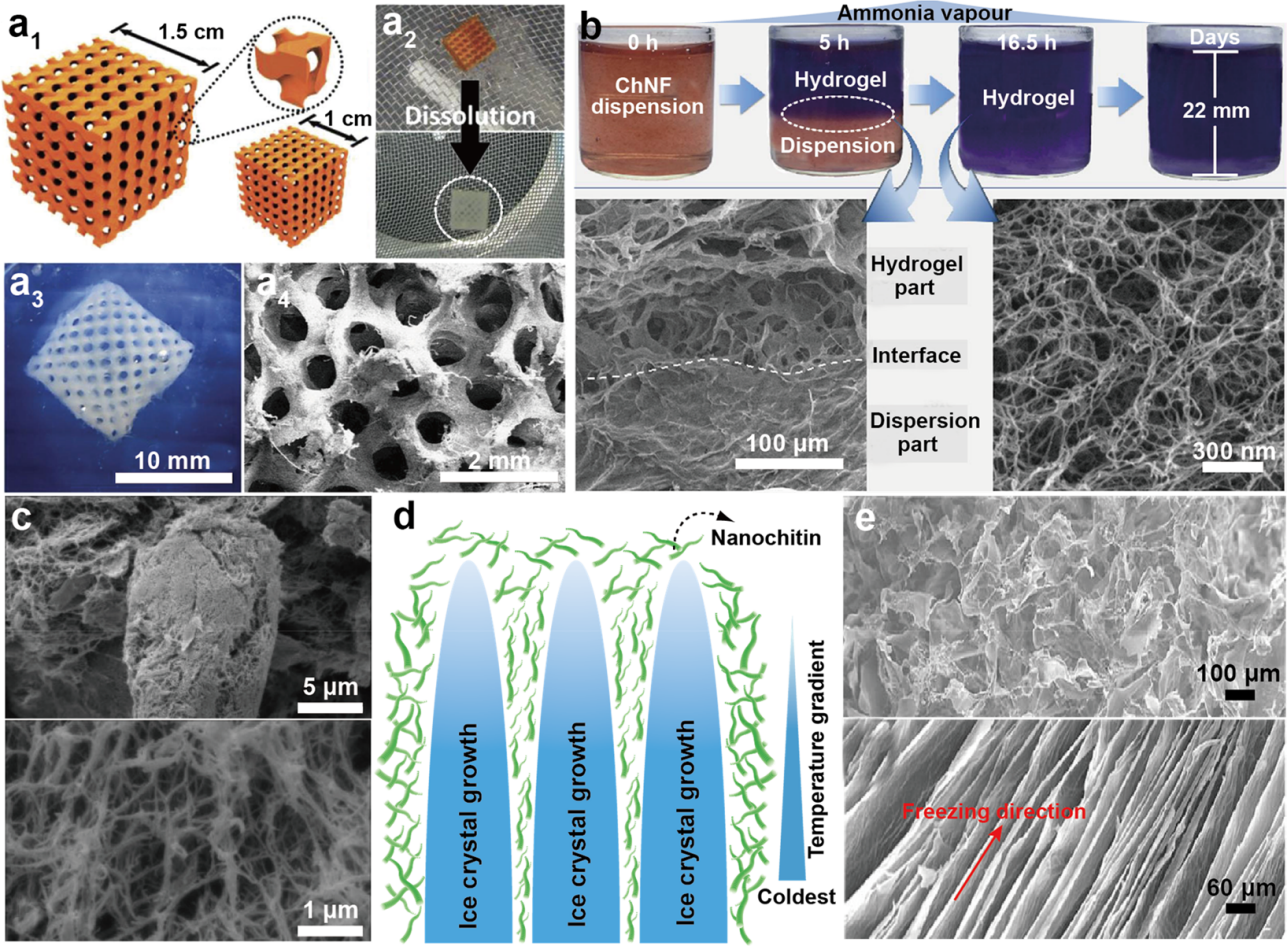
(a1) Structure of a scaffold used as a reverse template to form ChNF hydrogels. (a2) Visual appearance of the dissolution in alkaline media of the sacrificial template to form the ChNF hydrogel. (a3) Photograph and (a4) SEM image of ChNF hydrogel scaffold with a 10 mm edge length. Adapted with permission from ref (618). Copyright 2015 John Wiley and Sons. (b) Gelation process involving ChNFs in the presence of ammonia vapor during given processing time. Following a limited gelation period (5 h), a clear interface between the upper hydrogel phase and the bottom dispersion phase coexists (SEM image, bottom left); after ChNF dispersion is fully gelled (16.5 h), a network structure is formed by the nanofibers (SEM image, bottom right). Adapted with permission from ref (621). Copyright 2020 Springer Nature. (c) SEM images at higher magnification of a ChNC aerogel where the upper panel shows a porous network and nanocrystal aggregation with the bottom panel showing a highly porous network. Adapted with permission from ref (614). Copyright 2013 John Wiley and Sons. (d) Schematic illustration of the directional freezing used to prepare nanochitin cryogels. (e) SEM images of the cross section of freeze-dried ChNF cryogels, with the upper and bottom panels indicating ChNF cryogels obtained by freezing at −80 °C and under liquid nitrogen, respectively. Adapted rom ref (554). Copyright 2014 American Chemical Society.

Nanochitin can be assembled into hydrogels by physical cross-linking, e.g., chain entanglement, hydrophobic interaction, and H-bonding.^18^ Owing to its morphological and chemical features, ChNF has been used to produce hydrogels by forming strong, robust 3D networks at low concentration.^273^ For instance, gelation took place in a ChNF suspension at 0.4 wt % concentration, while ChNF hydrogels exhibiting a high storage modulus were achieved by increasing the suspension concentration to 3.0 wt %.^613^ With the assistance of low-power ultrasonication (similar energy magnitude as that of H-bonding, 4–50 kJ/mol),^25^ ChNCs assembled into 3D-percolated networks through H-bonding.^614^ Self-sustaining, durable β-ChNF hydrogels were fabricated by hydrothermal treatment, where the elevated temperature improved hydrophobic interactions between the nanofibers, fostering ChNF self-assembly into hydrogels.^615^ Partial deacetylation of nanofibrillated ChNF, which activates surface amino groups, resulted in colloidally stable ChNF suspensions. Hydrogels were prepared from such ChNF suspensions by directly adjusting the pH to >7^616^ under the influence of surface charge screening.^617^ However, this procedure often does not achieve good control of the homogeneity, interfibrillar interactions, and bridging during gelation. To avoid this issue, a gyroid hydrogel scaffold made from a ChNF suspension was developed by the “reverse templating” approach.^618^ The sacrificial template was prepared by stepwise lithographic 3D printing of a mixture of methacrylates and acrylamides, which contained methacrylic anhydride as a hydrolytically labile cross-linker (Figure 35a1). After filling the void space of the template with a ChNF hydrogel, the template was dissolved in alkaline media (Figure 35a2), resulting in a ChNF system that retained the visual appearance of the original design (Figure 35a3) and led to an aligned internal porous structure (Figure 35a4). On the other hand, in order to control the interactions leading to nanofiber interconnections, a gas phase coagulation was applied by exposing the system to a volatile alkaline (e.g., ammonium hydroxide) or acid (e.g., hydrochloric acid) vapor, leading to physically cross-linked ChNF hydrogels containing PD-ChNF or TEMPO-ChNF (Figure 35b).^619−621^ Besides hydrogels produced from pure nanochitin, blending of nanochitin with other components allows composite hydrogels with enhanced performances.^622,623^ For instance, loading alginate into a ChNC suspension improved the mechanical properties and swelling stability of the hydrogels due to strong electrostatic interactions between the nanocrystals and alginate molecules.^624^

Although physical cross-linking generates nanochitin hydrogels with robust structures and strength, further enhancement of the performance and functions of nanochitin-based hydrogels requires chemical cross-linking, leading to covalently entangled networks.^625^ In most cases, particularly for PD-ChNF, the cross-linking reaction takes place at the amino groups. For instance, high-axial-ratio ChNF was produced by the microfluidization of partially deacetylated chitin and were cross-linked with glutaraldehyde at various concentrations. By using an ice templating method, and through freezing and thawing, ultrastrong, highly viscoelastic, and shape-recoverable hydrogels were formed even at a ChNF concentration as low as 0.4 wt %.^626^ The obtained hydrogel retained its integrity after a number of dehydration (compression) and swelling (immersion) cycles, which demonstrates the advantage of covalent cross-linking to generate high-performance nanochitin hydrogels. Recently, inspired by a naturally occurring quinone hardening process during insect cuticle sclerotization, a quinone-cross-linked hydrogel was produced from surface-deacetylated ChNF in neutral aqueous phase.^625,627^ Therein, covalent cross-linking occurred between the amino groups present on the surface of ChNF and the quinone rings, derived from the oxidation of phenol.^622^ The obtained hydrogels displayed a nearly 10-fold higher tensile strength compared to the hydrogels formed by nondeacetylated ChNF, indicating the extended cross-linking between quinone and amino groups. Genipin, a natural product extracted from gardenia fruit, has shown low cytotoxicity and has been demonstrated to efficiently cross-link β-ChNF, forming strong hydrogels.^615^ The most significant improvement of genipin-cross-linked ChNF hydrogels was that they maintained their original shape in highly concentrated urea, realizing a strong resistance to chemical degradation.

A sol–gel transition can occur in nanofibril suspensions subjected to exchange of water with miscible organic solvents, e.g., acetone, ethanol, etc.^628^ Further removal of the solvent in the produced organogel leads to aerogels with given 3D network structures. Following this concept, a mesoporous ChNC aerogel has been produced via ethanol substitution followed by supercritical CO_2_ drying of the organogel.^614^ The supercritical drying retained the mesoporous network in the dried ChNC aerogel (Figure 35c), following shrinkage that can be as small as 4%. Such aerogels exhibit low density (0.043 g/cm), high porosity (up to 97%), large specific surface area (261 m^2^/g), and excellent mechanical performance (modulus up to 9.3 MPa). The obtained lightweight materials offer promise for thermal insulation, catalysis, and biomaterials. In addition to aerogel formation from supercritical drying, a dried nanochitin gel can be produced from freeze-drying of the hydrogel, yielding a cryogel.^629,630^ For instance, a TEMPO-oxidized nanochitin hydrogel was subjected to sequential chemical and physical cross-linking followed by freeze-drying into a cryogel with tunable internal structures.^631^ The main differences between a cryogel and an aerogel relate to the final internal 3D network that develops from the nanochitin gel.^632,633^ The direction of ice crystal growth significantly influences the alignment of nanofibers and the formed porous structures.^626^ For instance, an aligned porous ChNC cryogel was produced by directional freeze-casting.^634^ This is because hexagonal water ice crystals exhibit strong anisotropic growth kinetics, varying over 2 orders of magnitude as far as crystallographic orientation. The freezing process is easier for crystals that exhibit parallel growth in the direction of temperature gradient, thereby generating oriented pores, which depends on the solidification conditions used.^635^ Thus, when the bottom of a hydrogel is in contact with liquid N_2_, ice crystals grow vertically along the direction of the thermal gradient (Figure 35d). Moreover, adjusting the freezing rate allows control of the formation of the microstructure formed from the ChNF cryogel, ranging from oriented sheets to 3D aperiodic nanofiber networks (Figure 35e). The latter was shown to be similar in size and interconnectivity to that of the cuticle of the white *Cyphochilus* beetle.^554^

In sum, a number of methods have been used to produce nanochitin gels. The limited mechanical strength of these gels is a drawback, particularly in the case of nanochitin cryogels obtained upon drying. Hence, covalent cross-linking has been used as an option to improve the strength of the hydrogels, noting that it carries a counteracting effect, namely, reduced control of the microstructure. Taken together, it is critical to nondestructively balance the formation and chitin nanoparticle networking along with the methods used to transfer 3D constructions into the dried counterparts.

#### 5.4.2 Emerging Applications of Nanochitin Gels

As introduced previously, nanochitin brings various beneficial characteristics: versatility, biocompatibility, biodegradability, low immunogenicity, and antibacterial activity. In this subsection, we summarize emerging applications of nanochitin-based hydrogels, including those related to the biomedical, energy and environmental fields.

**Figure 36:**
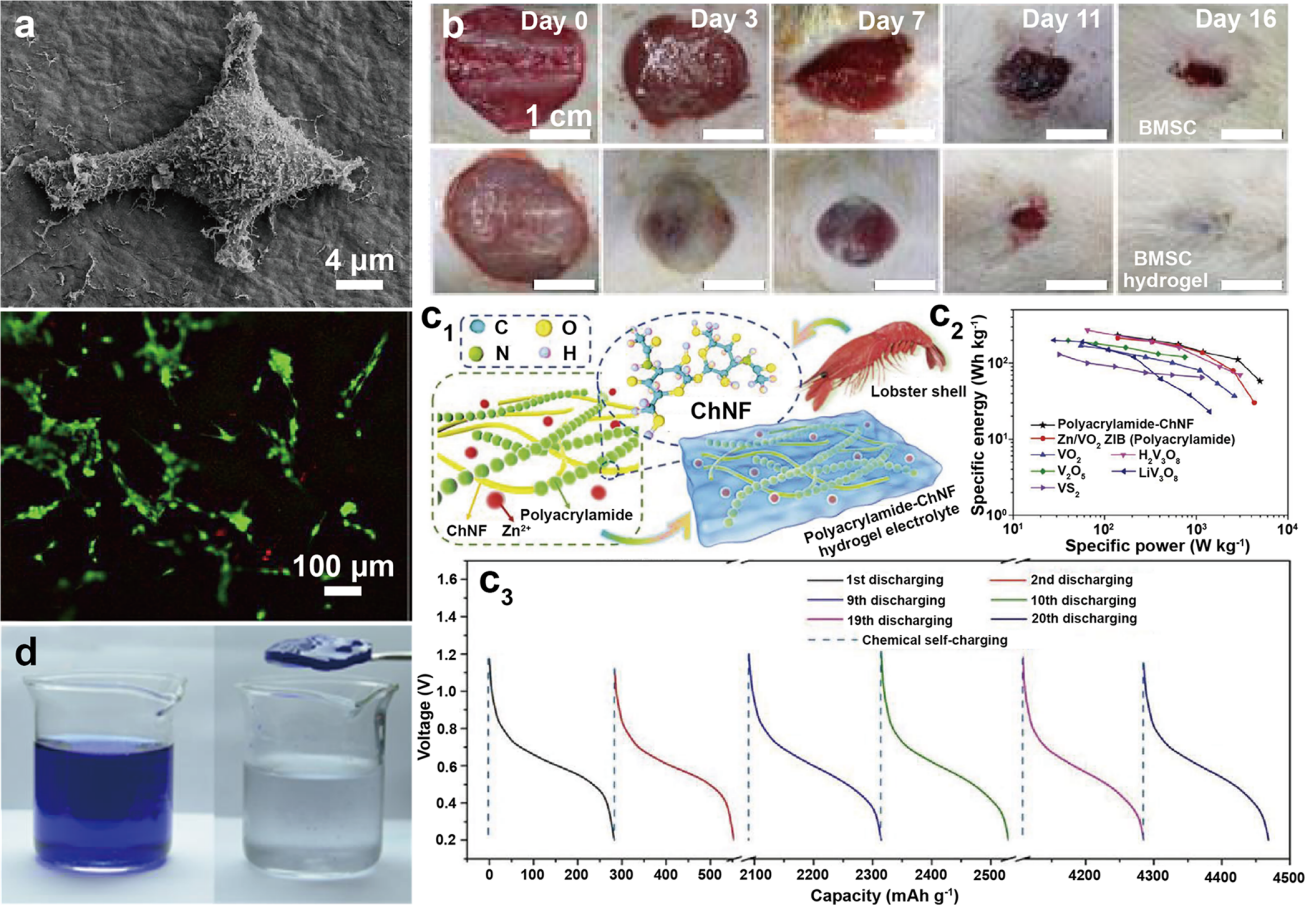
(a) SEM image (upper panel) and fluorescent micrograph (bottom panel) of osteoblast cells on alginate-containing ChNC hydrogel (ChNC/alginate ratio of 1). Adapted from ref (624). Copyright 2015 American Chemical Society. (b) Representative photos of cutaneous wounds treated with BMSCs alone and BMSC-encapsulated ChNF hydrogel, indicating the accelerated healing at all time points after incorporating BMSCs in the ChNF hydrogel. Adapted with permission from ref (644). Copyright 2018 John Wiley and Sons. (c1) Schematic showing of the preparation of a polyacrylamide-ChNF hydrogel that is suitable as the electrolyte in solid-state ZIB containing a VO_2_ cathode. (c2) Ragone plot of a Zn//VO_2_ solid-state battery using polyacrylamide-ChNF hydrogel electrolyte compared with other vanadium-based cathodes for an aqueous ZIB. The results indicate the high energy density of the current system. (c3) Galvanostatic discharging and chemical self-charging (oxidation for 6 h) cycles of solid-state ZIB. Twenty cycles were performed continuously, showing excellent self-rechargeability. Adapted with permission from ref (652). Copyright 2021 John Wiley and Sons. (d) Visual appearance of dyed water before and after treatment with a ChNF hydrogel. Reactive Blue 19 (90 mg/L) was fully adsorbed by the ChNF hydrogel (pH 1.5 after 12 h contact at 150 rpm stirring). Adapted from ref (620). Copyright 2016 American Chemical Society.

Nanochitin gels have been widely used in biomedical applications, for instance, in tissue engineering, wound dressing, drug delivery, antimicrobial patches, etc. This is derived from chitin’s biocompatibility, biodegradability, and nontoxicity as well as its processability into given material shapes, such as sponges and scaffolds.^610,636^ For instance, gas-phase coagulation of PD-ChNF hydrogels containing glycerophosphate led to porous and stable structures that can be used as delivery vehicles for prolonged release of microemulsions.^637^ Tissue engineering based on hydrogels belongs to the interdisciplinary fields of life sciences and engineering, opening the way for systematic designs. The latter are used with living cells to process biological substitutes for body implantation and/or as a guide for tissue regeneration.^638^ Nanochitin gels have attractive chemical, morphological, and mechanical advantages for applications in tissue engineering,^639^ particularly because they can be easily molded into different geometries to support the attachment and proliferation of cells (*e.g*., osteoblasts and others).^640^ Typically, ChNF hydrogels are used as templates for the mineralization of calcium phosphate crystals, leading to bone tissue engineering with improved cell adhesion and osteointegration.^641^ Mineralized ChNF hydrogels accelerated the differentiation of osteoblasts in subcutaneous tissues of rats. Meanwhile, osteoblasts were found surrounding the areas of mineralization, wherein fibroblasts and collagen were distributed tightly in the hydrogel. Additionally, incorporating alginate with ChNC produced a nanocomposite hydrogel with improved adhesion and proliferation of osteoblast cells (Figure 36a).^624^ This is associated with a hierarchical inner structure significantly enhancing the mechanical strength and inhibiting swelling of ChNF-based hydrogels.

Besides tissue engineering, the high stability and superior antibacterial properties of nanochitin hydrogels make them suitable as wound healing materials.^642,643^ For instance, cell-encapsulated ChNF-based hydrogels induced differentiation of bone marrow mesenchymal stem cells (BMSCs) in the absence of inducers.^644^ The ChNF-based hydrogel protected BMSCs from elimination by the harsh wound microenvironment, eventually enhancing cutaneous wound regeneration (Figure 36b). In another example, a 3D ChNF-based porous sponge, derived from a quaternary chitin/PD-ChNF hydrogel, was cross-linked by glutaraldehyde and was effective in hemostasis of noncompressible wounds.^645^ The underlying effect benefited from outstanding water absorbency, water-trigged shape recovery, and mechanical strength combined with antibacterial activity, cytocompatibility, and hemocompatibility. Hence, the sponges can be delivered into the wound cavity in the shape of a small patch that expands in volume upon contact with blood, filling the wound. This effect attracts and stimulates blood cells/platelets that are positively charged,^646^ thus promoting blood clotting. The use of noncompressible wound models in vivo, using rat livers, has shown superior hemostatic performance compared to the traditional hemostatic materials (medical gauze and gelatin sponges).

In a different type of application, N-doped electrochemical materials have been reported.^647,648^ Structurally, nanochitin hydrogels bear interconnected and robust network structures, which can be transferred into multilevel pores in aero- or cryogels, leading to hierarchically structured porous matrices.^649^ Thus, taking advantage of these benefits, nanochitin gels have been shown in catalysis, electrochemical, and energy conversion devices. For instance, ChNF aerogels and their exposed C2 amines displayed great catalytic activity and excellent cycling stability toward the Knoevenagel condensation reaction between benzaldehyde and ethyl cyanoacetate.^616^ The catalytic performance was even better than that measured in chitosan aerogels. The nonconductive nature of chitin can be addressed by introducing carbon nanotubes, producing conductive, mechanically robust, and self-standing in 3D porous structures from ChNF hydrogels.^650,651^ Using the structural advantage of ChNF hydrogels, a polyacrylamide-ChNF nanocomposite hydrogel was proposed as an electrolyte holder for a chemically self-charging, flexible solid-state zinc ion battery (ZIB) using a vanadium dioxide (VO_2_) cathode (Figure 36c1).^652^ With a power density of 139.0 W kg^–1^, the ZIB delivered an energy density of 231.9 Wh kg^–1^ (Figure 36c2). The superior electrochemical performance of the ZIB was partially attributed to the entangled network and robust tunnel structure of ChNF-based hydrogel electrolytes, which offered efficient pathways for ion diffusion during operation. Remarkably, the designed ZIB exhibited chemically self-charging capability via redox reactions between the cathode and oxygen. After oxidation for 6 h in air, the ZIB delivered a high discharging capacity, 263.9 mAh g^–1^ at 0.2 A g^–1^ (Figure 36c3). With the assistance of a small amount of acetic acid in the electrolyte hydrogel, 20 cycles of galvanostatic discharging and chemical self-charging were achieved without loss in electrochemical performance.

Taking advantage of the abundant active functional groups, including primary amino, hydroxyl, and acetamido groups, chitin and nanochitin display great adsorption capacity;^653^ hence, they have gained attention as effective biosorbents for the removal of various types of pollutants.^553,654^ Therefore, exploiting such chemical advantages associated with structured internal networks (pores) of nanochitin gels, they are highly promising in environmental remediation, typically as filtration materials for water purification and for contaminant removal.^655^ For instance, ultralight (0.2 wt %) yet robust, self-standing ChNF hydrogels were effective for dye removal (reactive Blue 19) from polluted water (Figure 36d), suggesting the use of ChNF-based hydrogels as a suitable biosorbent.^620^ Composite nanochitin gels have shown even better adsorption capability.^481,656^ A biohybrid cryogel made from PD-ChNF and TEMPO-CNF was prepared by self-assembly, driven by electrostatic forces and H-bonding, exhibiting a very high adsorption capacity (217 mg g^–1^ for As(III) under neutral pH conditions and 531 mg g^–1^ for methylene blue under alkaline aqueous conditions), which followed rapid adsorption kinetics.^606^ The results were explained by the highly porous and interconnected structure of the composite cryogel. Moreover, the respective cryogel was reusable, exhibiting a high methylene blue adsorption capacity (505 mg g^–1^ even after five successive adsorption–desorption cycles). A recent report indicates the use of ChNF cryogels decorated with silica toward superhydrophobic adsorbents for underwater collection of methane.^657^ The underlying mechanism of such an action of the ChNF cryogel relates to the formation of a silver mirrorlike film, making possible the entrance of methane gas through the pores. No such film was formed with pure ChNF, which resulted in aggregation of methane bubbles underneath the hydrophilic surface.

## 6 Industrialization Perspectives

In addition to the renewability, sustainability and unique chemical characteristics, the resource-efficiency and cost-competitiveness of nanochitin should be considered. Mature industrial-scale large-volume production usually lowers manufacturing costs; however, the physicochemical properties determining the design, processing, and application of industrialized nanochitin are not necessarily consistent. The market acceptability of nanochitin is also of importance for commercialization. Finally, the effectiveness of nanochitin and translating biomass waste to ecofriendly bioproducts are key factors dictating future industrial opportunities.^658,659^ Bearing these considerations in mind, this section introduces some industrialization perspectives (Figure 2, panel 6).

### 6.1 Industrialization Opportunities

#### 6.1.1 Source of Feedstocks

The type of chitin feedstock relates to the performance and manufacturing cost of nanochitin as well as its price. Different chitin grades require given extraction strategies, resulting in associated process efficiencies and energy consumption levels. More importantly, chitin sources together with processing technologies directly impact the chemistry and structure of chitin and subsequent nanochitin, which ultimately affect functionality and application. Consequently, the structure–process–property relationships between chitin sources and applications should be considered. Crustacean shell waste represents approximately half of the total weight of shellfish,^660^ which is considered to be the main chitin source. However, other sources include squid (β-chitin),^248^ which has unique advantages. For instance, the squid pen is easily processed and the isolated nanochitin has a large aspect ratio, enabling wide tunability as far as the flow behavior at given solid content, making it more competitive as functional additives.^557^

#### 6.1.2 Industrial Production

**Figure 37:**
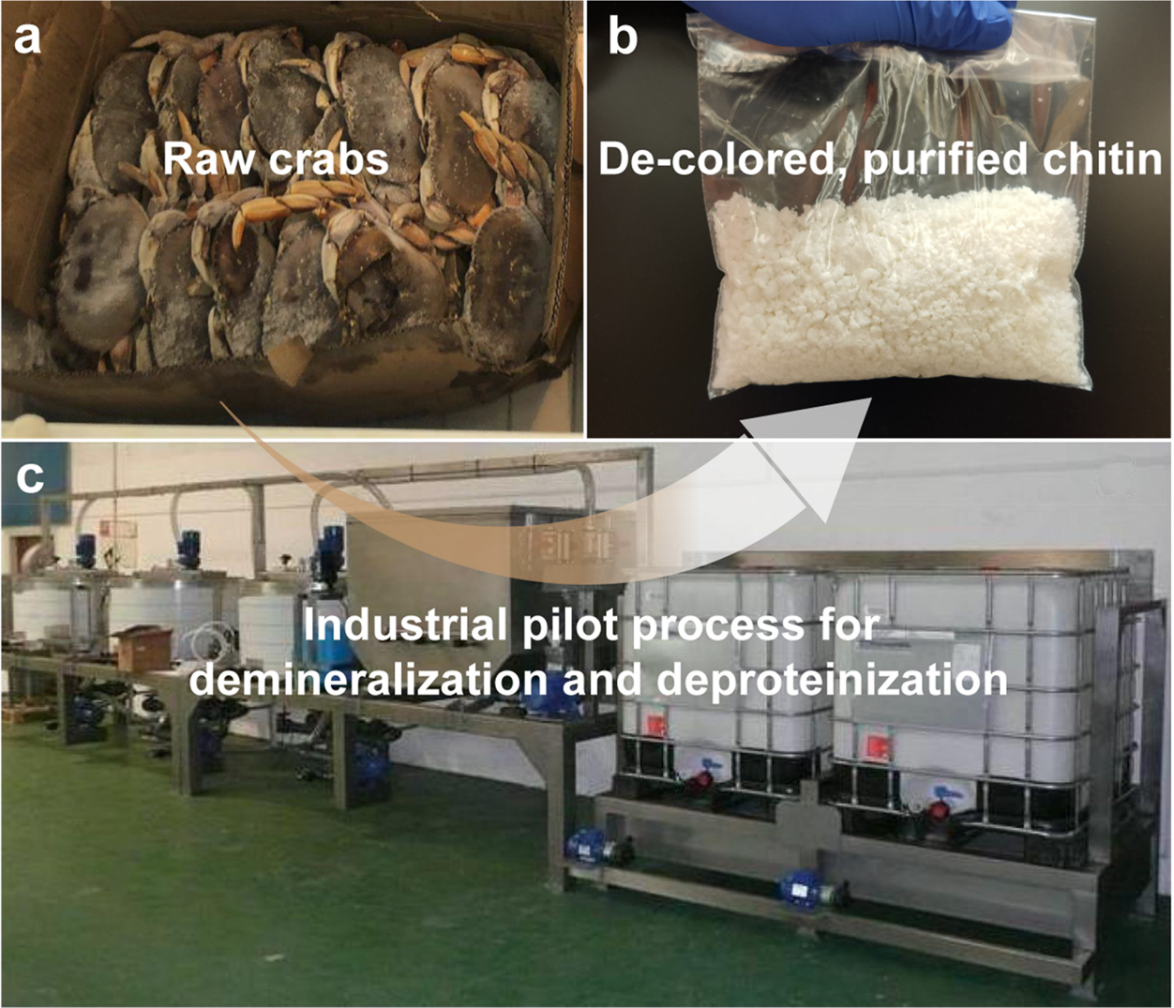
Industrial processing of (a) raw crab shells into (b) decolored, purified chitin flakes using (c) a pilot production system that removes the minerals, proteins, and surface pigment components of the crab shells. Adapted with permission from ref (661). Copyright 2017 MDPI.

While cellulose is currently produced worldwide in the context of the pulp and paper industry, industrial processing of chitin lags behind.^10^ Commercialization of nanochitin has been favored by recent state-of-the-art technologies that allow processing of chitin into its nanosized structures. With these developments, pre-production of raw chitin involves DM, DP, and decoloring (Figure 37). Processing of chitin into nanochitin has been achieved over the last two decades in pilot plants operating at high pressure and temperature but under cost-effective conditions, using chemical, enzymatic, or combined approaches.^290,661^ Industrial production still requires manufacturers to consider the chemical composition of the source feedstock, including minerals and proteins as well as the interactions that exist in the hierarchical chitin structures and their effect on the selection of processing conditions. The purity of the produced nanochitin significantly impacts the manufacturing costs and properties as well as commercialization; for instance, this includes the chitosan/chitin content, depending on the DDA. In industrial production, over-reaction leads to the formation of chitosan; meanwhile, depending on the application, high-purity nanochitin (chitosan-free) does not necessarily perform better than low-purity mixtures.^662^ It should be noted that due to the reduced processing involved, products containing nanochitin/chitosan may be more cost-competitive compared with pure nanochitin. The manufacturing cost of nanochitin also depends on the target nanochitin morphology, *e.g*., size and size distribution, degree of nanofibrillation, *etc*. For example, nanochitin with smaller dimensions and higher nanofibrillation degrees entails a higher cost compared with the microfibrillated forms. The trade-off between performance and cost should be considered in the selection of nanochitin.

Nanochitin processing method, storage, transport, purity and intended use are all important considerations in efforts to scale-up production. This leads to the key component of nanochitin: water. Nanochitin is typically extracted in three different forms, considering the water content: (1) as a suspension with a solid content <1 wt %; (2) as a wet gel with a solid content of 2–10 wt %; and (3) as a dry powder. The water content may be adjusted to different levels by using additional processing, including spray-drying. To facilitate storage and transport, nanochitin in the dry powder form is preferred. For applications such as solvent-based plastics, textiles, and composites, dry powder is required, given the lack of water dispersibility. By contrast, most of the applications relating to nanochitin for biomedical applications usually favor its hydrated form, *i.e*., a wet nanochitin gel that can be easily incorporated into the given system prior to use.

#### 6.1.3 Consumer Acceptability

Even if nanochitin is used as functional component in industry, its applications may be limited by the lack of consumer acceptance.^663^ Consumers must balance the potential functional benefits provided by nanochitin with any potential risks. Consequently, there is a need to understand what consumers perceive as beneficial and how they construe risks.^664^ However, consumers often make decisions based on public opinion that may unintentionally highlight the potential risks of nanoparticles, with less attention to details and the science involved. This specially applies to nanopolysaccharides that are potentially safe. Another aspect is that of biodegradation: due to a preference for the acetylated sites, some families of enzymes, *e.g*., chitotriosidases (chitinases), degrade more quickly chitin nanoparticles than the chitosan polymer.^665^ To facilitate the industrialization and commercialization of nanochitin, public awareness and acceptability of nanochitin should be carefully considered, requiring the development of holistic and integrated approaches.

### 6.2 Bioeconomy Vision

Beyond the advantages described in previous sections, a strong driver for a future sustainable society, for instance, in developing and utilizing chitin and nanochitin, is the shift from petroleum-based materials toward a circular economy and, ultimately, to a circular bioeconomy.^32,666−668^ While such a shift requires deep transformations,^2^ including fundamental changes in policies, technologies, organization, social acceptance, and markets, nanochitin offers great opportunities that fit many urgent needs. For instance, due to the vast sources and rapid regenerative practices in the crustacean industries, the production and utilization of nanochitin provides a balanced use of marine resources, safeguarding the biodiversity of the planet. The seafood waste–nanochitin nexus stimulates the reuse, recycling, and upcycling of crab shell waste and byproducts, minimizing the loss of natural resources. Moreover, taking advantage of advancements in nanochitin chemistry and technologies, such waste can be transferred to ecofriendly, high-value-added products *via* simple, scalable, and cost-effective routes. For example, nanochitin shows great promise as a replacement of some oil-derived plastics and chemicals, similarly to nanocelluloses.^669 −671^ Finally, relatively high conversion rates, going from chitin to nanochitin, enable low emissions of non-essentials, which prevents the unnecessary exploitation of low-valued byproducts, particularly alleviating the massive impact of production that is unlikely to be offset by recycling. In sum, we anticipate that the prominent features of nanochitin will expand the development of a biobased material, making it an additional enabler for the future circular bioeconomy.

## 7 Summary and Perspectives

The breadth, diversity, and scope of recent research associated with nanochitin have witnessed an expansion over the past two decades. Chitin and its nanoform, nanochitin, have attracted great interest; simultaneously, materials innovation from aquaculture offers a solution to many of the issues related to agricultural land use. Despite the fact that nanochitin shares similar morphological features compared to other nanopolysaccharides but bear distinctive chemical feaures, research advances in the area of nanochitin are still lagging behind plant-based nanocelluloses.

This Review summarized the fundamental chemical and biological aspects of chitin, highlighting the prominent role of chitin in constructing hierarchically multileveled structures in living organisms. The most distinctive characteristic of chitin is that it contains nitrogen, which can lead to cationic moieties, a desirable feature in a number of applications. The isolation of varying axial-aspect nanochitin depends on the chemical composition of chitin as well as the structures it forms, starting from the elementary chitin assemblies and going to higher orders or length scales. Based on the hierarchical structures of fibrous chitin, two types of mesostructured assemblies can be considered, nanofibers and nanocrystals, which can be isolated by top-down approaches, as discussed in this Review. The methods used in the isolation of chitin assemblies involve mechanical, chemical, and biological treatments, which directly impact the characteristics and application of the produced nanochitin. They are biocolloids that can be conveniently used in the construction of new materials. The assembly of nanochitin is paramount for achieving desirable performance, and therefore, we discussed the assembly forces and the interactions relevant to fibrillar nanoparticles and supramolecular assemblies dispersed in given media. Nanochitin provides an opportunity for new designs and inspires new strategies for material development, for instance, by regulating the self-assembly or by compounding with other materials. We considered a range of nanochitin-derived bioproducts in different dimensional formats (0D to 3D), emphasizing hierarchical structuring. Such materials display unique physicochemical properties and biological activity. Nanochitin is associated with anisotropy, high specific surface area, networking, nano-confinement, and optical effects. Such features are relevant to applications in the health, packaging, electronic, environmental, catalysis, and biomedical fields. We ended with a brief account of the production of nanochitin considering the feedstock, processing methods, and consumer acceptability.

Although great progress has been achieved to engineer nanochitin into sustainable and functional materials, many challenges remain related to processability and performance. Initiatives in this area require a deep understanding of the nature of interactions involving chitin and nanochitin. Efforts to retain the complex structure of nanochitin, upon isolation, are highly desirable, yet far from successful. New knowledge about the natural assembly of nanochitin is needed to facilitate fractionation and to open new uses. This requires the development of cost-effective, green processes that make full use of every feature encoded in nanochitin building blocks.

## List of Abbreviations

AA: Acrylic acid
APS: Ammonium persulfate
BMSCs: Bone marrow mesenchymal stem cells
ChNC: Chitin nanocrystal
ChNF: Chitin nanofiber
CNC: Cellulose nanocrystal
CNF: Cellulose nanofibril
Cryo-TEM: Cryogenic transmission electron microscope
Cryo-SEM: Cryogenic scanning electron microscope
DA: Degree of acetylation
DDA: Degree of deacetylation
DFT: Density function theory
DM: Demineralization
DP: Deproteinization
EDC: *N*-(3-(Dimethylamino)propyl)-*N’*-ethylcarbodiimide hydrochloride
EDL: Electric double layer
EISA: Evaporation-induced self-assembly
GIT: Gastrointestinal tract
H-bonding: Hydrogen bonding
HIPPE: High-internal-phase Pickering emulsion
INPC: Interfacial nanoparticle complexation
LC: Liquid crystal
LCP: Liquid crystalline phase
LNP: Lignin nanoparticles
MD: Molecular dynamic
O/W: Oil-in-water
PD-ChNF: Partially deacetylated ChNF
PEG: Poly(ethylene glycol)
PET: Poly(ethylene terephthalate)
POM: Polarized optical microscopy
RI: Refractive index
SAXS: Small-angle X-ray scattering
TEMPO: 2,2,6,6-tetramethylpiperidine-1-oxyl radical
TEMPO-ChNF: TEMPO-oxidized ChNF
TEMPO-CNF: TEMPO-oxidized CNF
VO_2_: Vanadium dioxide
vdW: van der Waals
W/O: Water-in-oil
ZIB: Zinc ion battery
